# Amino acid residues in five separate HLA genes can explain most of the known associations between the MHC and primary biliary cholangitis

**DOI:** 10.1371/journal.pgen.1007833

**Published:** 2018-12-03

**Authors:** Rebecca Darlay, Kristin L. Ayers, George F. Mells, Lynsey S. Hall, Jimmy Z. Liu, Mohamed A. Almarri, Graeme J. Alexander, David E. Jones, Richard N. Sandford, Carl A. Anderson, Heather J. Cordell

**Affiliations:** 1 Institute of Genetic Medicine, Newcastle University, Newcastle upon Tyne, United Kingdom; 2 Academic Department of Medical Genetics, University of Cambridge, Cambridge, United Kingdom; 3 Division of Psychological Medicine and Clinical Neurosciences, School of Medicine, Cardiff University, Cardiff, United Kingdom; 4 Human Genetics, Wellcome Sanger Institute, Wellcome Genome Campus, Hinxton, Cambridgeshire, United Kingdom; 5 Department of Forensic Science and Criminology, Dubai Police HQ, Dubai, United Arab Emirates; 6 Department of Hepatology, Cambridge University Hospitals National Health Service (NHS) Foundation Trust, Cambridge, United Kingdom; 7 Institute of Cellular Medicine, Medical School, Newcastle University, Newcastle upon Tyne, United Kingdom; University of Manchester, UNITED KINGDOM

## Abstract

Primary Biliary Cholangitis (PBC) is a chronic autoimmune liver disease characterised by progressive destruction of intrahepatic bile ducts. The strongest genetic association is with HLA-DQA1*04:*01*, but at least three additional independent HLA haplotypes contribute to susceptibility. We used dense single nucleotide polymorphism (SNP) data in 2861 PBC cases and 8514 controls to impute classical HLA alleles and amino acid polymorphisms using state-of-the-art methodologies. We then demonstrated through stepwise regression that association in the HLA region can be largely explained by variation at five separate amino acid positions. Three-dimensional modelling of protein structures and calculation of electrostatic potentials for the implicated HLA alleles/amino acid substitutions demonstrated a correlation between the electrostatic potential of pocket P6 in HLA-DP molecules and the *HLA-DPB1* alleles/amino acid substitutions conferring PBC susceptibility/protection, highlighting potential new avenues for future functional investigation.

## Author summary

Primary Biliary Cholangitis (PBC) is a chronic autoimmune liver disease that exhibits strong genetic associations, especially with variants in the human leukocyte antigen (HLA) gene region. Here we use dense single nucleotide polymorphism (SNP) data from the largest PBC study to date (2861 cases, 8514 controls) to investigate the likely underlying causes of this association, via performing imputation of HLA classical alleles and amino acids. We show that the HLA association can be largely explained by variation at five separate amino acid positions, one of which shows functional relevance to electrostatic potentials of HLA-DP molecules.

## Introduction

Primary Biliary Cholangitis (PBC; formerly known as Primary Biliary Cirrhosis) is a rare cholestatic liver disease characterized by progressive auto-immune destruction of intrahepatic bile ducts resulting in cholangitis, liver fibrosis and, eventually, cirrhosis. Candidate gene studies have consistently demonstrated association with polymorphisms in the human leukocyte antigen (HLA) region [1–8]. Genome-wide studies [9–12] have confirmed these HLA associations and have also identified 27 non-HLA risk loci. The MHC region, however, remains by far the strongest genetic contributor to disease susceptibility, with HLA haplotypes containing the HLA-DQA1*04:*01* allele conferring an approximately threefold increased disease risk [13].

To help understand the mechanisms underlying these HLA associations, and to identify functional, potentially causal, variants within the HLA region, we used previously-generated dense SNP genotype data from 2861 UK PBC cases and 8514 UK controls [13] to impute classical HLA alleles and amino acid polymorphisms within these 11375 individuals, using current state-of-the-art methods implemented in the software packages HLA*IMP:03 [14], HLA*IMP:02 [15], HIBAG [16] and SNP2HLA [17]. Previous interrogation of the UK PBC case/control data set risk [13] using classical HLA alleles imputed using the package HLA*IMP:01 [18, 19] had revealed four haplotypes showing independent disease associations: the well-established association at HLA-DQA1*04:*01* (which forms a haplotype with HLA-DQB1*04:*02* and HLA-DRB1*08:*01*), two previously identified protective effects marked by alleles HLA-DQB1*06:*02* [20] and HLA-DQB1*03:*01* [1], and a novel association marked by the haplotype HLA-DRB1*04:04/HLA-DQB1*03:*02*. Similar associations were also observed in application of HLA*IMP:01 to a smaller separate European data set [21]. Our updated analysis of the UK PBC data set, reported here, confirms these previously-observed associations, suggests potential additional independent associations, and suggests that the majority of the SNP and classical allele association in the HLA region can largely be explained by variation at five separate amino acid positions.

## Results

### Classical HLA associations

Various software packages have been developed for the imputation of classical HLA alleles (and, in some cases, amino acid substitutions) using dense SNP data; here we used the current state-of-the-art packages HLA*IMP:03 [14], HLA*IMP:02 [15], HIBAG [16] and SNP2HLA [17], and compared the results obtained for classical HLA alleles with those previously obtained [13] using HLA*IMP:01 [18, 19]. Our rationale for using four different software packages was the fact that the precise methodology implemented varies across the different packages, as do the reference sets used to inform the imputation. Thus, we were interested in examining the sensitivity of our findings to the software implementation used, with concordance of findings seen across different software implementations providing a greater degree of confidence in the results obtained.

Analysis of the UK PBC data set using these packages confirmed the previously-observed associations [13, 21] seen with classical HLA alleles (Table 1, haplogroups 1–4) and suggested potential additional novel independent associations at *HLA-DPB1* (HLA-DPB1*03:*01*, HLA-DPB1*06:*01*, HLA-DPB1*04:*01*, HLA-DPB1*10:*01* and HLA-DPB1*17:*01)*, *HLA-C* (HLA-C*04:*01*) and *HLA-DPA* (HLA-DPA*02:*01*) (Table 1, haplogroups 5–10). Results were largely concordant across different HLA imputation programs whenever the same alleles were interrogated. Our understanding is that *HLA-DPB1* was not included in the reference set used by HLA*IMP:01 and so could not be assessed in previous analyses [13, 21] using this software. Further associations with HLA-DQA1*03:*01* identified in our current analysis (Table 1, haplogroup 4) but not reported in previous analysis of these data [13] appear to be part of the previously identified HLA-DRB1*04:04/HLA-DQB1*03:*02* haplogroup.

**Table 1:** Comparison of highly significant marginal association results (P<0.000001) from 4 packages: HLA*IMP:02, HLA*IMP:03, HIBAG (calculated dosages and best guess) and SNP2HLA. HLA*IMP:01 results from Liu et al. (2012) are shown for comparison. Haplogroups were considered separate (i.e. independent) if predictors remained significant (P< 0.0001) when the top allele from any previously-identified haplogroup was included (pairwise) in the regression model.

Haplogroup	Gene	Allele	HLA*IMP:01 (dosages, results from Liu et al. 2012)		HLA*IMP:02 (dosages)		HLA*IMP:03 (best guess)		HIBAG 1.2 (best guess, provided probability > 0.8)		HIBAG 1.2 (dosages)		SNP2HLA	
			OR	P	OR	P	OR	P	OR	P	OR	P	OR	P
1	HLA-DQA1	04:01	3.07	5.90E-45	3.14	5.94E-49	3.05	1.16E-44	3.04	2.63E-42	3.14	3.48E-45	3.08	2.64E-45
	HLA-DQB1	04:02	3.04	1.91E-42	3.10	1.26E-45	3.04	2.35E-45	3.07	1.88E-44	3.08	1.37E-47	3.05	2.00E-45
	HLA-DRB1	08:01	3.18	1.14E-40	3.12	1.35E-45	3.16	2.18E-45	3.17	2.47E-37	3.26	1.25E-45	3.18	4.57E-45
	HLA-B	39:05^a^	5.48	4.81E-12	-	-	-	-	-	-	-	-	-	-
	HLA-B	39:06^a^	-	-	2.22	2.43E-09	2.28	7.72E-10	2.72	3.57E-10	2.78	1.60E-11	-	-
2	HLA-DQB1	06:02	0.64	2.32E-15	0.66	2.96E-16	0.66	8.96E-17	0.67	2.58E-15	0.66	2.14E-16	0.66	2.28E-16
	HLA-DRB1	15:01	0.65	2.78E-15	0.67	4.01E-16	0.67	3.89E-16	0.66	9.55E-15	0.67	4.57E-16	0.67	4.21E-16
	HLA-DQA1	01:02	0.69	4.19E-15	0.71	9.52E-16	0.72	6.06E-15	0.71	6.60E-15	0.71	3.26E-15	0.72	9.60E-15
	HLA-B	07:02	0.73	4.93E-10	0.73	4.14E-11	0.73	4.83E-11	0.72	1.08E-11	0.73	3.96E-11	0.73	3.62E-11
3	HLA-DQB1	03:01	0.70	6.48E-14	0.72	7.79E-15	0.72	4.92E-15	0.71	2.63E-15	0.70	1.93E-15	0.71	3.78E-15
	HLA-DRB1	11:01	0.33	2.14E-13	0.35	6.78E-19	0.46	1.60E-15	0.43	0.001	0.31	1.15E-18	0.41	8.57E-17
	HLA-DRB1	11:04	0.24	3.72E-09	0.14	1.38E-10	0.44	9.45E-06	0.44	0.011	0.14	5.00E-13	0.32	1.69E-07
	HLA-DRB1	11:03	-	-	0.31	0.076	0.65	0.202	-	-	0.002	2.15E-10	-	-
	HLA-DQA1	05:01^b^	0.75	4.76E-12	0.90	0.013	0.75	1.01E-13	0.98	0.654	0.98	0.5895	0.75	1.38E-13
	HLA-DQA1	05:05^b^	-	-	0.37	4.22E-26	-	-	0.49	1.31E-25	0.50	1.73E-25	-	-
4	HLA-DRB1	04:04	1.57	1.22E-09	1.61	8.59E-11	1.45	4.29E-09	1.45	4.09E-05	1.64	4.17E-10	1.54	1.25E-10
	HLA-DRB1	04:03	-	-	3.89	1.25E-07	1.32	0.183	1.23	0.626	2.92	1.21E-05	-	-
	HLA-DQB1	03:02	1.34	6.96E-09	1.34	7.83E-10	1.33	8.62E-10	1.33	2.11E-09	1.34	1.04E-09	1.28	1.45E-07
	HLA-DQA1	03:01	-	-	1.22	2.29E-06	1.14	4.14E-04	1.36	1.41E-09	1.37	1.03E-09	1.15	2.20E-04
5	HLA-DPB1	03:01	-	-	1.69	1.20E-31	1.53	3.68E-24	1.49	2.04E-07	1.80	1.60E-26	1.69	1.08E-27
	HLA-DPB1	06:01	-	-	-	-	1.71	1.51E-10	-	-	9.14	3.26E-26	1.97	6.30E-09
6	HLA-DPB1	04:01	-	-	0.74	2.04E-18	0.78	3.29E-16	0.81	4.62E-09	0.75	1.29E-18	0.76	2.23E-17
7	HLA-C	04:01	-	-	1.37	2.76E-10	1.37	3.15E-10	1.37	2.93E-10	1.37	4.01E-10	1.37	3.98E-10
8	HLA-DPB1	10:01	-	-	2.71	0.002	1.91	2.68E-11	2.19	4.11E-13	2.04	1.90E-12	1.93	3.09E-11
9	HLA-DPB1	17:01	-	-	2.43	0.005	2.40	1.03E-14	2.84	1.30E-17	2.47	5.55E-15	2.48	1.66E-15
10^c^	HLA-DPA	02:01	-	-	-	-	1.27	2.03E-09	-	-	-	-	1.27	2.08E-09

^a^ Note that HLA-B 39:05 and 39:06 are perhaps being used interchangeably: HLA*IMP:01 identified 39:05 whereas HLA*IMP:02, HLA*IMP:03 and HIBAG identify 39:06. Neither 39:05 or 39:06 are present in the SNP2HLA analysis.

^b^ Note that there are only two amino acids that differ between HLA-DQA1 05:01 and 05:05, one of which corresponds to our 5^th^ most significant result (position -13 of DQA1, see Table 2). This could perhaps explain the discrepancies between the results from the different programs for these alleles. We note that when HIBAG and HLA*IMP:02 identified either 05:01 or 05:05, the other programs (HLA*IMP:01, HLA*IMP:03 and SNP2HLA) identified exclusively 05:01

^c^ Note that HLA-DPA is only examined in HLA*IMP:03 and SNP2HLA

Our detected association at HLA-DPB1*03:*01* is consistent with results from HLA imputation in a medium-sized Italian PBC data set (676 cases and 1440 controls) in which Invernizzi et al. [22] used the Beagle software [23], in conjunction with the T1DGC HLA reference set, to demonstrate association of HLA-DPB1*03:*01* with disease [22]. Previous much smaller studies had generated somewhat contradictory results, with some showing [24] and others not showing [8] association between HLA-DPB1*03:*01* and PBC.

In addition to examining the effects of individual classical HLA alleles, we also used the HIBAG imputed dosages to perform multi degree-of-freedom (df) omnibus gene-based tests, examining the effects of all alleles (with frequency > 0.5%) at a gene simultaneously, although we note that the large (and differing) numbers of alleles at each HLA gene makes this procedure arguably less powerful and interpretable than the testing of individual alleles. All genes showed highly significant marginal association (S1 Table), with all genes except HLA-DQB1 retaining some level of support even conditional on the other genes (i.e. when all non-rare alleles at all other genes were included in the model).

For our primary analyses, we did not consider it necessary to include additional covariates such as gender or principal component scores (PCs) in the regression model to account for possible population stratification (see S1 Text); an investigation of the sensitivity of the results to inclusion or not of these covariates (S2 Table) suggested that the odds ratios (ORs) and P-values achieved were largely unaffected by the inclusion or not of the top 10 PCs (calculated from a pruned set of SNPs—with SNPs in the extended HLA region removed) and were only slightly altered by the additional inclusion of gender as a covariate. We also found that the marginal associations of the lead allele from each haplogroup shown in Table 1 were largely reproduced when modelled as part of a 9 variable model (with all lead alleles included simultaneously) (S3 Table).

### Association with amino acid substitutions

**Fig 1:**
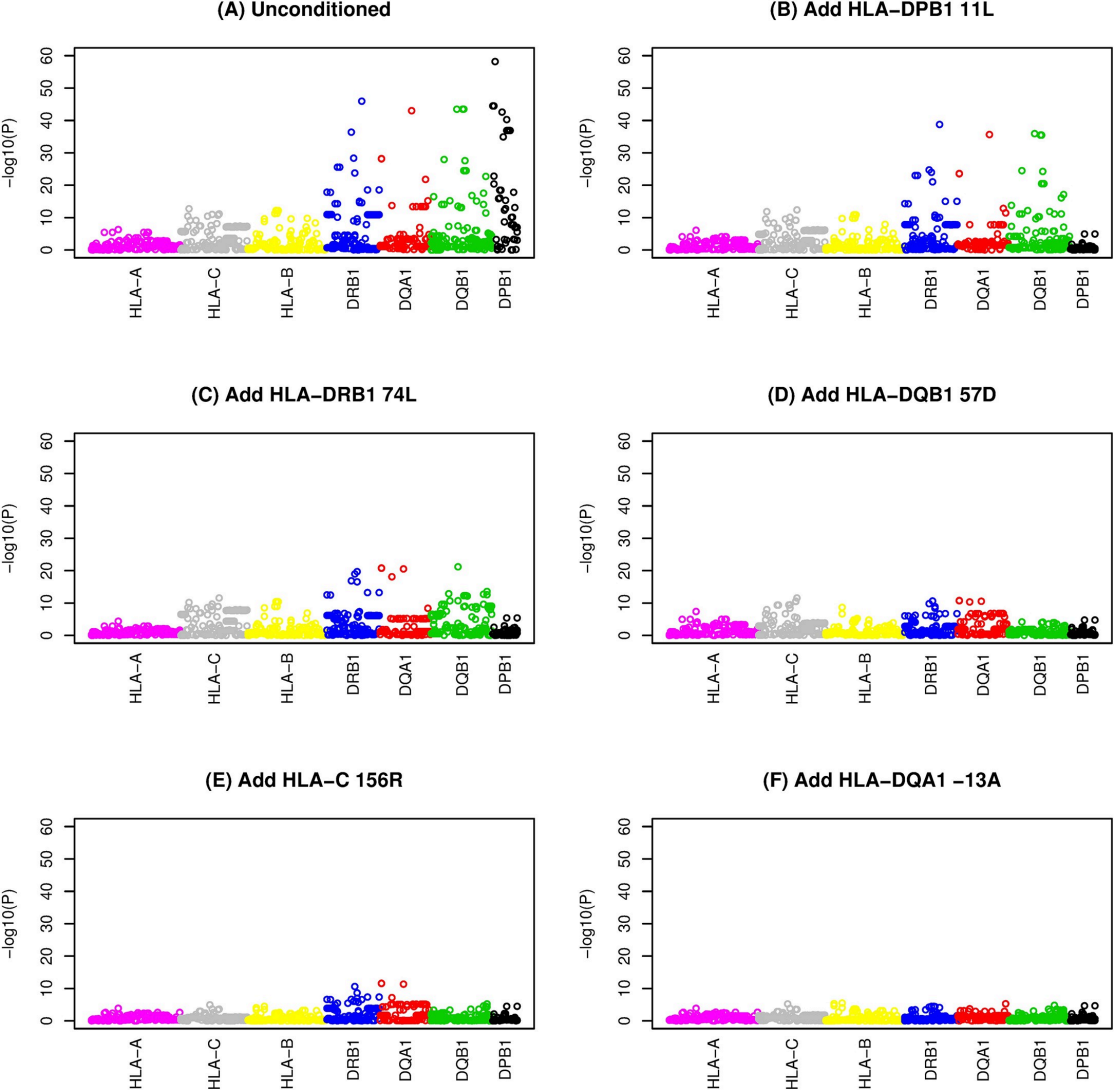
Stepwise logistic regression of amino acid residue dosages with up to five amino acid residue dosages included as covariates. The x axis denotes amino acid position. A) no conditioning, B) conditioned on HLA-DPB11L, C) conditioned on HLA-DPB11L and HLA-DRB74L, D) conditioned on HLA-DPB11L, HLA-DRB74L and HLA-DQB57D, E) conditioned on HLA-DPB11L, HLA-DRB74L, HLA-DQB57D and HLA-C155R and F) conditioned on HLA-DPB11L, HLA-DRB74L, HLA-DQB57D, HLA-C155R and HLA-DQA-13A.

Given that classical HLA alleles encode combinations of amino acid substitutions at specific positions, and given the *a priori* functional relevance of amino acid substitutions, we next focussed our attention on the variables encoding their effects. To determine whether the association seen between PBC and HLA SNPS and/or classical alleles could be explained by the amino acid substitutions encoded by the associated HLA alleles, we tested the imputed dosage of each amino acid residue at each amino acid position for association with PBC using logistic regression (Fig 1A, Table 2, S1 Spreadsheet). The marginal association results obtained using classical alleles imputed from HIBAG were seen to be highly concordant with those obtained directly from SNP2HLA; we focus here on the results from HIBAG which allows more complex subsequent modelling via regression and stepwise regression approaches (on account of outputting a posterior probability for each possible genotype, in contrast to the estimated dosage output by SNP2HLA, see Methods).

**Table 2:** Amino acid residues significantly associated (P<4.87E-05) with PBC in forward stepwise regression analysis. Shown are odds ratios (ORs) and P-values from stepwise and marginal logistic regression calculated using posterior probabilities from HIBAG, together with marginal associations from SNP2HLA. Classical alleles in bold are significantly associated with PBC in the current study, see Table 1.

Gene	BP position	Peptide position	Residue	Stepwise associations (HIBAG 1.2 dosage)		Marginal associations (HIBAG 1.2 dosage)		Marginal associations (SNP2HLA)		Classical HLA alleles within same gene carrying first listed residue observed in our study (imputed frequency in cases; imputed frequency in controls)
				Order of entry	P to enter	OR	P	OR	P	
HLA-DPB1	33156444	11	L/G	1	6.64E-59	1.765	6.64E-59	1.752	1.09E-58	**03:01 (0.14; 0.093)**, **06:01 (0.032; 0.021),** 09:01 (0.0086; 0.0072), **10:01 (0.032; 0.017),** 104:01 (0.017; 0.011), 11:01 (0.031; 0.026), 13:01 (0.022; 0.016), 14:01 (0.014; 0.013), **17:01 (0.025; 0.011)**
HLA-DRB1	32659927	74	L	2	1.73E-39	3.065	1.14E-46	2.828	5.65E-43	**08:01 (0.056; 0.019)**, 08:02 (0.0010; 0.00051), 08:03 (0.0045; 0.0017), 08:04 (0.0013; 0.00082), 08:10 (0.00075; 0.00032), 13:18 (0.0000085; 0.000018)
HLA-DQB1	32740667	57	D	3	6.42E-22	0.789	2.87E-14	0.787	9.69E-15	**03:01 (0.14; 0.18)**, 03:03 (0.044; 0.052)**, 04:02 (0.059; 0.020**), 06:01 (0.0051; 0.0037)**, 06:02 (0.095; 0.14)**, 06:03 (0.044; 0.053),06:14 (0.00030; 0.00030), 06:15 (0.00000; 0.00000)
HLA-C	31346910	156	R	4	2.70E-12	1.255	7.16E-12	1.250	1.20E-11	01:02 (0.042; 0.034)**, 04:01 (0.11; 0.084)**, 04:03 (0.000025; 0.000012), 04:09 (0.00030; 0.00019), 05:01 (0.13; 0.12), 08:02 (0.037; 0.039), 14:02 (0.011; 0.0075), 14:03 (0.00016; 0.00017), 18:01 (0.0000055; 0.000016)
HLA-DQA1	32713244	-13	A	5	2.48E-12	1.997	6.02E-29	-	-	01:01 (0.14; 0.12)**, 01:02 (0.14; 0.19)**, 01:03 (0.047; 0.053), 01:04 (0.029; 0.022), 01:05 (0.0046; 0.0053), 01:06 (0.00010; 0.00015), 02:01 (0.16; 0.15)**, 03:01 (0.13; 0.10)**, 03:02 (0.0082; 0.011), 03:03 (0.093; 0.094), **04:01 (0.057; 0.019)**, 04:02 (0.00079; 0.00030), 05:01 (0.14; 0.14), 05:03 (0.00030; 0.00024), 06:01 (0.0053; 0.0022)
HLA-B	31432581	45	T	6	3.13E-06	1.280	2.35E-10	1.278	1.65E-10	18:01 (0.043; 0.037), 18:02 (0.000041; 0.000035), 18:03 (0.00045; 0.00078), 18:11 (0.00023; 0.00017), 18:18 (0.000029; 0.000028), 35:01 (0.062; 0.050), 35:02 (0.0028; 0.0037), 35:03 (0.017; 0.011), 35:08 (0.0030; 0.0026), 35:17 (0.00029; 0.00034), 35:41 (0.00032; 0.00024), 35:55 (0.000020; 0.000016), 37:01 (0.017; 0.015), 44:06 (0.00026; 0.00018), 51:01 (0.046; 0.036), 51:05 (0.00022; 0.00024), 51:07 (0.00053; 0.00037), 51:08 (0.0010; 0.00068), 51:09 (0.00010; 0.00011), 52:01 (0.0054; 0.0044), 53:01 (0.0027; 0.0019), 58:01 (0.0047; 0.0051)
HLA-DQA1	32718440	207	V	7	4.38E-06	1.400	6.35E-16	1.386	1.23E-14	01:01 (0.14; 0.12), 01:03 (0.047; 0.053), 01:04 (0.029; 0.023), 01:05 (0.0046; 0.0053), 02:01 (0.16; 0.15), **03:01 (0.13; 0.10)**, 03:03 (0.093; 0.094), **04:01 (0.057; 0.020)**, 04:02 (0.00079; 0.00030), 05:01 (0.14; 0.14), 05:03 (0.00030; 0.00024), 05:05 (0.050; 0.095), 05:09 (0.00067; 0.00093), 06:01 (0.0053; 0.0022)
HLA-DPB1	33156663 or 33161618	84 or 215	V or T	8a or 8b	2.18E-05	1.713	0.000913	NA (OR = 0.550 for residue I)	NA (P = 4.74E-34 for residue I)	15:01 (0.011; 0.0068)
HLA-B	31432689	9	H	9	2.15E-05	1.181	1.07E-05	1.19	2.80E-06	18:01 (0.043; 0.037), 18:02 (0.000041; 0.000035), 18:03 (0.00045; 0.00078), 18:11 (0.00023; 0.00017), 18:18 (0.000029; 0.000028), 27:02 (0.0028; 0.0033), 27:03 (0.0000090; 0.000034), 27:05 (0.041; 0.040), 27:07 (0.00019; 0.00016), 27:09 (0.000045; 0.000097), 37:01 (0.017; 0.014), 40:01 (0.072; 0.056), 40:02 (0.0074; 0.010), 40:06 (0.00033; 0.00033), 40:27 (0.000030; 0.000045), 40:32 (0.00045; 0.00028), 41:01 (0.0024; 0.0021), 41:02 (0.0041; 0.0041), 42:02 (0.000077; 0.000046), 45:01 (0.0078; 0.0073), 49:01 (0.010; 0.012), 50:01 (0.014; 0.0089), 50:02 (0.00019; 0.00013), 73:01 (0.00019; 0.00013)

The strongest association (*P* = 6.64x10^-59^) was seen with residue L (the substitution leucine for glycine) at position 11 of HLA-DPβ1; an equivalent association was seen with residue G (reflecting the fact that at this position there are only two possible substitutions, and so the test of L versus G is equivalent to the test of G versus L). Once the variable encoding this effect had been included as a covariate in the regression model, the next most associated amino acid (*P* = 1.73x10^-39^) was residue L at position 74 of HLA-DRβ1. HLA-DRβ1 74L was previously identified as significantly associated with PBC by Donaldson et al. [1] and Invernizzi et al. [22], as well as by an earlier small Japanese study (53 PBC patients and 60 controls) [5]. This substitution occurs both on classical alleles HLA-DRB1*08:*01* (which is strongly associated with PBC in Europeans) and on HLA-DRB1*08:*03* (which is known to be associated with PBC in Japanese/Chinese populations [4, 5, 25] and thus offers a potential explanation for the HLA-DRB1*08 associations seen in these different populations. Our identification of amino acids in HLA-DPβ1 and HLA-DRβ1 as the top contributors to HLA-induced PBC risk is consistent with the results of Invernizzi et al. [22] who found in their (much smaller) Italian data set that conditioning on residue L at position 11 of HLA-DPβ1 largely removed the signal at *HLA-DPB1*, and who noted that, considered together, HLA-DRB1*08 and HLA-DPB1*03:*01* accounted for the majority of the signal in the HLA region.

### Stepwise regression analysis of amino acid substitutions

**Fig 2:**
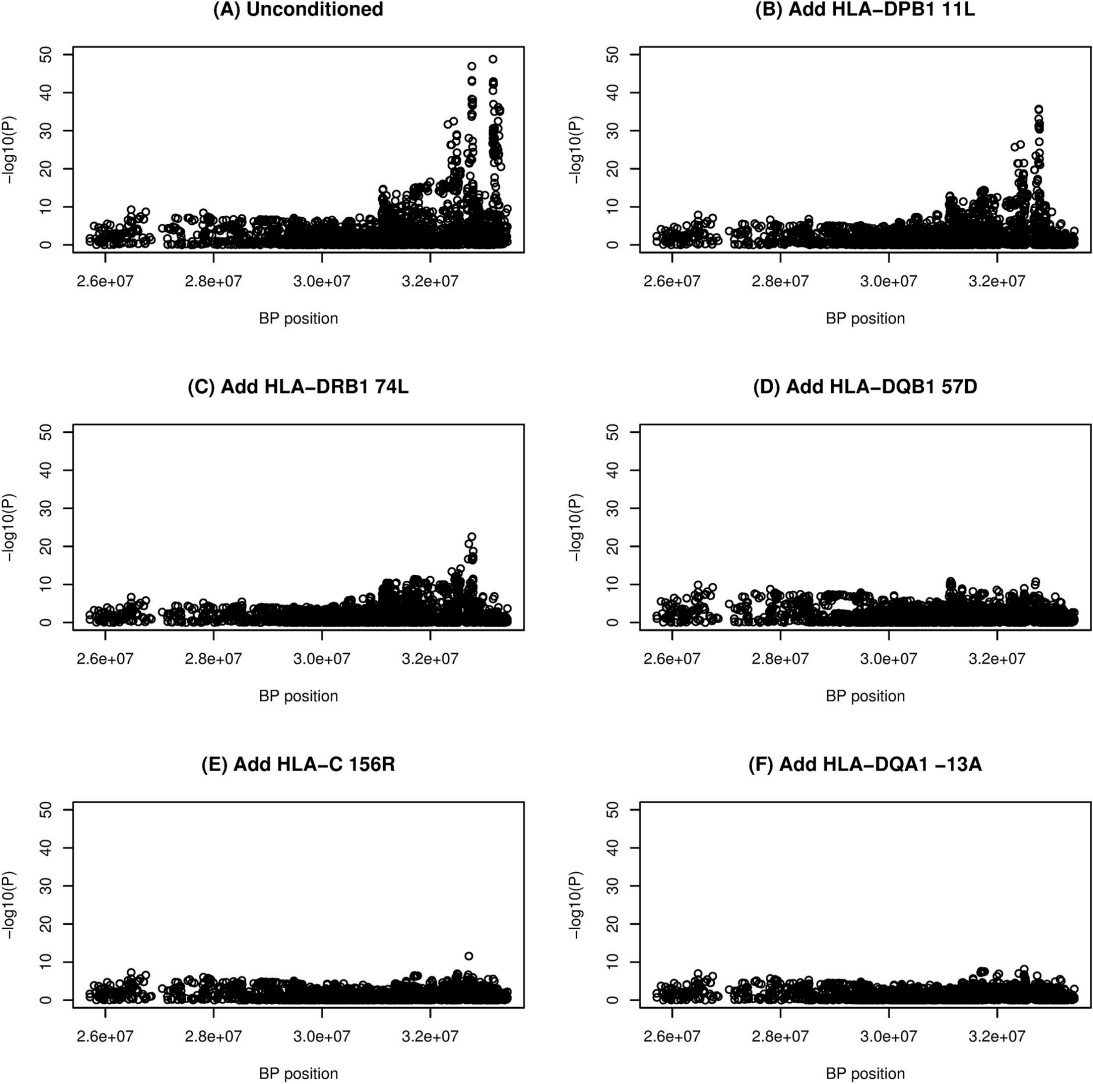
Stepwise logistic regression of individual SNPs with up to five amino acid residue dosages included as covariates. The x axis denotes base pair position on chromosome 6. A) All SNPs, no conditioning, B) conditioned on HLA-DPB11L, C) conditioned on HLA-DPB11L and HLA-DRB74L, D) conditioned on HLA-DPB11L, HLA-DRB74L and HLA-DQB57D, E) conditioned on HLA-DPB11L, HLA-DRB74L, HLA-DQB57D and HLA-C155R and F) conditioned on HLA-DPB11L, HLA-DRB74L, HLA-DQB57D, HLA-C155R and HLA-DQA-13A.

**Fig 3:**
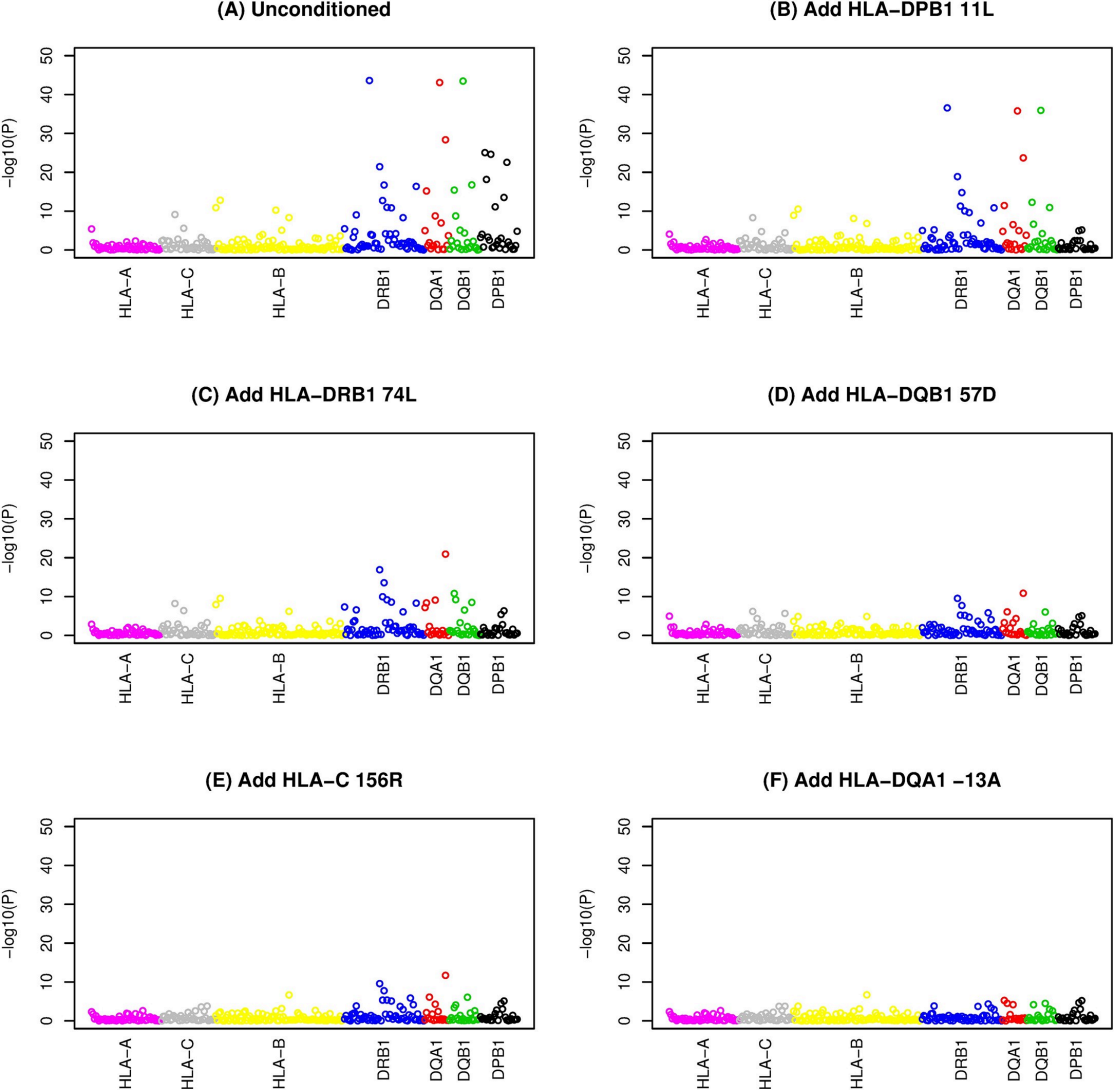
Stepwise logistic regression of classical HLA alleles with up to five amino acid residue dosages included as covariates. The ordering of the alleles within a gene along the x axis is alphabetical by allele name. A) no conditioning, B) conditioned on HLA-DPB11L, C) conditioned on HLA-DPB11L and HLA-DRB74L, D) conditioned on HLA-DPB11L, HLA-DRB74L and HLA-DQB57D, E) conditioned on HLA-DPB11L, HLA-DRB74L, HLA-DQB57D and HLA-C155R and F) conditioned on HLA-DPB11L, HLA-DRB74L, HLA-DQB57D, HLA-C155R and HLA-DQA-13A.

Using a stepwise regression approach similar to that used in previous studies [26–28], we continued adding amino acid residues into the regression model in a stepwise fashion to account for their effects [29] until none reached significance level < *P* = 4.87x10^-5^ (representing a Bonferroni-corrected threshold of 0.05, allowing for 1028 amino acids tested); this resulted in a final model that included nine amino acids (Table 2). Use of a more stringent stopping threshold of *P* = 1.0x10^-8^ resulted in a final model that included five amino acids in five separate genes (Table 2, Fig 1A–1F, S1 Spreadsheet). None of the top nine or top five amino acids dropped out of the model (all *P*>4.87x10^-5^) when allowing a backward stepwise step (S4 Table). Stepwise inclusion of the top five amino acids in association analyses carried out with respect to individual SNPs (Fig 2, S2 Spreadsheet) or classical HLA alleles (Fig 3, S3 Spreadsheet) indicated that these five amino acids could account for the majority of the HLA association seen at the level of SNPs or classical alleles; once these five amino acids had been included, the minimum significance levels achieved were *P* = 7.05x10^-9^ for SNPs and *P* = 1.98x10^-7^ for classical alleles. Thus, although some residual association remains, inclusion of the top five amino acids is sufficient to remove the strongest disease associations observed. Interestingly, in spite of the strong linkage disequilibrium (LD) across the HLA region (resulting in haplogroups spanning multiple genes, see Table 1), visual inspection of Fig 3 suggests that each of the five implicated amino acid residues accounts primarily for the disease association observed with classical alleles of its own gene, although HLA-DRβ1 74L does partly account for association seen at *HLA-DQA1* and *HLA-DQB1*, and HLA-DQβ1 57D in turn partly accounts for association seen at *HLA-DQA1* and *HLA-DRB1*, probably due to the long-range correlations (due to extensive LD) between alleles (and thus between amino acid substitutions and classical alleles) at different genes.

We investigated the sensitivity of our results to the inclusion of the top 10 principal component scores (calculated from a pruned set of SNPs—with SNPs in the extended HLA region removed) in order to account for possible population stratification, and also to the inclusion of gender, as covariates in the regression equation, but found (as expected from theoretical arguments, see Methods), that this had little impact on the results obtained, either with respect to the main effects of amino acids (S1 Fig, S4 Spreadsheet) or with respect to the stepwise entry of predictors. With principal component scores included, exactly the same top five amino acids entered the model, while, with principal component scores and gender included, four out of the top five identified amino acids (all still with *P*<1.0x10^-8^) remained the same, with HLA-DQβ1 87F entering the model in preference to the marginally less significant HLA-DQβ1 57D, and with the order of entry slightly altered as follows: (1) HLA-DPβ1 11L/G (*P* = 5.70x10^-53^), (2) HLA-DRβ1 74L (*P* = 4.67x10^-33^), (3) HLA-DQα1 -13A (*P* = 2.34x10^-20^), (4) HLA-DQβ1 87F (*P* = 1.18x10^-13^), and (5) HLA-C 156R (*P* = 2.23x10^-10^).

We additionally investigated the consistency/stability of the predictors identified by the stepwise selection procedure using a resampling approach (see Methods), and found the approach to be highly stable in terms of the top amino acid predictors identified. In 1000 bootstrap replicates (each containing 2/3 of our PBC cases and 2/3 of our controls, see Methods) the top amino acid HLA-DPβ1 11L/G entered as the most significant predictor in 86.7% of replicates (and entered as second in the remaining 13.3% of replicates). The second amino acid to enter was HLA-DRβ1 74L in 83.1% of replicates. The third amino acid to enter was HLA-DQβ1 57D in 51% of replicates (the closest competitor was HLA-DRβ1 67L which entered third in 25.8% of replicates). The fourth amino acid to enter was HLA-C 156R in 33.7% of replicates (the closest competitor was HLA-C 152A which entered fourth in only 10.3% of replicates). The fifth amino acid to enter was HLA-DQα1 -13A in 27.9% of replicates (the closest competitor was HLA-DRβ1 58A which entered fifth in only 10.5% of replicates).

### No association with KIR genes

The association seen between PBC and HLA-C 156R is intriguing, as HLA-C is known to be not very potent in antigen presentation. Given that HLA-C has a significant role in interaction with killer-cell immunoglobulin-like receptors (KIRs), this raises the question of whether the HLA-C association is related to T-cell interaction, or is rather about presentation to KIRs. We therefore used the software package KIR*IMP [30] to examine the association between PBC and genes on chromosome 19q13.4 that encode for KIRs. In contrast to the detected HLA associations, however, analysis of PBC association with imputed KIR haplotypes and copy number variation detected no significant associations (minimum observed P value = 0.07) between PBC and KIR variation. We therefore chose not to focus any further attention on the KIR gene region at this current time.

### Stepwise regression analysis of classical alleles, SNPs and amino acid substitutions

We continued our investigation by examining the association between PBC and amino acid residues, SNPs or classical HLA alleles simultaneously, by allowing either amino acid residues and/or SNPs and/or classical HLA alleles to enter the stepwise regression model at each step. At each of steps 1–2, an amino acid residue (HLA-DPβ1 11L at step 1 and HLA-DRβ1 74L at step 2) entered the model preferentially in comparison to a SNP or a classical HLA allele. This ability of amino acid substitutions to explain the association of the MHC to PBC in a more parsimonious way than is achieved by classical HLA alleles contrasts with results previously found using stepwise regression in inflammatory bowel disease (IBD) [27], where classical HLA alleles (specifically HLA-DRB1*01:*03*) entered the model first and better explained the association than models based on amino acid substitutions, leading the investigators in that study to focus their subsequent efforts on an *HLA-DRB1* centric model. Our results here are more akin to those found using stepwise regression in rheumatoid arthritis [28], where amino acid substitutions entered the model first and were found to provide a better fit, and a more parsimonious explanation for the observed association, than models based on either two- or four- digit classical alleles.

Continuing the stepwise regression procedure, our results at subsequent steps (S1 Text) illustrated the difficulty of disentangling “causal” from “hitchhiking” effects amongst highly correlated variables such as the amino acid residues, classical alleles and SNPs considered here—although it is noteworthy that in each of steps 1–4 an amino acid always entered the model in preference to a classical allele. In most cases, the difference in model fit between including the top SNP and the top amino acid or classical allele was relatively small. Given the *a priori* potential functional role of amino acid substitutions, we found it most natural to focus primarily on variables directly encoding these effects. The fact that, in some instances, inclusion of a SNP provided a slightly better model fit could indicate that the SNP itself is having a functional role (perhaps through a mechanism such as modulation of gene expression) but, equally, could arise from the phenomenon whereby a SNP tags the combined effects of several functional amino acids. In terms of accounting for the overall association in the region, we found the model that included the top 5 amino acids (S2 Fig, left hand panels) performed similarly to the model that included the top 5 variables of any type (S2 Fig, right hand panels, S5 Spreadsheet, S6 Spreadsheet, S7 Spreadsheet).

**Fig 4:**
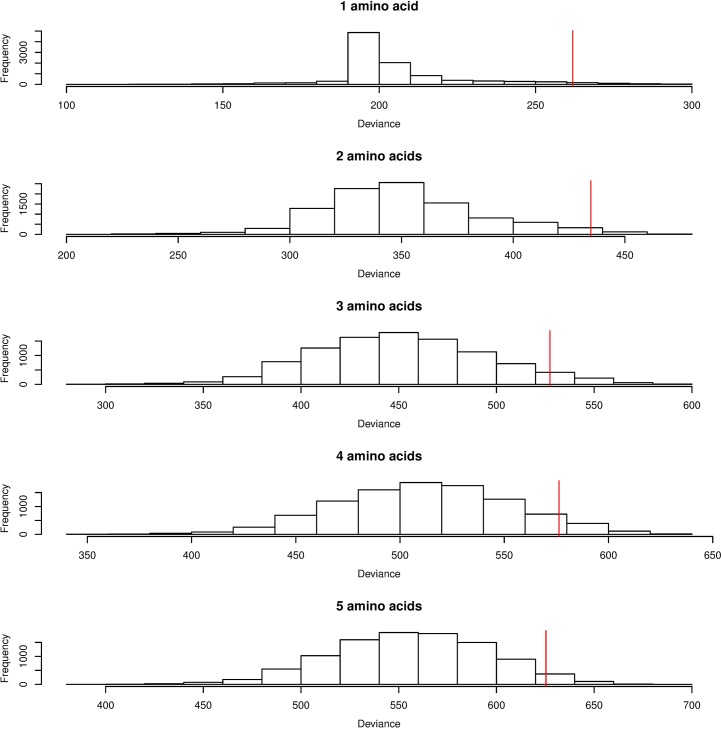
Empirical distribution of deviance in 10,000 permutations of amino acid sequences across classical HLA alleles. The vertical red lines indicate the deviances (261.89, 434.78, 527.37, 576.27, 625.34) explained by the top 1–5 amino acids in the actual data, which generate empirical P-values (0.0330, 0.0195, 0.0515, 0.0603, 0.0333) respectively.

To explore further the degree to which amino acid substitutions could account for the effects of classical alleles, and to investigate whether such results could occur by chance by tagging classical alleles of differential risk, we used the permutation approach employed in IBD [27]. Specifically, we repeatedly reassigned (permuted) the amino acid sequence assigned to each of the classical HLA alleles, creating a null hypothesis distribution whereby the relationship between classical HLA alleles and disease was retained, but the relationship between amino acid substitutions and classical alleles was permuted. The results (Fig 4, S8 Spreadsheet) indicated that the model deviance accounted for by the top 1–5 amino acids generally fell in the tail of the empirical null distribution, suggesting that the observed amino acid associations were unlikely to have arisen through chance tagging of classical HLA alleles.

### Alternative amino acid substitution explanations

To investigate whether alternative amino acid substitutions could provide an equally good explanation for the top amino acid associations, we examined the correlations between our top five residues and other amino acid residues (S5 Table). Substitutions L and G at position 11 of HLA-DPβ1 were perfectly correlated as previously noted; no other amino acid substitution reached r^2^ > 0.8 with this substitution. Similarly substitutions A and T at position -13 of HLA-DQα1 were almost perfectly correlated; no other substitution reached r^2^ > 0.8 with this substitution. Five alternative residues showed r^2^ >0.8 with HLA-DRβ1 74L and thus might be considered plausible alternative causal explanations for the association seen at this residue. No alternative residues reached r^2^ > 0.8 with the amino acid residues implicated at HLA-DQβ1 57D and HLA-C 156R, suggesting that these effects are unlikely to be attributable to alternative substitutions.

Similar to previous studies conducted in IBD [27], we additionally fitted multi-df omnibus models that included predictor variables encoding the effects of *all* amino acid substitutions at a position simultaneously (the maximum number of such amino acid variants at a position was 8). This analysis strategy investigates the *combined* effects seen at a particular position of the amino acid sequence, rather than the effects of individual specific amino acid residues. The results (S1 Text, S6 Table, S3 Fig, S4 Fig, S5 Fig, S9 Spreadsheet, S10 Spreadsheet, S11 Spreadsheet, S12 Spreadsheet) showed reasonable (albeit not perfect) concordance with the results seen when considering individual amino acid substitutions, while incurring the expense of a larger number of df and an arguably less interpretable model. Given that the five individual amino acid residues previously identified do almost as well at accounting for the association as do the multi-df models, overall we tend to prefer the five amino acid model identified through stepwise regression as representing the most parsimonious solution.

### Exhaustive and stochastic searches for best combinations of amino acid variables

Similar to previous studies conducted in rheumatoid arthritis [28], we also performed an exhaustive search for all pairs and all triplets of amino acid residues and amino acid positions, in order to determine the best pairwise or three-way combination associated with PBC. The best pairwise combination of amino acids was HLA-DPβ1 11L and HLA-DRβ1 74L, which corresponds to the top two residues identified through stepwise analysis (Table 2). The best (multi-df) pairwise combination of amino acid positions was position 11 of HLA-DPβ1 and position 27 of HLA-DQβ1, again the same as the top two positions identified through stepwise analysis (S6 Table). The best three-way combination of amino acids was HLA-DPβ1 11L, HLA-DQα1 -13A and HLA-DQα1 53G, which includes the first and 5^th^ amino acids identified through stepwise analysis; this combination was only very marginally better (AIC 12311.42) than the combination DPβ1 11L, HLA-DRβ1 74L and HLA-DQβ1 57D (AIC 12311.51) which corresponds to the top three amino acids identified through stepwise analysis. The best (multi-df) three-way combination of amino acid positions was position 11 of HLA-DPβ1, and positions 58 and 13 of HLA-DRβ1, corresponding to the first, 3^rd^ and 7^th^ positions identified through stepwise analysis (S6 Table).

To move beyond three-way combinations of predictors in an exhaustive search is computationally challenging. We therefore used the FINEMAP [31] and GUESSFM [32] programs which implement (slightly different) Bayesian stochastic search algorithms for selecting important predictors within a densely genotyped candidate region. Preliminary analyses with FINEMAP generated many equivalently-fitting models; we circumvented this issue by filtering out highly correlated amino acid variables, retaining an index set of 396 amino acids with pairwise correlation values less than 0.98 for analysis. The equivalent strategy in GUESSFM was achieved through setting the user-defined input parameter “tag.r2” (the r^2^ threshold for grouping predictors together into LD groups) as 0.9604 (= 0.98^2^); this resulted in 354 tag groups once monomorphic amino acid positions had been discarded. Following model fitting, the “expand.tags” function within GUESSFM package was then used to expand the set of models considered by GUESSFM to consider all predictors (rather than using a single “tag” amino acid as a surrogate for the other amino acids in its LD group) and the “snp.picker” function used to pick out the resulting amino acids that had the highest posterior probability of inclusion.

The top models and amino acids identified by FINEMAP and GUESSFM respectively are shown in S7 Table and S8 Table. We found the results from FINEMAP and GUESSFM to be somewhat sensitive to the choice of user-defined input parameters, particularly the “nexp” parameter (the expected number of causal variants) in GUESSFM and the maximum number of causal variants in FINEMAP. The results from FINEMAP (S7 Table) were relatively concordant with those from stepwise regression, strongly implicating four out of the top five amino acids from stepwise regression (HLA-DPβ1 11L/G, HLA-DRβ1 74L, HLA-DQβ1 57D and HLA-C 156R), but also providing some support for additional predictors HLA-DQβ1 -4V, HLA-DQβ1 71T, HLA-DQα1 175E and HLA-B 45T (or their correlates). The results from GUESSFM (S8 Table) were more variable and, in general, GUESSFM generated final models that involved a relatively large number of predictors compared to stepwise regression. However, three out of the top five amino acids from stepwise regression (HLA-DPβ1 11L/G, HLA-DRβ1 74L and HLA-C 156R) retained strong levels of support, which was maintained following application of GUESSFM’s snp.picker algorithm (S9 Table).

Given the strong LD in the HLA region, it is perhaps not surprising that GUESSFM ended up preferring models with large numbers of predictors which can better capture subtle haplotype effects. Our comparison between these different analysis approaches again illustrates the difficulty of statistically identifying true *causal* variants (as opposed to good *markers* of causal variants) in regions of high LD such as the HLA region. S6 Fig, S7 Fig, S8 Fig, S9 Fig, S10 Fig and S11 Fig (see also S13 Spreadsheet, S14 Spreadsheet, S15 Spreadsheet) illustrate the degree to which the top predictors implicated by FINEMAP and GUESSFM can account for the amino acid, SNP and classical allele associations observed in the region. Although FINEMAP and GUESSFM perform well when larger numbers of predictors are included, they did not generally outperform stepwise regression when limited to 5 predictors. The fact that the five amino acid model identified through stepwise regression performs well at explaining the observed SNP and classical allele association again motivates the five amino acid model as representing arguably the most parsimonious solution.

### Dominant, recessive, genotypic and interaction models

Given that non-multiplicative effects at HLA have been observed in other autoimmune diseases [33], for each of the classical HLA alleles (Table 1) and amino acid substitutions (Table 2) identified using the 1df multiplicative allelic model, we additionally explored models that allowed the effects to operate via dominant, recessive, genotypic or interaction effects. However, we found little compelling support for such models from the data (S2 Text, S10 Table, S11 Table). In most cases there was little difference in fit between the multiplicative and dominant models, suggesting insufficient data (in particular insufficient observations with two copies of the allele in question) as to be able to distinguish between these two scenarios. We additionally performed pairwise interaction analysis to investigate whether particular combinations of classical HLA alleles or amino acid residues led to increased or reduced risks (over and above their individual multiplicative effects) (S2 Text) but found no evidence of any significant interactions, once Bonferroni correction had been made for the number of tests performed.

### 3D protein structure modelling and calculation of electrostatic potentials

To explore the potential functional consequences of changes at the key PBC-associated amino acid residues identified, we followed an approach previously used in primary sclerosing cholangitis [34]. HLA alleles carrying and not carrying the associated residues were three-dimensionally modelled using the program MODELLER 9.14 [35], electrostatic potentials around the resulting 3D structures were calculated using DelPhi 6v2 [36], and the surface of the modelled molecules were coloured according to charge using Chimera [37]. We focussed on the top three amino acid residues (HLA-DPβ1 11L, HLA-DRβ1 74L, HLA-DQβ1 57D) identified through stepwise regression (Table 2), all of which showed strong marginal association with PBC, and we modelled HLA molecules corresponding to alleles showing significant marginal association with PBC (Table 1) that either carried or did not carry the associated amino acid residue. We also investigated the electrostatic potential of residues 56, 70 and 71 in HLA-DQβ1 on account of their strong correlation with the second top amino acid HLA-DRβ1 74L (S5 Table). In relation to the top two residues, it has previously been suggested that polymorphism at position 11 of HLA-DPβ1 has the potential to influence properties of binding pocket P9, while polymorphism at position 74 of HLA-DRβ1 may influence properties of binding pocket P4 [38]. HLA-DQβ1 57D, the critical residue that enters third in the stepwise regression procedure, is known to be associated with protection from type 1 diabetes [39]. Its carboxylate group forms a salt bridge with a conserved arginine at position 76 of HLA-DQα1 that stabilises the heterodimer and may affect peptide binding [38, 40].

**Fig 5:**
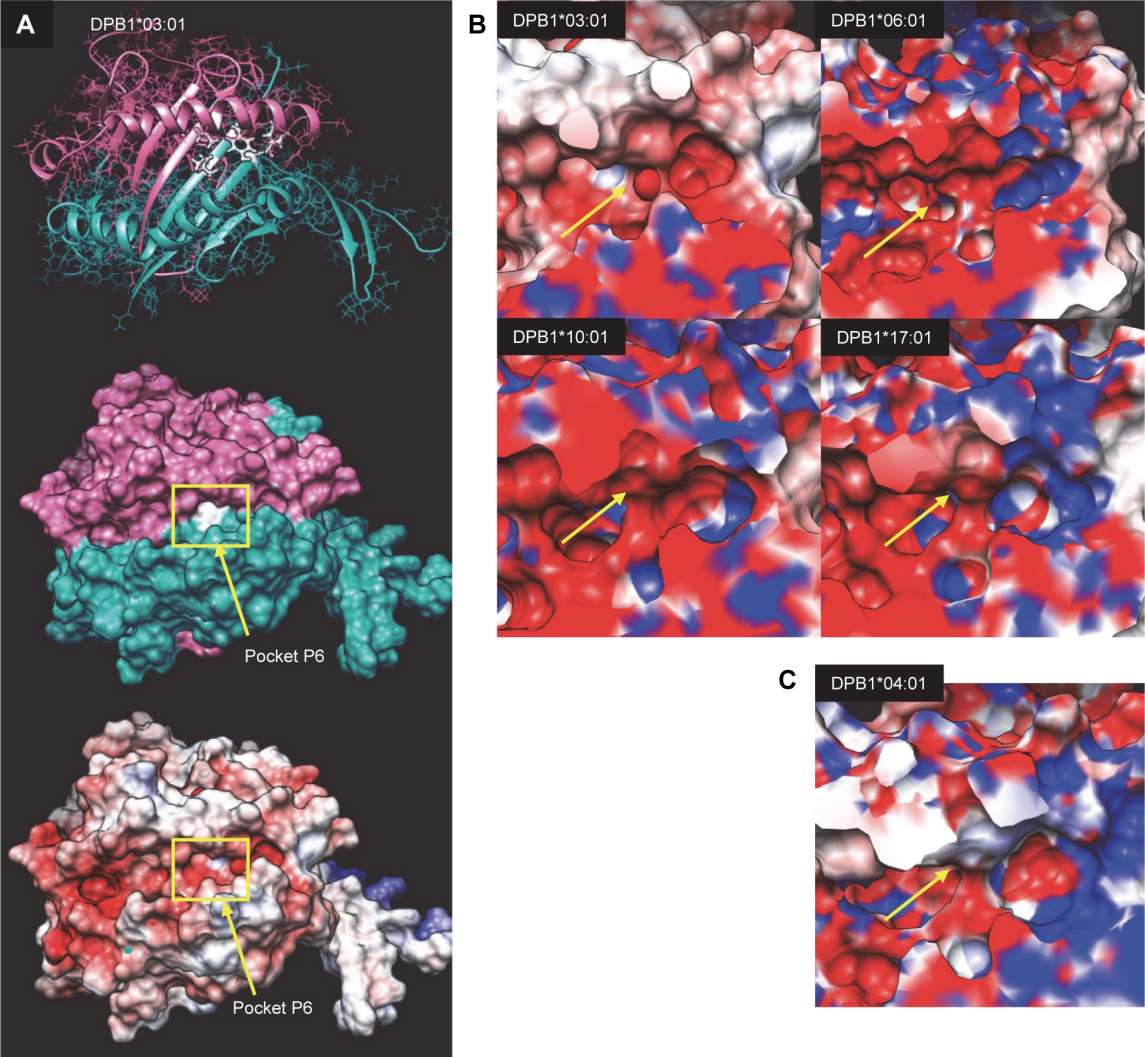
Structure and molecular surface electrostatic potential of pocket P6 in HLA-DP molecules. A) The structure and electrostatic potential of HLA-DPB1*03:01. The area within the frame is depicted in expanded form in B and C, and indicates the position of pocket P6 (arrows). All structures were superimposed on HLA-DPB1*03:01 and therefore show the same view. HLA-DPB alleles associated with an increased risk of PBC (03:01, 06:01, 10:01, 17:01) are shown in panel B whereas those associated with a protective effect (04:01) are shown in panel C. Negatively charged potentials (less than 5 kT/e) are coloured red, positively charged (greater than 5 kT/e) blue, and neutral potentials (0 kT/e) are coloured white. Linear interpolation was used to produce the colour for surface potentials between these values.

Results for HLA-DPβ1 11L (Fig 5) showed a remarkable correlation between the electrostatic potential of pocket P6 in HLA-DP molecules and the HLA-DPB1 alleles/amino acid substitutions conferring PBC susceptibility or protection. The PBC-associated HLA alleles (Fig 5B, top right) all contain an L at position 11 of the amino acid sequence, and show negative potentials, while the protective allele (Fig 5C, bottom right) contains a G at position 11, and shows neutral or slightly positive. Results for the other modelled alleles (S12 Fig, S13 Fig, S14 Fig) were less compelling in terms of demonstrating clear-cut correlations between electrostatic potentials and alleles/amino acid substitutions conferring PBC susceptibility or protection, suggesting that the mechanisms underlying these detected associations may be more complex than can be accounted for by simple amino acid substitution.

## Discussion

In this, the largest such study carried out to date in PBC, we present results from an investigation of the association between classical HLA alleles, the amino acids they encode, and PBC, through an analysis of pre-existing immunochip data using a variety of state-of-the-art HLA imputation software packages. Previous HLA imputation-based studies in PBC [22] have sought to determine whether the amino acid associations observed with PBC could be explained by classical HLA alleles; here we took the view, in common with earlier studies of PBC [1, 8] and of other diseases [8, 28, 34, 39, 41] that, given the functional relevance of amino acid substitutions, a more natural question is whether the association with classical alleles (and with SNPs in the HLA region) can be explained by the amino acid substitutions themselves.

We found that the majority of the strong association between PBC and SNPS in the HLA region and/or classical HLA alleles could indeed be explained by variation at five separate amino acid substitutions. These included two substitutions (HLA-DPβ1 11L/G and HLA-DRβ1 74L) that were previously implicated (at much lower levels of significance) by smaller earlier studies, and three substitutions that represent (to our knowledge) novel findings; once these five effects had been accounted for, there remained some residual association, but this was nowhere near as strong as the genome-wide levels of significance observed in marginal analysis. Given the *a priori* functional relevance of amino acid substitutions, we thus considered these amino acids (or other residues highly correlated with them) as good candidates for being causal; we note that this viewpoint—which places a strong prior on amino acids accounting for association at classical alleles being a more convincing explanation than classical alleles accounting for association of amino acids (given the fact that classical alleles can effectively be considered as specific combinations of amino acid substitutions)—is, to some extent, borne out by that fact that, in steps 1–4 of our stepwise analysis, an amino acid always entered the model in preference to a classical allele.

In our primary analyses, we focussed on examining the effects of individual amino acid substitutions and classical HLA alleles, reserving for secondary consideration omnibus tests that encode the effects of all amino acid substitutions at a position, or all alleles at a gene, simultaneously. This strategy differs from that used in previous studies of IBD [27] and RA [28], in which the investigators focussed first on omnibus tests, reserving tests of individual amino acid substitutions and classical HLA alleles for secondary consideration. Which analysis strategy is to be preferred is perhaps debatable. We considered the individual testing strategy to be *a priori* the more appealing and interpretable of the two approaches, on account of having fewer df (one df per residue/allele), which provides higher power than testing all residues/alleles at a position/gene simultaneously. For example, if only one residue out of 8 possible residues at a particular position was actually associated with the disease, then this would be clearly visible when testing individual amino acid substitutions, but the effect might well be drowned out when constructing a combined test of all 8 substitutions (one of which is associated, and 7 of which are not). A further reason for preferring the test of individual residues/alleles is the fact that individual residues/alleles may themselves have specific functional effects, separate from the effects of any other residues/alleles at the same position/gene. In application to our PBC data set, we did not find the multi-df omnibus approach to add substantial significance compared to testing individual predictors, perhaps justifying retrospectively our choice of analysis strategy.

We took forward the implicated amino acid residues from our analyses for three dimensional predictive modelling and calculation of electrostatic potentials, following an approach that was previously used in primary sclerosing cholangitis [34] but which has not, to our knowledge, been previously used in PBC. This analysis demonstrated a correlation between the electrostatic potential of pocket P6 in HLA-DP molecules and the HLA-DPB1 alleles/amino acid substitutions conferring PBC susceptibility/protection, highlighting a potential mechanistic explanation for the observed association that warrants further investigation.

A previous study in IBD [27] addressed this question of electrostatic potentials in a slightly different way, motivated by their finding that classical HLA alleles (specifically HLA-DRB1*01:*03*) better explained the IBD association than did models based on amino acid substitutions, leading the investigators to focus their subsequent efforts on an *HLA-DRB1* centric model. In that study, Goyette et al. [27] followed three dimensional predictive modelling with an analysis of the electrostatic properties around the seven peptide residues (at peptide positions 1, 2, 3, 4, 5, 7 and 9) that are known to make contact within the binding grove, and then clustered electrostatically similar HLA-DR molecules together. (A similar analysis was also carried out focussing only on electrostatic properties in the region affected by amino acid positions 67, 70 and 71, which had shown significant association with IBD). This analysis identified four clusters of HLA-DR molecules sharing similar electrostatic properties with respect to the seven peptide regions (or two clusters sharing similar electrostatic properties with respect to the region encompassing amino acid positions 67, 70 and 71). The resulting clustering of *HLA-DRB1* alleles (encoding these molecules) showed that alleles associated with increased risk of IBD generally fell into different clusters than alleles associated with decreased risk of IBD, suggesting that the HLA-DR molecules associated with increased risk of IBD exhibited structural and electrostatic properties within the peptide-binding grove that were largely distinct from those associated with decreased risk.

Given the strong associations of *HLA-DRB1* alleles with PBC risk (Table 1), and the fact that our second most associated amino acid (*P* = 1.73x10^-39^) is residue L at position 74 of HLA-DRβ1, we investigated how the *HLA-DRB1* alleles associated with either increased or decreased PBC risk in our study mapped onto the four clusters of electrostatically similar HLA-DR molecules that had been identified by Goyette et al. In contrast to the results seen in IBD, we did not find a consistent pattern of alleles associated with increased risk of PBC falling into different clusters than alleles associated with decreased risk of PBC, suggesting that the mechanisms underlying the *HLA-DRB1* associations with PBC may be more specific than is captured by this analysis.

Although the amino acid substitutions highlighted here represent the most compelling disease-causing factors implicated by our study, we note that high LD in the region and the ability of alternative, more complex, models (such as those examined in our multi-df, FINEMAP and GUESSFM analyses) to account for the disease associations observed means that we cannot definitively rule out the contribution of other factors whose effects are statistically intertwined with the substitutions we have identified; follow-up functional studies will be required to further investigate this question. It has previously been demonstrated that HLA-DRB1*08:*01* (DR0801), but not HLA-DRB1*11:*01* (DR1101), can bind functional epitopes derived from the dominant autoantigen pyruvate dehydrogenase complex-E2 (PDC-E2) [42]. We hypothesise that epitope analysis (using resources such as the Immune Epitope Database and Analysis Resource) may suggest that the identified significant DQ residues are likely to be equally significant immunologically, with risk, but not protective alleles being permissive for binding of binding of epitopes derived from the immuno-dominant inner lipoyl binding domain of PDC-E2.

The association seen between PBC and HLA-C 156R, implicated by all analysis methods, is intriguing, as HLA-C is known to be not very potent in antigen presentation. However, HLA-C does have a significant role in interaction with killer-cell immunoglobulin-like receptors (KIRs), and this therefore raises the question of whether the HLA-C association is related to T-cell interaction, or is rather about presentation to KIRs. The fact that, in our data set, we observed no association between PBC and imputed KIR haplotypes and copy number variation makes this explanation seem less plausible, but it remains an interesting topic for future investigation.

## Methods

### Ethical approval

This study was approved by the Research and Development Departments of all National Health Service (NHS) Trusts participating in this study and by the Oxford Research Ethics Committee C (Oxford REC C reference 07/H0606/96).

### Study samples and genotyping

The cases, controls and genotype data used here have been described previously [11, 13]. In brief, a total of 2981 PBC cases were contributed by the UK PBC Consortium, a consortium operating within 142 NHS trusts, including all UK liver transplant centres. All cases were of self-declared British or Irish ancestry, over 18 years old with probable or certain PBC. 8,970 controls of self-declared British or Irish ancestry were provided by the 1958 British Birth Cohort and the National Blood Service. Samples were genotyped on an Illumina iSelect HD custom genotyping array at either the Wellcome Trust Sanger Institute (2981 cases and 4537 controls) or the Center for Public Health Genomics at the University of Virginia (4433 controls). Following sample and SNP quality control, we retained 2861 cases and 8514 controls that passed previously-derived quality control checks [13], genotyped at 143,006 SNPs, of which 7848 fell within the extended MHC region [43] on chromosome 6 (ranging from 25,650,000 to 33,426,000 base pairs, Build36). One SNP within the region (rs2394173) showing apparent association with disease status was subsequently excluded from analysis following visual inspection of its cluster plots.

### HLA imputation

A variety of different software packages have been developed for the imputation of classical HLA alleles (and, in some cases, amino acid substitutions) using dense SNP data; we used the current state-of-the-art methods implemented in the software packages HLA*IMP:03 [14], HLA*IMP:02 [15], HIBAG [16] and SNP2HLA [17], and we compared the results obtained for classical HLA alleles with those previously obtained [13] using HLA*IMP:01 [18, 19]. For a detailed description of the HLA imputation and subsequent association analysis performed using the different software packages, see S3 Text. Motyer et al. [14] recently compared the performance of HLA*IMP:03, HLA*IMP:02, HIBAG and SNP2HLA and found that HLA*IMP:03 and HIBAG gave generally the best (similar) levels of performance, achieving high accuracy (in the range ~90–99%, depending on HLA locus). Earlier studies had shown that HLA*IMP:02 and HIBAG performed well in comparison to HLA*IMP:01 [15, 16] while SNP2HLA performed similarly to HLA*IMP:01 [17].

Although based on similar methodological approaches (and, in our investigation, producing largely concordant results, see Results), the output from the four packages that we considered is not fully comparable. HLA*IMP:03 and SNP2HLA provide the posterior probability for each best-imputed allele (although SNP2HLA does not output this quantity directly but rather converts it to a dosage), while HLA*IMP:02 and HIBAG produce a posterior probability for each possible genotype (i.e. for each combination of two alleles, including combinations that have lower probabilities than the best combination). We found this genotype-based output most convenient for averaging over the possible genotype combinations (while allowing appropriately for imputation uncertainty) and for subsequent imputation of amino acid substitutions. Neither HIBAG nor HLA*IMP:02 directly impute amino acid substitutions (as is done by SNP2HLA) but this can be done manually using the peptide sequences of classical alleles available in the IMGT/HLA database (see below). According to Motyer et al. [14], HLA*IMP:03 can also impute amino acid substitutions, but this feature was not enabled in the development version of HLA*IMP:03 to which we were given access. Given (a) the similar performance of the four packages in our data set with respect to classical HLA allele imputation (see Results), (b) the superior performance of HLA*IMP:03 and HIBAG seen by Motyer et al. [14], and (c) the greater convenience of the output from HLA*IMP:02 and HIBAG, for all subsequent detailed modelling of amino acid substitutions we used the imputations derived from HIBAG.

### KIR imputation

We also used the software package KIR*IMP [30] to examine the association between PBC and genes on chromosome 19q13.4 that encode for killer-cell immunoglobulin-like receptors (KIRs). In a previous evaluation of KIR*IMP, Vukcevic et al. [30] showed that KIR imputation using the high-density Illumina Immunochip array is extremely accurate, achieving > 98% accuracy for the majority of loci, at least 95% accuracy for half the remaining loci, and > 90% for the rest. For distinguishing the broad A and B haplotype groups, KIR*IMP achieves ~98.5% accuracy. KIR imputation in our data set was informed by 241 genotyped SNPs in the KIR region that matched the 301 SNPs available in the KIR*IMP training data set. The resulting imputed KIR allele and haplotype frequencies in our PBC data set were found to be extremely close to those seen in the KIR training set. Case-control association analysis was carried out using the Unphased package [44] and via logistic regression in R, with predictors corresponding to KIR*IMP’s “best-guess” genotypes (provided that both inferred alleles had posterior probability > 0.8). Analysis of each individual KIR allele (or haplotype) was performed by using the dosage of each allele (or haplotype) as a single predictor variable. Additionally, a multi-allelic (multi-df) omnibus analysis was also carried out by including predictor variables encoding the multiplicative effects of all alleles at a position (or all haplotypes at a set of positions) in the regression model simultaneously.

### Stepwise logistic regression analysis

Stepwise logistic regression [29] was used to assess the importance of variables while accounting for the effects of other, previously detected, effects. Predictor variables encoding an individual’s estimated dosage of the relevant SNP, HLA classical allele or amino acid substitution were included as predictors in a logistic regression equation in a forward stepwise fashion (and were subsequently considered for removal from the model in a backward stepwise fashion). For full details, see S1 Text.

For our primary analyses, we did not consider it necessary to include additional predictors such as principal component scores in the regression model to account for possible population stratification, as prior analysis of this data set [11, 13, 45] has shown little evidence of population stratification (once appropriate QC has been performed to remove outlying individuals) in this UK-based sample. Similarly we did not consider it necessary to include gender as a covariate–even though gender is known to be important in PBC (the disease is more prevalent in women than in men)–as theoretical arguments dictate that inclusion of gender should not bias the results of association tests between disease and genetic factors (outside of the X/Y chromosomes) as gender is not a *confounder* (it is associated with the disease outcome, but not with the genetic predictors). Indeed, it has been shown [46] that inclusion of known covariates such as gender can even reduce power to detect genetic effects in case-control studies. We subsequently investigated the sensitivity of our results to the inclusion (or not) of principal component scores and gender in the regression model, using the top 10 principal component scores calculated from a pruned (by LD) set of SNPs with SNPs in the extended HLA region removed.

We also investigated the stability of the stepwise selection procedure through a resampling approach motivated by the stability selection procedure of Meinshausen and Buhlmann (2010) [47]. In each of 1000 bootstrap replicates, we randomly selected 2/3 of our cases and 2/3 of our controls to form a new case/control data set and applied stepwise regression to select the top 20 amino acid predictors, noting the order and significance of entry of each predictor in each replicate.

#### Exhaustive and stochastic searches for best combinations of amino acid variables

A drawback of stepwise regression is that it employs a “greedy algorithm” whereby predictors are only included conditional on other predictors that have already been added to the model. This is not equivalent to determining which *combination* of predictors best explain the outcome, and can produce misleading inferences, especially when predictors are highly correlated [32]. To overcome this issue, we performed an exhaustive search via logistic regression of all pairwise and all three-way combinations of individual amino acids (generating 2 df and 3 df tests respectively), and all pairwise and all three-way combinations of amino acid *positions* (generating multi-df tests that include predictor variables encoding the multiplicative effects of all amino acid substitutions at both–or all three–positions in the regression model simultaneously).

Moving beyond 3-way combinations is computationally prohibitive: the 1028 individual amino acids substitutions considered here result in 527,878 pairwise combinations and 180,534,276 three-way combinations; modelling the full set of effects at each of the 368 positions results in 67,528 pairwise tests or 32,953,664 three-way tests, each with potentially many df. To address this issue, we used two Bayesian stochastic search algorithm implementations that have been proposed in the context of genetic fine-mapping to search for the best set of genetic predictors explaining a phenotypic outcome: the GUESSFM package [32] and the FINEMAP package [31]. For GUESSFM we used as input data the “best guess” amino acid designations from HIBAG, while for FINEMAP we used as input the Z-score from the marginal association test of amino acid with disease status. Both packages require specification of various user-defined parameters (such as the expected or maximum number of causal variants) in order to inform the prior distribution of the search space; we performed some limited investigations of the sensitivity of the results to the parameter choices made.

### 3D protein structure modelling

The atomic coordinates (3 dimensional structures) of any HLA molecules carrying or not carrying a significantly associated amino acid residue were determined using comparative protein structure modelling by satisfaction of spatial restraints as implemented by the MODELLER 9.14 computer algorithm [35, 48–50]. HLA proteins of known structure suitable as modelling templates were identified in the Protein Data Bank. The peptide sequences of the target classical alleles were downloaded from the IMGT/HLA database. Sequence alignment was performed with Clustal Omega and manually corrected where necessary. The stereochemical qualities of the modelled structures was verified using the COOT program [51] by assessment of Ramachandran plots and by calculating the least square mean deviation between the template molecule and computed model.

#### Electrostatic potential calculations

The electrostatic potentials around the resulting 3D structures were calculated by numerically solving the Poisson Boltzmann equation using the finite difference method implemented in DelPhi v6.2 [36, 52]. Essential hydrogens were added to each structure using the UCSF Chimera package [37] and the protonated molecule was subsequently used to compute the electrostatic potential. Interior and exterior dielectric constants were set at 2 and 80 respectively, with a solution with charged ions simulated by an assigned ionic strength 0.145. The dielectric boundary between the protein and the solvent was defined by calculating the solvent-accessible surface generated by a rolling probe sphere of radius 1.4 Å. Atomic radii and charges were taken from the CHARMM parameter set [53]. The system was mapped into a 3D cubical grid filled by 80% solute, with the grid dimensions set at 251 grid points per axis (spacing 0.3 Å/grid point), and electrostatic potentials were calculated iteratively starting from the Debye-Hückel boundary conditions. The solvent accessible surface was then colored according to charge using Chimera [37].

## Supporting information

S1 Table Multi-df association tests at the gene level, constructed by including all non-rare (frequency > 0.5% in our data set) alleles at each gene into a combined (omnibus) analysis. (DOCX)

S2 Table Comparison of inclusion or not of covariates on the marginal association results obtained using HIBAG from Table 1. (DOCX)

S3 Table Associations of the lead allele from each haplogroup from Table 1 when considered either marginally, or as part of a 9 variable model (with all lead alleles included simultaneously). (DOCX)

S4 Table Amino acid associations from Table 2 when considered either marginally, or as part of a 5-variable or 9-variable model (with either the top 5 or the top 9 amino acids included simultaneously). (DOCX)

S5 Table Top-ranked residues (as measured by r^2^) in LD with the top five independently associated HLA gene residues identified through stepwise regression. Only the top residues (i.e. those showing the strongest LD with the index residue) are listed. (DOCX)

S6 Table Amino acid residue positions significantly associated (P<0.000136) with PBC in multi-df forward stepwise regression analysis. (DOCX)

S7 Table Results from FINEMAP, for varying values of maximum number of amino acid predictors. (DOCX)

S8 Table Results from GUESSFM analysis of amino acids, for varying values of nexp parameter. (DOCX)

S9 Table Amino acid residues with posterior probability of inclusion >0.8 from snp.picker, applied to GUESSFM results (with nexp = 2). A period (“.”) in the name of the amino acid variable indicates a negative position. An underscore (_) indicates absence of an amino acid residue at that position. Amino acids also appearing in the top five from stepwise regression are shown in ***bold italic***.
(DOCX)

S10 Table Comparison of allelic (= multiplicative), dominant, recessive and genotypic models for top associated classical HLA alleles. Entries corresponding to the preferred model (that with the lowest AIC) in each row are shown in **bold**.
(DOCX)

S11 Table Comparison of allelic (= multiplicative), dominant, recessive and genotypic models for top associated amino acid substitutions. Entries corresponding to the preferred model (that with the lowest AIC) in each row are shown in **bold**.
(DOCX)

S1 Fig Comparison of -log10(P) obtained when testing for marginal association at each amino acid with PBC using logistic regression with different covariates included. None: no covariates included; PCs: the top 10 principal component scores included as covariates; PCsAndGender: the top 10 principal component scores and gender included as covariates.
(PDF)

S2 Fig Association analysis results for individual amino acids (panels (A) and (B)), classical alleles (panels (C) and (D)) and SNPs (panels (E) and (F)) in the extended MHC region, once the top five amino acids (left panels) or the top five variables (right panels)—which correspond to two amino acids, two SNPs and a classical allele—have been included in the regression model.
(PDF)

S3 Fig Association analysis results for individual amino acids while including in the regression model: (A) no other variables; (B) the top five amino acids from stepwise regression; (C) the top five amino acid positions (resulting in multi-df tests at each position) from stepwise regression; (D) the top seven amino acid positions (resulting in multi-df tests at each position) from stepwise regression.
(PDF)

S4 Fig Association analysis results for individual SNPs while including in the regression model: (A) no other variables; (B) the top five amino acids from stepwise regression; (C) the top five amino acid positions (resulting in multi-df tests at each position) from stepwise regression; (D) the top seven amino acid positions (resulting in multi-df tests at each position) from stepwise regression.
(PDF)

S5 Fig Association analysis results for individual classical alleles while including in the regression model: (A) no other variables; (B) the top five amino acids from stepwise regression; (C) the top five amino acid positions (resulting in multi-df tests at each position) from stepwise regression; (D) the top seven amino acid positions (resulting in multi-df tests at each position) from stepwise regression.
(PDF)

S6 Fig Association analysis results for individual amino acids while including in the regression model: (A) no other variables; (B) the top five amino acids from stepwise regression; (C) the four amino acids in the top model from FINEMAP, when limiting to a maximum of four predictors; (D) the five amino acids in the top model from FINEMAP, when limiting to a maximum of five predictors; (E) the six amino acids in the top model from FINEMAP, when limiting to a maximum of six predictors; (F) the seven amino acids in the top model from FINEMAP, when limiting to a maximum of seven predictors.
(PDF)

S7 Fig Association analysis results for individual SNPs while including in the regression model: (A) no other variables; (B) the top five amino acids from stepwise regression; (C) the four amino acids in the top model from FINEMAP, when limiting to a maximum of four predictors; (D) the five amino acids in the top model from FINEMAP, when limiting to a maximum of five predictors; (E) the six amino acids in the top model from FINEMAP, when limiting to a maximum of six predictors; (F) the seven amino acids in the top model from FINEMAP, when limiting to a maximum of seven predictors.
(PDF)

S8 Fig Association analysis results for individual classical alleles while including in the regression model: (A) no other variables; (B) the top five amino acids from stepwise regression; (C) the four amino acids in the top model from FINEMAP, when limiting to a maximum of four predictors; (D) the five amino acids in the top model from FINEMAP, when limiting to a maximum of five predictors; (E) the six amino acids in the top model from FINEMAP, when limiting to a maximum of six predictors; (F) the seven amino acids in the top model from FINEMAP, when limiting to a maximum of seven predictors.
(PDF)

S9 Fig Association analysis results for individual amino acids while including in the regression model: (A) no other variables; (B) the top five amino acids from stepwise regression; (C) the five amino acids with the highest posterior probabilities from GUESSFM when run with with nexp = 2; (D) the five amino acids with the highest posterior probabilities from GUESSFM when run with with nexp = 5; (E) the five amino acids with the highest posterior probabilities from snp.picker, applied following a GUESSFM run with with nexp = 2; (F) the seven amino acids with the highest posterior probabilities from snp.picker, applied following a GUESSFM run with with nexp = 2.
(PDF)

S10 Fig Association analysis results for individual SNPs while including in the regression model: (A) no other variables; (B) the top five amino acids from stepwise regression; (C) the five amino acids with the highest posterior probabilities from GUESSFM when run with with nexp = 2; (D) the five amino acids with the highest posterior probabilities from GUESSFM when run with with nexp = 5; (E) the five amino acids with the highest posterior probabilities from snp.picker, applied following a GUESSFM run with with nexp = 2; (F) the seven amino acids with the highest posterior probabilities from snp.picker, applied following a GUESSFM run with with nexp = 2.
(PDF)

S11 Fig Association analysis results for individual classical alleles while including in the regression model: (A) no other variables; (B) the top five amino acids from stepwise regression; (C) the five amino acids with the highest posterior probabilities from GUESSFM when run with with nexp = 2; (D) the five amino acids with the highest posterior probabilities from GUESSFM when run with with nexp = 5; (E) the five amino acids with the highest posterior probabilities from snp.picker, applied following a GUESSFM run with nexp = 2; (F) the seven amino acids with the highest posterior probabilities from snp.picker, applied following a GUESSFM run with nexp = 2.
(PDF)

S12 Fig Structure and molecular surface electrostatic potential of pocket P4 in HLA-DR molecules. A) The structure and electrostatic potential of HLA-DRB1*08:01. The area within the frame is depicted in expanded form in B and C, and indicates the position of pocket P4 (arrows). All structures were superimposed on HLA-DRB1*08:01 and therefore show the same view. HLA-DRB1 alleles associated with an increased risk of PBC (08:01, 04:03 and 04:04) are shown in panel B whereas those associated with a protective effect (15:01,11:01,11:03 and 11:04) are shown in panel C. Negatively charged potentials (less than 5 kT/e) are coloured red, positively charged (greater than 5 kT/e) blue, and neutral potentials (0 kT/e) are coloured white. Linear interpolation was used to produce the colour for surface potentials between these values.
(PDF)

S13 Fig Structure and molecular surface electrostatic potential of associated residues 57 in HLA-DQ molecules. A) The structure and electrostatic potential of HLA-DQB1*04:02. The area within the frame is depicted in expanded form in B and C, and indicates the position of residue 57 (arrows). All structures were superimposed on HLA-DQB1*04:02 and therefore show the same view. HLA-DQB1 alleles associated with an increased risk of PBC (04:02 and 03:02) are shown in panel B whereas those associated with a protective effect (06:02 and 03:01) are shown in panel C. Negatively charged potentials (less than 5 kT/e) are coloured red, positively charged (greater than 5 kT/e) blue, and neutral potentials (0 kT/e) are coloured white. Linear interpolation was used to produce the colour for surface potentials between these values.
(PDF)

S14 Fig Structure and molecular surface electrostatic potential of associated residues 56, 70 and 71 in HLA-DQ molecules. A) The structure and electrostatic potential of HLA-DQB1*04:02. The area within the frame is depicted in expanded form in B and C, and indicates the position of residues 56, 70 and 71 (arrows). All structures were superimposed on HLA-DQB1*04:02 and therefore show the same view. HLA-DQB1 alleles associated with an increased risk of PBC (04:02 and 03:02) are shown in panel B whereas those associated with a protective effect (06:02 and 03:01) are shown in panel C. Negatively charged potentials (less than 5 kT/e) are coloured red, positively charged (greater than 5 kT/e) blue, and neutral potentials (0 kT/e) are coloured white. Linear interpolation was used to produce the colour for surface potentials between these values.
(PDF)

S1 Text Description of stepwise logistic regression analysis. (DOCX)

S2 Text Description of dominant, recessive, genotypic and interaction models. (DOCX)

S3 Text Details of HLA imputation. (DOCX)

S1 Spreadsheet Quantification of data shown in Fig 1. (XLSX)

S2 Spreadsheet Quantification of data shown in Fig 2. (XLSX)

S3 Spreadsheet Quantification of data shown in Fig 3. (XLSX)

S4 Spreadsheet Quantification of data shown in S1 Fig. (XLSX)

S5 Spreadsheet Quantification of data shown in S2 Fig (Panel B). (XLSX)

S6 Spreadsheet Quantification of data shown in S2 Fig (Panel D). (XLSX)

S7 Spreadsheet Quantification of data shown in S2 Fig (Panel F). (XLSX)

S8 Spreadsheet Quantification of data shown in Fig 4. (XLSX)

S9 Spreadsheet Quantification of data shown in S3 Fig (Panels C and D). (XLSX)

S10 Spreadsheet Quantification of data shown in S4 Fig (Panels C and D). (XLSX)

S11 Spreadsheet Quantification of data shown in S5 Fig (Panel C). (XLSX)

S12 Spreadsheet Quantification of data shown in S5 Fig (Panel D). (XLSX)

S13 Spreadsheet Quantification of data shown in S6 Fig and S9 Fig. (XLSX)

S14 Spreadsheet Quantification of data shown in S7 Fig and S10 Fig. (XLSX)

S15 Spreadsheet Quantification of data shown in S8 Fig and S11 Fig. (XLSX)
