# Supplementary material for: Amino acid residues in five separate HLA genes can explain most of the known associations between the MHC and primary biliary cholangitis
Source: PLoS Genet. 2018 Dec 3;14(12):e1007833. doi: 10.1371/journal.pgen.1007833 (PMC6292650; doi:10.1371/journal.pgen.1007833)
Supplement: S1 Table — (DOCX) [file pgen.1007833.s001.docx]

**S1 Table:** Multi-df association tests at the gene level, constructed by including all non-rare (frequency > 0.5% in our data set) alleles at each gene into a combined (omnibus) analysis

| Gene | df | Primary (marginal) P-value | P-value conditional on all other genes (i.e. with all non-rare alleles at all other genes included in the model) |
| --- | --- | --- | --- |
| HLA-A | 17 | 1.26E-05 | 0.00446 |
| HLA-C | 16 | 4.39E-19 | 0.00026 |
| HLA-B | 25 | 3.97E-28 | 4.02E-07 |
| HLA-DRB1 | 22 | 6.63E-84 | 0.03126 |
| HLA-DQA1 | 12 | 1.89E-86 | 0.00924 |
| HLA-DQB1 | 13 | 3.15E-74 | 0.17971 |
| HLA-DPB1 | 19 | 5.26E-62 | 1.72E-40 |
