# Supplementary material for: Amino acid residues in five separate HLA genes can explain most of the known associations between the MHC and primary biliary cholangitis
Source: PLoS Genet. 2018 Dec 3;14(12):e1007833. doi: 10.1371/journal.pgen.1007833 (PMC6292650; doi:10.1371/journal.pgen.1007833)
Supplement: S2 Table — (DOCX) [file pgen.1007833.s002.docx]

**S2 Table:** Comparison of inclusion or not of covariates on the marginal association results obtained using HIBAG from Table 1

| Haplogroup | Gene | Allele | HIBAG 1.2 (dosages), no covariates | | HIBAG 1.2 (dosages), with 10 PCs included as covariates | | HIBAG 1.2 (dosages), with 10 PCs and gender included as covariates | |
| --- | --- | --- | --- | --- | --- | --- | --- | --- |
|  |  |  | OR | P | OR | P | OR | P |
| 1 | HLA-DQA1 | 04:01 | 3.14 | 3.48E-45 | 3.15 | 9.97E-44 | 3.06 | 2.99E-36 |
|  | HLA-DQB1 | 04:02 | 3.08 | 1.37E-47 | 3.09 | 4.15E-44 | 3.02 | 6.43E-37 |
|  | HLA-DRB1 | 08:01 | 3.26 | 1.25E-45 | 3.27 | 2.90E-44 | 3.19 | 6.97E-37 |
|  | HLA-B | 39:05 | - | - | - | - | - | - |
|  | HLA-B | 39:06 | 2.78 | 1.60E-11 | 2.84 | 2.74E-11 | 2.93 | 2.77E-10 |
| 2 | HLA-DQB1 | 06:02 | 0.66 | 2.14E-16 | 0.66 | 2.15E-17 | 0.64 | 1.22E-17 |
|  | HLA-DRB1 | 15:01 | 0.67 | 4.57E-16 | 0.67 | 5.36E-17 | 0.65 | 3.27E-17 |
|  | HLA-DQA1 | 01:02 | 0.71 | 3.26E-15 | 0.71 | 7.81E-16 | 0.70 | 6.45E-16 |
|  | HLA-B | 07:02 | 0.73 | 3.96E-11 | 0.73 | 1.93E-11 | 0.71 | 7.88E-12 |
| 3 | HLA-DQB1 | 03:01 | 0.70 | 1.93E-15 | 0.71 | 5.70E-16 | 0.70 | 1.13E-14 |
|  | HLA-DRB1 | 11:01 | 0.31 | 1.15E-18 | 0.31 | 5.04E-22 | 0.29 | 2.63E-22 |
|  | HLA-DRB1 | 11:04 | 0.14 | 5.00E-13 | 0.13 | 7.58E-18 | 0.13 | 9.26E-17 |
|  | HLA-DRB1 | 11:03 | 0.002 | 2.15E-10 | 0.002 | 2.64E-13 | 0.002 | 3.81E-12 |
|  | HLA-DQA1 | 05:01 | 0.98 | 0.5895 | 0.98 | 0.5920 | 0.98 | 0.7073 |
|  | HLA-DQA1 | 05:05 | 0.50 | 1.73E-25 | 0.49 | 3.72E-29 | 0.48 | 2.67E-28 |
| 4 | HLA-DRB1 | 04:04 | 1.64 | 4.17E-10 | 1.64 | 1.04E-09 | 1.64 | 1.47E-08 |
|  | HLA-DRB1 | 04:03 | 2.92 | 1.21E-05 | 2.94 | 1.76E-05 | 3.01 | 5.59E-05 |
|  | HLA-DQB1 | 03:02 | 1.34 | 1.04E-09 | 1.34 | 1.83E-09 | 1.33 | 3.34E-08 |
|  | HLA-DQA1 | 03:01 | 1.37 | 1.03E-09 | 1.37 | 1.57E-09 | 1.36 | 3.52E-08 |
| 5 | HLA-DPB1 | 03:01 | 1.80 | 1.60E-26 | 1.80 | 6.52E-26 | 1.83 | 1.36E-23 |
|  | HLA-DPB1 | 06:01 | 9.14 | 3.26E-26 | 9.29 | 1.42E-25 | 9.95 | 3.06E-23 |
| 6 | HLA-DPB1 | 04:01 | 0.75 | 1.29E-18 | 0.75 | 1.55E-18 | 0.74 | 1.31E-17 |
| 7 | HLA-C | 04:01 | 1.37 | 4.01E-10 | 1.37 | 8.41E-10 | 1.37 | 1.22E-08 |
| 8 | HLA-DPB1 | 10:01 | 2.04 | 1.90E-12 | 2.03 | 1.13E-11 | 2.06 | 1.49E-10 |
| 9 | HLA-DPB1 | 17:01 | 2.47 | 5.55E-15 | 2.43 | 1.20E-13 | 2.33 | 4.65E-11 |
| 10 | HLA-DPA | 02:01 | - | - | - | - | - | - |
