# Supplementary material for: Amino acid residues in five separate HLA genes can explain most of the known associations between the MHC and primary biliary cholangitis
Source: PLoS Genet. 2018 Dec 3;14(12):e1007833. doi: 10.1371/journal.pgen.1007833 (PMC6292650; doi:10.1371/journal.pgen.1007833)
Supplement: S3 Table — (DOCX) [file pgen.1007833.s003.docx]

**S3 Table:** Associations of the lead allele from each haplogroup from Table 1 when considered either marginally, or as part of a 9 variable model (with all lead alleles included simultaneously).

| Haplogroup | Gene | Allele | HIBAG 1.2 (dosages), marginal results | | HIBAG 1.2 (dosages), results from 9-variable model | |
| --- | --- | --- | --- | --- | --- | --- |
|  |  |  | OR | P | OR | P |
| 1 | HLA-DQB1 | 04:02 | 3.08 | 1.37E-47 | 2.68 | 3.19E-33 |
| 2 | HLA-DQB1 | 06:02 | 0.66 | 2.14E-16 | 0.73 | 2.83E-09 |
| 3 | HLA-DQA1 | 05:05 | 0.50 | 1.73E-25 | 0.52 | 6.39E-22 |
| 4 | HLA-DRB1 | 04:04 | 1.64 | 4.17E-10 | 1.48 | 2.10E-06 |
| 5 | HLA-DPB1 | 03:01 | 1.80 | 1.60E-26 | 1.53 | 9.06E-12 |
| 6 | HLA-DPB1 | 04:01 | 0.75 | 1.29E-18 | 0.90 | 4.59E-03 |
| 7 | HLA-C | 04:01 | 1.37 | 4.01E-10 | 1.33 | 6.15E-08 |
| 8 | HLA-DPB1 | 10:01 | 2.04 | 1.90E-12 | 1.98 | 1.56E-10 |
| 9 | HLA-DPB1 | 17:01 | 2.47 | 5.55E-15 | 2.52 | 1.40E-14 |
