# Supplementary material for: Amino acid residues in five separate HLA genes can explain most of the known associations between the MHC and primary biliary cholangitis
Source: PLoS Genet. 2018 Dec 3;14(12):e1007833. doi: 10.1371/journal.pgen.1007833 (PMC6292650; doi:10.1371/journal.pgen.1007833)
Supplement: S4 Table — (DOCX) [file pgen.1007833.s004.docx]

**S4 Table:** Amino acid associations from Table 2 when considered either marginally, or as part of a 5-variable or 9-variable model (with either the top 5 or the top 9 amino acids included simultaneously).

| Gene | BP position | Peptide position | Residue | Marginal associations (HIBAG 1.2 dosage) | | Associations when included in 5-amino acid model | | Associations when included in 9-amino acid model | |
| --- | --- | --- | --- | --- | --- | --- | --- | --- | --- |
|  |  |  |  | OR | P | OR | P | OR | P |
| HLA-DPB1 | 33156444 | 11 | L/G | 1.765 | 6.64E-59 | 1.662 | 1.45E-45 | 1.658 | 1.79E-44 |
| HLA-DRB1 | 32659927 | 74 | L | 3.065 | 1.14E-46 | 3.132 | 2.87E-44 | 2.820 | 2.53E-35 |
| HLA-DQB1 | 32740667 | 57 | D | 0.789 | 2.87E-14 | 0.790 | 1.87E-11 | 0.853 | 2.02E-05 |
| HLA-C | 31346910 | 156 | R | 1.255 | 7.16E-12 | 1.281 | 3.24E-13 | 1.227 | 1.88E-08 |
| HLA-DQA1 | 32713244 | -13 | A | 1.997 | 6.02E-29 | 1.614 | 1.42E-11 | 1.838 | 1.13E-16 |
| HLA-B | 31432581 | 45 | T | 1.280 | 2.35E-10 |  |  | 1.191 | 3.48E-05 |
| HLA-DQA1 | 32718440 | 207 | V | 1.400 | 6.35E-16 |  |  | 1.250 | 2.22E-06 |
| HLA-DPB1 | 33156663  33161618 | 84  215 | V  T | 1.713  1.713 | 0.000913  0.000913 |  |  | 2.048  2.048 | 1.07E-05  1.07E-05 |
| HLA-B | 31432689 | 9 | H | 1.181 | 1.07E-05 |  |  | 1.186 | 1.95E-05 |
