# Supplementary material for: Amino acid residues in five separate HLA genes can explain most of the known associations between the MHC and primary biliary cholangitis
Source: PLoS Genet. 2018 Dec 3;14(12):e1007833. doi: 10.1371/journal.pgen.1007833 (PMC6292650; doi:10.1371/journal.pgen.1007833)
Supplement: S5 Table — (DOCX) [file pgen.1007833.s005.docx]

**S5 Table:** Top-ranked residues (as measured by r^2^) in LD with the top five independently associated HLA gene residues identified through stepwise regression. Only the top residues (i.e. those showing the strongest LD with the index residue) are listed.

| Correlation with HLA-DPB1 11L | r^2^ | Correlation with HLA-DRB1 74L | r^2^ | Correlation with HLA-DQB1 57D | r^2^ | Correlation with HLA-C 156R | r^2^ | Correlation with HLA-DQA1 -13A | r^2^ |
| --- | --- | --- | --- | --- | --- | --- | --- | --- | --- |
| HLA-DPB1 11G | 1.000 | HLA-DQA1 69T | 0.909 | HLA-DQB1 57A | 0.492 | HLA-C 114D | 0.774 | HLA-DQA1 -13T | 0.999 |
| HLA-DPB1 8L | 0.716 | HLA-DQB1 56P | 0.888 | HLA-DQB1 77R | 0.447 | HLA-C 114N | 0.774 | HLA-DQA1 53G | 0.702 |
| HLA-DPB1 8V | 0.716 | HLA-DQB1 56L | 0.888 | HLA-DQB1 77T | 0.447 | HLA-C 116F | 0.697 | HLA-DRB1 58A | 0.669 |
| HLA-DPB1 9F | 0.716 | HLA-DQB1 70E | 0.867 | HLA-DQB1 38A | 0.436 | HLA-C 138K | 0.451 | HLA-DRB1 58E | 0.669 |
| HLA-DPB1 57D | 0.680 | HLA-DQB1 71D | 0.867 | HLA-DQB1 38V | 0.436 | HLA-C 138T | 0.451 |  |  |
| HLA-DPB1 57E | 0.680 | HLA-DRB1 13G | 0.699 |  |  |  |  |  |  |
|  |  | HLA-DRB1 16H | 0.699 |  |  |  |  |  |  |
|  |  | HLA-DRB1 16Y | 0.699 |  |  |  |  |  |  |
