# Supplementary material for: Amino acid residues in five separate HLA genes can explain most of the known associations between the MHC and primary biliary cholangitis
Source: PLoS Genet. 2018 Dec 3;14(12):e1007833. doi: 10.1371/journal.pgen.1007833 (PMC6292650; doi:10.1371/journal.pgen.1007833)
Supplement: S6 Table — (DOCX) [file pgen.1007833.s006.docx]

**S6 Table:** Amino acid residue positions significantly associated (P<0.000136) with PBC in multi-df forward stepwise regression analysis.

| Gene | Peptide position | Residues | Stepwise associations  (HIBAG 1.2 dosage) | | |
| --- | --- | --- | --- | --- | --- |
|  |  |  | Order of entry | P to enter | df to enter |
| HLA-DPB1 | 11 | G, L | 1 | 6.64E-59 | 1 |
| HLA-DQB1^a^ | 71 | A, D, K, R, T | 2 | 3.27E-45 | 4 |
| HLA-DRB1 | 58 | A, D, E, F, L, N, S, Y | 3 | 2.67E-23 | 6 |
| HLA-B | 45 | E, G, K, M, T | 4 | 2.17E-10 | 4 |
| HLA-C | 156 | D, L, Q, R, W | 5 | 6.98E-08 | 4 |
| HLA-DQB1 | 203 | -, I, V | 6 | 2.59E-06 | 2 |
| HLA-DRB1 | 13 | F, G, H, R, S Y | 7 | 1.64E-05 | 5 |
| HLA-DPB1 | 215 | -, I, T | 8 | 1.10E-04 | 2 |

^a^ drops out of the model (*P*=0.0189) when a backward step is allowed after step 8
