# Supplementary material for: Amino acid residues in five separate HLA genes can explain most of the known associations between the MHC and primary biliary cholangitis
Source: PLoS Genet. 2018 Dec 3;14(12):e1007833. doi: 10.1371/journal.pgen.1007833 (PMC6292650; doi:10.1371/journal.pgen.1007833)
Supplement: S7 Table — (DOCX) [file pgen.1007833.s007.docx]

**S7 Table:** Results from FINEMAP, for varying values of maximum number of amino acid predictors.

| Maximum number of predictors | Best models (in order of posterior probability) | Posterior probability of model | -log10 Bayes Factor  for model | Best amino acids | Posterior probability of amino acid | -log10 Bayes Factor for amino acid | Predictors tagged (r^2^≥0.9604) by best amino acids |
| --- | --- | --- | --- | --- | --- | --- | --- |
|  |  |  |  |  |  |  |  |
| 1 | ***DPB11L*** | 1.0000 | 54.4973 | ***DPB11L*** | 1.0000 | 12.4960 | DPB11G  ***DPB11L*** |
|  |  |  |  |  |  |  |  |
| 2 | ***DRB74L***+***DPB11L*** | 0.9984 | 91.0747 | ***DPB11L*** | 1.0000 | 12.3867 | DPB11G  ***DPB11L*** |
|  |  |  |  | ***DRB74L*** | 0.9984 | 5.2542 | ***DRB74L*** |
|  |  |  |  |  |  |  |  |
| 3 | ***DRB74L***+***DQB57D***+***DPB11L***  DRB67L+***DRB74L***+***DPB11L***  ***DRB74L***+***DQA.13A***+***DPB11L*** | 0.9244  0.0472  0.0229 | 107.7764  106.4842  106.1699 | ***DPB11L*** | 1.0000 | 10.8631 | DPB11G  ***DPB11L*** |
|  |  |  |  | ***DRB74L*** | 0.9997 | 5.9153 | ***DRB74L*** |
|  |  |  |  | ***DQB57D*** | 0.9246 | 3.5086 | ***DQB57D*** |
|  |  |  |  |  |  |  |  |
| 4 | DRB47F+***DRB74L***+DQB.4V+***DPB11L***  ***C156R***+***DRB74L***+***DQB57D***+***DPB11L***  ***DRB74L***+***DQA.13A***+DQB87F+***DPB11L***  ***C156R***+***DRB74L***+***DQA.13A***+***DPB11L***  ***DRB74L***+***DQA.13A***+DQA207M+***DPB11L***  DRB47F+***DRB74L***+***DQB57D***+***DPB11L***  ***DRB74L***+***DQA.13A***+DQB125G+***DPB11L***  ***DRB74L***+DQA34Q+DQB.4V+***DPB11L***  C152E+***DRB74L***+***DQB57D***+***DPB11L***  C11A+***DRB74L***+***DQA.13A***+***DPB11L*** | 0.3314  0.2727  0.1485  0.0623  0.0403  0.0247  0.0223  0.0194  0.0170  0.0105 | 116.9703  116.8856  116.6217  116.2442  116.0557  115.8421  115.7987  115.7383  115.6792  115.4706 | ***DPB11L*** | 1.0000 | 8.6951 | DPB11G  ***DPB11L*** |
|  |  |  |  | ***DRB74L*** | 0.9980 | 5.1065 | ***DRB74L*** |
|  |  |  |  |  |  |  |  |
| 5 | B69A+DRB47F+***DRB74L***+DQB.4V+***DPB11L***  B45T+***DRB74L***+***DQA.13A***+DQB87F+***DPB11L***  ***C156R***+***DRB74L***+***DQA.13A***+DQB87F+***DPB11L***  ***C156R***+***DRB74L***+***DQA.13A***+***DQB57D***+***DPB11L***  C11A+***DRB74L***+***DQA.13A***+DQB87F+***DPB11L***  ***C156R***+DRB67L+***DRB74L***+DQB13G+***DPB11L***  DRB47F+***DRB74L***+DQB.4V+DQB67V+***DPB11L***  ***C156R***+***DRB74L***+***DQA.13A***+DQA207M+***DPB11L***  B45T+***DRB74L***+***DQA.13A***+DQB125G+***DPB11L***  B45T+***DRB74L***+***DQA.13A***+DQA207M+***DPB11L***  B69A+***DRB74L***+DQA34Q+DQB.4V+***DPB11L***  B69A+DRB47F+***DRB74L***+DQB.18A+***DPB11L***  ***C156R***+***DRB74L***+***DQA.13A***+DQB125G+***DPB11L***  C11A+***DRB74L***+***DQA.13A***+DQA207M+***DPB11L*** | 0.4088  0.1467  0.0903  0.0733  0.0491  0.0373  0.0263  0.0203  0.0175  0.0157  0.0134  0.0130  0.0125  0.0102 | 125.2265  124.7812  124.5707  124.4803  124.3062  124.1872  124.0347  123.9227  123.8575  123.8102  123.7420  123.7299  123.7114  123.6242 | ***DPB11L*** | 1.0000 | 7.6703 | DPB11G  ***DPB11L*** |
|  |  |  |  | ***DRB74L*** | 0.9964 | 4.8347 | ***DRB74L*** |
|  |  |  |  |  |  |  |  |
| 6 | ***C156R***+***DRB74L***+DQA175E+DQB.4V+DQB71T+***DPB11L***  ***C156R***+DRB47F+***DRB74L***+DQB.4V+DQB67V+***DPB11L***  ***C156R***+***DRB74L***+***DQA.13A***+DQB26G+DQB57A+***DPB11L***  ***C156R***+DRB47F+***DRB74L***+DQB.40+DQB71T+***DPB11L***  ***C156R***+***DRB74L***+DQA175E+DQB.4V+DQB77R+***DPB11L***  B45T+DRB47F+***DRB74L***+DQB.4V+DQB67V+***DPB11L***  ***C156R***+DRB47F+***DRB74L***+DQB.4V+DQB37Y+***DPB11L*** | 0.4449  0.3250  0.0258  0.0251  0.0157  0.0121  0.0121 | 132.0300  131.8936  130.7934  130.7805  130.5777  130.4661  130.4627 | ***DPB11L*** | 0.9999 | 6.3181 | DPB11G  ***DPB11L*** |
|  |  |  |  | ***DRB74L*** | 0.9982 | 5.1442 | ***DRB74L*** |
|  |  |  |  | ***C156R*** | 0.9330 | 3.5414 | ***C156R*** |
|  |  |  |  | DQB.4V | 0.8884 | 3.2980 | DQB.27A  DQB.21G  DQB.9M  DQB.6T  DQB.5P  DQB.4V |
|  |  |  |  | DQB71T | 0.5047 | 2.4054 | DQB71T  DQB74E  DQB75V  DQB75L |
|  |  |  |  |  |  |  |  |
| 7 | B45T+***C156R***+***DRB74L***+DQA175E+DQB.4V+DQB71T+***DPB11L***  B45T+C116S+***DRB74L***+DQA175E+DQB.4V+DQB71T+***DPB11L***  B9H+***C156R***+***DRB74L***+DQA175E+DQB.4V+DQB71T+***DPB11L***  B45T+C116S+DRB47F+***DRB74L***+DQB.4V+DQB67V+***DPB11L***  B9Y+***C156R***+DRB47F+***DRB74L***+DQB.4V+DQB67V+***DPB11L***  C113H+***C156R***+***DRB74L***+DQA175E+DQB.4V+DQB71T+***DPB11L***  B9Y+***C156R***+***DRB74L***+DQA175E+DQB.4V+DQB71T+***DPB11L***  B45T+***C156R***+DRB47F+***DRB74L***+DQB.4V+DQB67V+***DPB11L***  C113H+***C156R***+DRB47F+***DRB74L***+DQB.4V+DQB67V+***DPB11L***  B9H+***C156R***+DRB47F+***DRB74L***+DQB.4V+DQB67V+***DPB11L***  B45T+C116S+***DRB74L***+***DQA.13A***+DQB26G+DQB57A+***DPB11L***  B45T+C116S+DRB58A+***DRB74L***+DQB26G+DQB57A+***DPB11L***  B9H+***C156R***+***DRB74L***+***DQA.13A***+DQB26G+DQB57A+***DPB11L***  ***C156R***+***DRB74L***+DQA175E+DQB.4V+DQB71T+***DPB11L***+DPB215T  B9Y+***C156R***+DRB47F+***DRB74L***+DQB.40+DQB71T+***DPB11L*** | 0.2093  0.0937  0.0917  0.0375  0.0368  0.0359  0.0275  0.0254  0.0242  0.0234  0.0211  0.0172  0.0151  0.0113  0.0100 | 135.9780  135.6292  135.6194  135.2308  135.2236  135.2126  135.0961  135.0621  135.0418  135.0273  134.9810  134.8925  134.8374  134.7100  134.6588 | ***DPB11L*** | 0.9841 | 4.1894 | DPB11G  ***DPB11L*** |
|  |  |  |  | ***DRB74L*** | 0.9841 | 4.1893 | ***DRB74L*** |
|  |  |  |  | DQB.4V | 0.8046 | 3.0118 | DQB.27A  DQB.21G  DQB.9M  DQB.6T  DQB.5P  DQB.4V |
|  |  |  |  | ***C156R*** | 0.7279 | 2.8244 | ***C156R*** |
|  |  |  |  | DQB71T | 0.6033 | 2.5792 | DQB71T  DQB74E  DQB75V  DQB75L |
|  |  |  |  | DQA175E | 0.5612 | 2.504 | DQA175E |
|  |  |  |  | B45T | 0.5105 | 2.4153 | B45T |
|  |  |  |  |  |  |  |  |
| 8 | B9H+C113H+***C156R***+***DRB74L***+DQA175E+DQB.4V+DQB71T+***DPB11L***  B9Y+C113H+***C156R***+DRB47F+***DRB74L***+DQB.4V+DQB67V+***DPB11L***  B9Y+C113H+***C156R***+***DRB74L***+DQA175E+DQB.4V+DQB71T+***DPB11L***  B9Y+B45T+***C156R***+***DRB74L***+DQA175E+DQB.4V+DQB71T+***DPB11L***  B45T+***C156R***+***DRB74L***+DQA175E+DQB.4V+DQB71T+***DPB11L***+DPB215T  B9H+C113H+***C156R***+***DRB74L***+***DQA.13A***+DQB26G+DQB57A+***DPB11L***  B45T+***C156R***+***DRB74L***+DQA175E+DQB.4V+DQB71T+***DPB11L***  B45T+B147W+***C156R***+***DRB74L***+DQA175E+DQB.4V+DQB71T+***DPB11L***  B45T+C91G+C116S+***DRB74L***+DQA175E+DQB.4V+DQB71T+***DPB11L***  A76A+B9Y+***C156R***+***DRB74L***+DQA175E+DQB.4V+DQB71T+***DPB11L***  B9H+C113H+***C156R***+DRB47F+***DRB74L***+DQB.4V+DQB67V+***DPB11L***  B9Y+C113H+***C156R***+DRB47F+***DRB74L***+DQB.40+DQB71T+***DPB11L***  B9H+B45T+***C156R***+***DRB74L***+DQA175E+DQB.4V+DQB71T+***DPB11L***  B45T+C116S+***DRB74L***+DQA175E+DQB.4V+DQB71T+***DPB11L***+DPB215T  A76A+B9Y+***C156R***+DRB47F+***DRB74L***+DQB.4V+DQB67V+***DPB11L***  B45T+***C156R***+***DRB74L***+DQA175E+DQB.4V+DQB71T+DPB8V+DPB194Q  B45T+***C156R***+C156Q+***DRB74L***+DQA175E+DQB.4V+DQB71T+***DPB11L***  B9H+***C156R***+***DRB74L***+DQA175E+DQB.4V+DQB71T+***DPB11L***+DPB215T | 0.0707  0.0530  0.0521  0.0456  0.0235  0.0195  0.0195  0.0188  0.0185  0.0184  0.0183  0.0148  0.0136  0.0136  0.0112  0.0110  0.0106  0.0103 | 139.1348  139.0102  139.0026  138.9446  138.6573  138.5755  135.9780  138.5609  138.5524  138.5506  138.5486  138.4572  138.4191  138.4180  138.3356  138.3279  138.3102  138.2970 | ***DRB74L*** | 0.9874 | 4.2915 | ***DRB74L*** |
|  |  |  |  | ***DPB11L*** | 0.9415 | 3.6037 | DPB11G  ***DPB11L*** |
|  |  |  |  | ***C156R*** | 0.7963 | 2.9891 | ***C156R*** |
|  |  |  |  | DQB.4V | 0.777 | 2.9393 | DQB.27A  DQB.21G  DQB.9M  DQB.6T  DQB.5P  DQB.4V |
|  |  |  |  | DQB71T | 0.598 | 2.5695 | DQB71T  DQB74E  DQB75V  DQB75L |
|  |  |  |  | DQA175E | 0.5324 | 2.4534 | DQA175E |

“Best models” are defined as all those with posterior probability ≥0.01. “Best amino acids” are defined as all those with posterior probability of inclusion ≥0.5. Amino acids also appearing in the top five from stepwise regression are shown in ***bold italic***. A period (“.”) in the name of the amino acid indicates a negative position.
