# Supplementary material for: Amino acid residues in five separate HLA genes can explain most of the known associations between the MHC and primary biliary cholangitis
Source: PLoS Genet. 2018 Dec 3;14(12):e1007833. doi: 10.1371/journal.pgen.1007833 (PMC6292650; doi:10.1371/journal.pgen.1007833)
Supplement: S8 Table — (DOCX) [file pgen.1007833.s008.docx]

**S8 Table:** Results from GUESSFM analysis of amino acids, for varying values of nexp parameter.

| nexp | Size of best models | Best models (in order of posterior probability) | Posterior probability of model | Best amino acids | Marginal  posterior probability of inclusion of amino acid | Predictors tagged (r^2^≥0.9604) by best amino acids |
| --- | --- | --- | --- | --- | --- | --- |
|  |  |  |  |  |  |  |
| 1 | 7 | B45T+***DPB11G***+DQA175E+DQB26G+DRB67I+DRB74R+DRB78Y | 0.9954 | DRB74R | 1.0000 | DRB74R  DRB77T  DRB77N  DQA53Q |
|  |  |  |  | DRB78Y | 1.0000 | DRB78Y  DRB78V |
|  |  |  |  | DRB67I | 1.0000 | DRB67I |
|  |  |  |  | DQA175E | 1.0000 | DQA175E |
|  |  |  |  | DQB26G | 1.0000 | DQB26G  DQB74S |
|  |  |  |  | ***DPB11G*** | 1.0000 | ***DPB11G***  DPB11L |
|  |  |  |  | B45T | 0.9980 | B45T |
|  |  |  |  |  |  |  |
| 2 | 9  9  8  9  9  9  9  9  8  9  9  9  9  8  9 | B9Y+C113Y+***C156R***+***DPB11G***+DPB84V+DQA34E+DQB.270+DQB38V+***DRB74L***  B9Y+C113Y+***C156R***+***DPB11G***+DPB84V+DQA34E+DQB.270+DQB74E+***DRB74L***  B9Y+C113Y+***C156R***+***DPB11G***+DQA34E+DQB.270+DQB38V+***DRB74L***  B9Y+***C156R***+***DPB11G***+DPB65I+DPB84V+DQA34E+DQB.270+DQB38V+***DRB74L***  B9Y+C113Y+***C156R***+***DPB11G***+DPB84V+DQA34E+DQB.27A+DQB66D+***DRB74L***  A76A+B9Y+C113Y+***C156R***+***DPB11G***+DQA34E+DQB.270+DQB38V+***DRB74L***  B9Y+***C156R***+***DPB11G***+DPB65I+DPB84V+DQA34E+DQB.270+DQB74E+***DRB74L***  B45T+B9Y+***C156R***+***DPB11G***+DPB84V+DQA34E+DQB.270+DQB38V+***DRB74L***  B9Y+C113Y+***C156R***+***DPB11G***+DQA34E+DQB.270+DQB74E+***DRB74L***  B45T+B9Y+***C156R***+***DPB11G***+DPB84V+DQA34E+DQB.270+DQB74E+***DRB74L***  B9Y+C113Y+***C156R***+***DPB11G***+DPB84V+DQB.27A+DQB66D+DRB47Y+***DRB74L***  A76A+B9Y+***C156R***+***DPB11G***+DPB84V+DQA34E+DQB.270+DQB38V+***DRB74L***  A76A+B9Y+C113Y+***C156R***+***DPB11G***+DQA34E+DQB.27A+DQB66D+***DRB74L***  B9Y+***C156R***+***DPB11G***+DPB84V+DQA34E+DQB.270+DQB38V+***DRB74L***  B9Y+C113Y+***C156R***+***DPB11G***+DPB84V+DQB.270+DQB38V+DRB47Y+***DRB74L*** | 0.2441  0.1180  0.0500  0.0459  0.0335  0.0334  0.0252  0.0239  0.0180  0.0169  0.0168  0.0157  0.0139  0.0124  0.0114 | ***DPB11G*** | 1.0000 | ***DPB11G***  DPB11L |
|  |  |  |  | ***DRB74L*** | 0.9999 | ***DRB74L*** |
|  |  |  |  | B9Y | 0.9995 | B9Y |
|  |  |  |  | ***C156R*** | 0.9983 | ***C156R*** |
|  |  |  |  | DQA34E | 0.9915 | DQA34E  DQA34Q |
|  |  |  |  | DQB.270 | 0.9743 | DQB.270  DQB.210  DQB.180  DQB.100  DQB.90  DQB.60  DQB.50  DQB.40 |
|  |  |  |  | C113Y | 0.9544 | C113Y  C113H |
|  |  |  |  | DPB84V | 0.9384 | DPB84V  DPB215T |
|  |  |  |  | DQB38V | 0.7690 | DQB38V  DQB38A  DQB77R  DQB77T |
|  |  |  |  |  |  |  |
| 3 | 10  10  9  10  9  10  10  10  10  8  10  10  9 | B9Y+C113Y+***C156R***+***DPB11G***+DPB65I+DPB84V+DQA34E+DQB.270+DQB38V+***DRB74L***  B9Y+C113Y+***C156R***+***DPB11G***+DPB65I+DPB84V+DQA34E+DQB.270+DQB74E+***DRB74L***  B9Y+C113Y+***C156R***+***DPB11G***+DPB84V+DQA34E+DQB.270+DQB38V+***DRB74L***  A76A+B9Y+C113Y+***C156R***+***DPB11G***+DPB84V+DQA34E+DQB.270+DQB38V+***DRB74L***  B9Y+C113Y+***C156R***+***DPB11G***+DPB84V+DQA34E+DQB.270+DQB74E+***DRB74L***  B45T+B9Y+***C156R***+***DPB11G***+DPB65I+DPB84V+DQA34E+DQB.270+DQB38V+***DRB74L***  B45T+B9Y+***C156R***+***DPB11G***+DPB65I+DPB84V+DQA34E+DQB.270+DQB74E+***DRB74L***  B9Y+C113Y+***C156R***+***DPB11G***+DPB65I+DPB84V+DQA34E+DQB.27A+DQB66D+***DRB74L***  A76A+B9Y+***C156R***+***DPB11G***+DPB65I+DPB84V+DQA34E+DQB.270+DQB38V+***DRB74L***  B9Y+C113Y+***C156R***+***DPB11G***+DQA34E+DQB.270+DQB38V+***DRB74L***  A76A+B9Y+C113Y+***C156R***+***DPB11G***+DPB84V+DQA34E+DQB.270+DQB74E+***DRB74L***  A76A+B9Y+C113Y+***C156R***+***DPB11G***+DPB84V+DQA34E+DQB.27A+DQB66D+***DRB74L***  B9Y+***C156R***+***DPB11G***+DPB65I+DPB84V+DQA34E+DQB.270+DQB38V+***DRB74L*** | 0.2059  0.1157  0.0671  0.0387  0.0324  0.0242  0.0195  0.0191  0.0186  0.0155  0.0147  0.0127  0.0126 | ***DPB11G*** | 1.0000 | ***DPB11G***  DPB11L |
|  |  |  |  | ***DRB74L*** | 0.9999 | ***DRB74L*** |
|  |  |  |  | ***C156R*** | 0.9999 | ***C156R*** |
|  |  |  |  | DQA34E | 0.9992 | DQA34E  DQA34Q |
|  |  |  |  | B9Y | 0.9988 | B9Y |
|  |  |  |  | DQB.270 | 0.9986 | DQB.270  DQB.210  DQB.180  DQB.100  DQB.90  DQB.60  DQB.50  DQB.40 |
|  |  |  |  | DPB84V | 0.9976 | DPB84V  DPB215T |
|  |  |  |  | C113Y | 0.9819 | C113Y  C113H |
|  |  |  |  | DPB65I | 0.9019 | DPB65I  DPB65L |
|  |  |  |  | DQB38V | 0.8437 | DQB38V  DQB38A  DQB77R  DQB77T |
|  |  |  |  |  |  |  |
| 4 | 10  11  11  10  11  9  11  11  11  11  11  10 | B9Y+C113Y+***C156R***+***DPB11G***+DPB65I+DPB84V+DQA34E+DQB.270+DQB38V+***DRB74L***  A76A+B9Y+C113Y+***C156R***+***DPB11G***+DPB65I+DPB84V+DQA175E+DQA50L+DQB.27A+DQB38V  A76A+B9Y+C113Y+***C156R***+***DPB11G***+DPB65I+DPB84V+DQA34E+DQB.270+DQB38V+***DRB74L***  B9Y+C113Y+***C156R***+***DPB11G***+DPB65I+DPB84V+DQA34E+DQB.270+DQB74E+***DRB74L***  A76A+B9Y+C113Y+***C156R***+***DPB11G***+DPB65I+DPB84V+DQA175E+DQB.27A+DQB38V+***DRB74L***  B9Y+C113Y+***C156R***+***DPB11G***+DPB84V+DQA34E+DQB.270+DQB38V+***DRB74L***  A76A+B9Y+C113Y+***C156R***+***DPB11G***+DPB65I+DPB84V+DQA34E+DQB.270+DQB74E+***DRB74L***  A76A+B9Y+C113Y+***C156R***+***DPB11G***+DPB65I+DPB84V+DQA175E+DQA50L+DQB.27A+DQB74E  A76A+B9Y+C113Y+***C156R***+***DPB11G***+DPB65I+DPB84V+DQB13G+DQB38V+DRB13H+***DRB74L***  A76A+B45T+B9Y+***C156R***+***DPB11G***+DPB65I+DPB84V+DQA175E+DQA50L+DQB.27A+DQB38V  A76A+B45T+B9Y+***C156R***+***DPB11G***+DPB65I+DPB84V+DQB13G+DQB38V+DRB13H+***DRB74L***  A76A+B9Y+C113Y+***C156R***+***DPB11G***+DPB84V+DQA34E+DQB.270+DQB38V+***DRB74L*** | 0.0549  0.0374  0.0360  0.0308  0.0244  0.0194  0.0163  0.0137  0.0131  0.0113  0.0110  0.0103 | ***DPB11G*** | 1.0000 | ***DPB11G***  DPB11L |
|  |  |  |  | DPB84V | 0.9974 | DPB84V  DPB215T |
|  |  |  |  | B9Y | 0.9862 | B9Y |
|  |  |  |  | ***C156R*** | 0.9789 | ***C156R*** |
|  |  |  |  | DPB65I | 0.9240 | DPB65I  DPB65L |
|  |  |  |  | C113Y | 0.8582 | C113Y  C113H |
|  |  |  |  | ***DRB74L*** | 0.7765 | ***DRB74L*** |
|  |  |  |  | DQB38V | 0.7172 | DQB38V  DQB38A  DQB77R  DQB77T |
|  |  |  |  | DQA34E | 0.5891 | DQA34E  DQA34Q |
|  |  |  |  | DQB.270 | 0.5763 | DQB.270  DQB.210  DQB.180  DQB.100  DQB.90  DQB.60  DQB.50  DQB.40 |
|  |  |  |  | A76A | 0.5028 | A76A |
|  |  |  |  |  |  |  |
| 5 | 11  10  11  11  11  12  11 | A76A+B9Y+C113Y+***C156R***+DPB35Y+DPB76V+DPB8L+DQA175E+DQA50L+DQB.27A+DQB38V  B9Y+C113Y+***C156R***+DPB35Y+DPB76V+DPB8L+DQA34E+DQB.270+DQB38V+***DRB74L***  A76A+B9Y+C113Y+***C156R***+DPB35Y+DPB76V+DPB8L+DQA175E+DQB.27A+DQB38V+***DRB74L***  A76A+B9Y+C113Y+***C156R***+DPB35Y+DPB76V+DPB8L+DQA34E+DQB.270+DQB38V+***DRB74L***  A76A+B9Y+C113Y+***C156R***+DPB35Y+DPB76V+DPB8L+DQA50L+DQB.27A+DQB38V+***DRB74L***  A76A+B70K+B9Y+C113Y+***C156R***+DPB35Y+DPB76V+DPB8L+DQA34E+DQB.270+DQB38V+***DRB74L***  A76A+B9Y+C113Y+***C156R***+***DPB11G***+DPB65I+DPB84V+DQA175E+DQA50L+DQB.27A+DQB38V | 0.0219  0.0202  0.0156  0.0154  0.0132  0.0114  0.0102 | B9Y | 0.9954 | B9Y |
|  |  |  |  | ***C156R*** | 0.9627 | ***C156R*** |
|  |  |  |  | C113Y | 0.9232 | C113Y  C113H |
|  |  |  |  | DQB38V | 0.8117 | DQB38V  DQB38A  DQB77R  DQB77T |
|  |  |  |  | ***DRB74L*** | 0.7798 | ***DRB74L*** |
|  |  |  |  | DPB8L | 0.7664 | DPB8V  DPB8L  DPB9F |
|  |  |  |  | DPB35Y | 0.7001 | DPB35Y |
|  |  |  |  | DPB76V | 0.6999 | DPB76V |
|  |  |  |  | A76A | 0.5996 | A76A |
|  |  |  |  | DQA34E | 0.5882 | DQA34E  DQA34Q |
|  |  |  |  | DQB.270 | 0.5556 | DQB.270  DQB.210  DQB.180  DQB.100  DQB.90  DQB.60  DQB.50  DQB.40 |

“nexp”= expected number of causal variants assumed in Bayesian variable selection procedure. “size”= number of predictors included. “Best models” are defined as all those with posterior probability ≥0.01; “Best amino acids” are defined as all those with marginal posterior probability of inclusion ≥0.5. Amino acids also appearing in the top five from stepwise regression are shown in ***bold italic***. A period (“.”) in the name of the amino acid indicates a negative position.
