# Supplementary material for: Amino acid residues in five separate HLA genes can explain most of the known associations between the MHC and primary biliary cholangitis
Source: PLoS Genet. 2018 Dec 3;14(12):e1007833. doi: 10.1371/journal.pgen.1007833 (PMC6292650; doi:10.1371/journal.pgen.1007833)
Supplement: S9 Table — A period (“.”) in the name of the amino acid variable indicates a negative position. An underscore (_) indicates absence of an amino acid residue at that position. Amino acids also appearing in the top five from stepwise regression are shown in bold italic. (DOCX) [file pgen.1007833.s009.docx]

**S9 Table:** Amino acid residues with posterior probability of inclusion >0.8 from snp.picker, applied to GUESSFM results (with nexp=2). A period (“.”) in the name of the amino acid variable indicates a negative position. An underscore (_) indicates absence of an amino acid residue at that position. Amino acids also appearing in the top five from stepwise regression are shown in ***bold italic***.

| Amino acid  variable name | Amino acid | Posterior probability of inclusion |
| --- | --- | --- |
| ***DPB11G*** | HLA-DPB1 11G | 1.0000 |
| ***DRB74L*** | HLA-DRB1 74L | 1.0000 |
| DQA34E | HLA-DQA1 34E | 1.0000 |
| DQB.270 | HLA-DQB1 -27_ | 1.0000 |
| ***C156R*** | HLA-C 156R | 0.9974 |
| B9Y | HLA-B 9Y | 0.9924 |
| DQB38V | HLA-DQB1 38V | 0.8817 |
