# Supplementary material for: Amino acid residues in five separate HLA genes can explain most of the known associations between the MHC and primary biliary cholangitis
Source: PLoS Genet. 2018 Dec 3;14(12):e1007833. doi: 10.1371/journal.pgen.1007833 (PMC6292650; doi:10.1371/journal.pgen.1007833)
Supplement: S10 Table — Entries corresponding to the preferred model (that with the lowest AIC) in each row are shown in bold. (DOCX) [file pgen.1007833.s010.docx]

**S10 Table:** Comparison of allelic (=multiplicative), dominant, recessive and genotypic models for top associated classical HLA alleles. Entries corresponding to the preferred model (that with the lowest AIC) in each row are shown in **bold**.

|  |  |  |  |  | | |  | | |  | | |  | | | | | Implied odds ratios (ORs) | | | | | | | |
| --- | --- | --- | --- | --- | --- | --- | --- | --- | --- | --- | --- | --- | --- | --- | --- | --- | --- | --- | --- | --- | --- | --- | --- | --- | --- |
| Haplogroup | Gene | Allele |  | Allelic (multiplicative) model | | | Dominant model | | | Recessive model | | | Genotypic model | | | | | Allelic model | | Dominant model | | Recessive model | | Genotypic model | |
|  |  |  | Null AIC | AIC | lnOR | SE | AIC | lnOR | SE | AIC | lnOR | SE | AIC | lnOR1 | SE1 | lnOR2 | SE2 | OR1 | OR2 | OR1 | OR2 | OR1 | OR2 | OR1 | OR2 |
| 1 | HLA-DQA1 | 04:01 | 12832.89 | 12642.24 | 1.13 | 0.08 | **12637.80** | **1.17** | **0.08** | 12832.76 | 0.91 | 0.61 | 12639.71 | 1.17 | 0.08 | 0.99 | 0.61 | 3.09 | 9.54 | **3.21** | **3.21** | 1 | 2.48 | 3.22 | 2.69 |
|  | HLA-DQB1 | 04:02 | 12832.89 | 12643.06 | 1.11 | 0.08 | **12639.70** | **1.15** | **0.08** | 12831.48 | 1.09 | 0.58 | 12641.70 | 1.15 | 0.08 | 1.17 | 0.58 | 3.03 | 9.20 | **3.15** | **3.15** | 1 | 2.98 | 3.15 | 3.23 |
|  | HLA-DRB1 | 08:01 | 12832.89 | 12642.24 | 1.14 | 0.08 | **12637.66** | **1.18** | **0.08** | 12832.76 | 0.91 | 0.61 | 12639.56 | 1.18 | 0.08 | 0.99 | 0.61 | 3.12 | 9.74 | **3.25** | **3.25** | 1 | 2.48 | 3.26 | 2.69 |
|  | HLA-B | 39:05^a^ | - | - | - | - | - | - | - | - | - | - | - | - | - | - | - | - | - | - | - | - | - | - | - |
|  | HLA-B | 39:06^a^ | 12832.89 | **12789.26** | **0.98** | **0.14** | **12789.26** | **0.98** | **0.14** | NA | NA | NA | NA | NA | NA | NA | NA | **2.66** | **7.06** | **2.66** | **2.66** | 1 | NA | NA | NA |
| 2 | HLA-DQB1 | 06:02 | 12832.89 | 12762.48 | -0.41 | 0.05 | **12758.93** | **-0.46** | **0.05** | 12828.75 | -0.42 | 0.18 | 12760.83 | -0.46 | 0.06 | -0.52 | 0.18 | 0.66 | 0.44 | **0.63** | **0.63** | 1 | 0.65 | 0.63 | 0.60 |
|  | HLA-DRB1 | 15:01 | 12832.89 | 12764.63 | -0.40 | 0.05 | **12761.18** | **-0.45** | **0.05** | 12828.86 | -0.42 | 0.18 | 12763.08 | -0.45 | 0.06 | -0.51 | 0.18 | 0.67 | 0.45 | **0.63** | **0.63** | 1 | 0.66 | 0.64 | 0.60 |
|  | HLA-DQA1 | 01:02 | 12832.89 | **12770.14** | **-0.33** | **0.04** | 12778.89 | -0.36 | 0.05 | 12810.42 | -0.65 | 0.14 | 12771.67 | -0.31 | 0.05 | -0.74 | 0.14 | **0.72** | **0.51** | 0.70 | 0.70 | 1 | 0.52 | 0.73 | 0.48 |
|  | HLA-B | 07:02 | 12832.89 | 12788.79 | -0.31 | 0.05 | 12800.13 | -0.3 | 0.05 | 12803.64 | -1.04 | 0.21 | **12783.34** | **-0.25** | **0.05** | **-1.10** | **0.21** | 0.73 | 0.53 | 0.74 | 0.74 | 1 | 0.35 | **0.78** | **0.33** |
| 3 | HLA-DQB1 | 03:01 | 12832.89 | **12772.38** | **-0.33** | **0.04** | 12779.44 | -0.35 | 0.05 | 12814.56 | -0.63 | 0.15 | 12774.16 | -0.32 | 0.05 | -0.72 | 0.15 | **0.72** | **0.52** | 0.70 | 0.70 | 1 | 0.53 | 0.73 | 0.49 |
|  | HLA-DRB1 | 11:01 | 12832.89 | **12751.02** | **-0.79** | **0.09** | 12751.17 | -0.8 | 0.09 | 12833.03 | -1.21 | 1.05 | 12752.94 | -0.79 | 0.10 | -1.27 | 1.05 | **0.45** | **0.21** | 0.45 | 0.45 | 1 | 0.30 | 0.45 | 0.28 |
|  | HLA-DRB1 | 11:04 | 12832.89 | **12823.12** | **-0.70** | **0.22** | **12823.12** | **-0.7** | **0.22** | NA | NA | NA | NA | NA | NA | NA | NA | **0.50** | **0.25** | **0.5** | **0.50** | 1 | NA | NA | NA |
|  | HLA-DRB1 | 11:03 | 12832.89 | **12830.25** | **-11.48** | **114.81** | **12830.25** | **-11.48** | **114.81** | NA | NA | NA | NA | NA | NA | NA | NA | **0** | **0** | **0** | **0** | 1 | NA | NA | NA |
|  | HLA-DQA1 | 05:01^b^ | 12832.89 | 12834.60 | -0.02 | 0.04 | 12834.88 | 0 | 0.05 | **12831.78** | **-0.30** | **0.17** | 12833.66 | 0.02 | 0.05 | -0.29 | 0.17 | 0.98 | 0.95 | 1 | 1 | **1** | **0.74** | 1.02 | 0.75 |
|  | HLA-DQA1 | 05:05^b^ | 12832.89 | **12710.62** | **-0.69** | **0.07** | 12711.04 | -0.72 | 0.07 | 12823.99 | -0.98 | 0.34 | 12711.79 | -0.71 | 0.07 | -1.07 | 0.34 | **0.50** | **0.25** | 0.49 | 0.49 | 1 | 0.37 | 0.49 | 0.34 |
| 4 | HLA-DRB1 | 04:04 | 12832.89 | 12797.15 | 0.41 | 0.07 | **12795.25** | **0.43** | **0.07** | 12834.78 | -0.21 | 0.65 | 12796.33 | 0.44 | 0.07 | -0.16 | 0.65 | 1.51 | 2.27 | **1.54** | **1.54** | 1 | 0.81 | 1.55 | 0.85 |
|  | HLA-DRB1 | 04:03 | 12832.89 | **12834.63** | **0.15** | **0.30** | **12834.63** | **0.15** | **0.30** | NA | NA | NA | NA | NA | NA | NA | NA | **1.16** | **1.35** | **1.16** | **1.16** | 1 | NA | NA | NA |
|  | HLA-DQB1 | 03:02 | 12832.89 | 12798.40 | 0.29 | 0.05 | **12792.71** | **0.33** | **0.05** | 12834.88 | 0.01 | 0.21 | 12793.27 | 0.34 | 0.05 | 0.09 | 0.21 | 1.33 | 1.77 | **1.39** | **1.39** | 1 | 1.01 | 1.41 | 1.09 |
|  | HLA-DQA1 | 03:01 | 12832.89 | 12800.27 | 0.28 | 0.05 | **12795.19** | **0.32** | **0.05** | 12834.88 | 0.02 | 0.22 | 12795.92 | 0.34 | 0.05 | 0.09 | 0.22 | 1.33 | 1.76 | **1.38** | **1.38** | 1 | 1.02 | 1.40 | 1.09 |
| 5 | HLA-DPB1 | 03:01 | 12832.89 | **12713.50** | **0.50** | **0.04** | 12715.01 | 0.53 | 0.05 | 12823.12 | 0.69 | 0.20 | 12714.58 | 0.52 | 0.05 | 0.83 | 0.20 | **1.64** | **2.70** | 1.70 | 1.70 | 1 | 2.00 | 1.67 | 2.29 |
|  | HLA-DPB1 | 06:01 | 12832.89 | **12828.50** | **0.91** | **0.35** | **12828.50** | **0.91** | **0.35** | NA | NA | NA | NA | NA | NA | NA | NA | **2.49** | **6.19** | **2.49** | **2.49** | 1 | NA | NA | NA |
| 6 | HLA-DPB1 | 04:01 | 12832.89 | **12754.33** | **-0.28** | **0.03** | 12770.87 | -0.37 | 0.05 | 12792.81 | -0.37 | 0.06 | 12756.08 | -0.30 | 0.05 | -0.55 | 0.07 | **0.76** | **0.57** | 0.69 | 0.69 | 1 | 0.69 | 0.74 | 0.58 |
| 7 | HLA-C | 04:01 | 12832.89 | **12796.15** | **0.32** | **0.05** | 12797.41 | 0.34 | 0.05 | 12828.87 | 0.51 | 0.20 | 12798.04 | 0.32 | 0.06 | 0.57 | 0.20 | **1.37** | **1.88** | 1.40 | 1.40 | 1 | 1.67 | 1.38 | 1.77 |
| 8 | HLA-DPB1 | 10:01 | 12832.89 | **12788.74** | **0.68** | **0.10** | 12792.28 | 0.66 | 0.10 | 12821.08 | 13.66 | 145.23 | 12784.71 | 0.63 | 0.10 | 13.69 | 145.23 | **1.97** | **3.87** | 1.94 | 1.94 | 1 | 854569.26 | 1.88 | 879427.77 |
| 9 | HLA-DPB1 | 17:01 | 12832.89 | 12779.41 | 0.88 | 0.11 | **12779.24** | **0.89** | **0.12** | 12834.32 | 1.09 | 1.41 | 12781.21 | 0.88 | 0.12 | 1.12 | 1.41 | 2.40 | 5.76 | **2.42** | **2.42** | 1 | 2.98 | 2.42 | 3.06 |
| 10^c^ | HLA-DPA | 02:01 | - | - | - | - | - | - | - | - | - | - | - | - | - | - | - | - | - | - | - | - | - | - | - |
