# Supplementary material for: Amino acid residues in five separate HLA genes can explain most of the known associations between the MHC and primary biliary cholangitis
Source: PLoS Genet. 2018 Dec 3;14(12):e1007833. doi: 10.1371/journal.pgen.1007833 (PMC6292650; doi:10.1371/journal.pgen.1007833)
Supplement: S11 Table — Entries corresponding to the preferred model (that with the lowest AIC) in each row are shown in bold. (DOCX) [file pgen.1007833.s011.docx]

**S11 Table:** Comparison of allelic (=multiplicative), dominant, recessive and genotypic models for top associated amino acid substitutions. Entries corresponding to the preferred model (that with the lowest AIC) in each row are shown in **bold**.

|  |  |  |  |  | | |  | | |  | | |  | | | | | Implied odds ratios (ORs) | | | | | | | |
| --- | --- | --- | --- | --- | --- | --- | --- | --- | --- | --- | --- | --- | --- | --- | --- | --- | --- | --- | --- | --- | --- | --- | --- | --- | --- |
| Gene | Peptide position | Residue |  | Allelic (multiplicative) model | | | Dominant model | | | Recessive model | | | Genotypic model | | | | | Allelic model | | Dominant model | | Recessive model | | Genotypic model | |
|  |  |  | Null AIC | AIC | lnOR | SE | AIC | lnOR | SE | AIC | lnOR | SE | AIC | lnOR1 | SE1 | lnOR2 | SE2 | OR1 | OR2 | OR1 | OR2 | OR1 | OR2 | OR1 | OR2 |
| HLA-DPB1 | 11 | L/G | 12832.89 | **12578.23** | **0.56** | **0.03** | 12603.25 | 0.66 | 0.04 | 12748.62 | 0.79 | 0.08 | 12578.35 | 0.60 | 0.05 | 1.05 | 0.09 | **1.75** | **3.05** | 1.94 | 1.94 | 1 | 2.20 | 1.82 | 2.85 |
| HLA-DRB1 | 74 | L | 12832.89 | 12629.77 | 1.11 | 0.08 | **12624.14** | **1.15** | **0.08** | 12833.33 | 0.75 | 0.59 | 12625.85 | 1.16 | 0.08 | 0.84 | 0.59 | 3.04 | 9.25 | **3.17** | **3.17** | 1 | 2.13 | 3.18 | 2.32 |
| HLA-DQB1 | 57 | D | 12832.89 | **12778.58** | **-0.23** | **0.03** | 12793.46 | -0.3 | 0.05 | 12801.46 | -0.31 | 0.06 | 12780.57 | -0.24 | 0.05 | -0.46 | 0.06 | **0.79** | **0.63** | 0.74 | 0.74 | 1 | 0.73 | 0.79 | 0.63 |
| HLA-C | 156 | R | 12832.89 | **12788.05** | **0.23** | **0.03** | 12789.42 | 0.29 | 0.04 | 12820.68 | 0.28 | 0.07 | 12788.31 | 0.27 | 0.05 | 0.4 | 0.08 | **1.25** | **1.57** | 1.34 | 1.34 | 1 | 1.32 | 1.31 | 1.50 |
| HLA-DQA1 | -13 | A | 12832.89 | **12712.32** | **0.68** | **0.07** | 12823.64 | 0.99 | 0.34 | 12713.02 | 0.72 | 0.07 | 12713.62 | 0.39 | 0.34 | 1.09 | 0.34 | **1.97** | **3.89** | 2.7 | 2.7 | 1 | 2.05 | 1.47 | 2.96 |
| HLA-B | 45 | T | 12832.89 | **12796.72** | **0.24** | **0.04** | 12802.42 | 0.26 | 0.05 | 12819.66 | 0.44 | 0.11 | 12798.59 | 0.23 | 0.05 | 0.51 | 0.11 | **1.27** | **1.61** | 1.30 | 1.30 | 1 | 1.55 | 1.26 | 1.66 |
| HLA-DQA1 | 207 | V | 12832.89 | **12769.98** | **0.33** | **0.04** | 12810.42 | 0.65 | 0.14 | 12778.72 | 0.36 | 0.05 | 12771.51 | 0.43 | 0.15 | 0.74 | 0.14 | **1.39** | **1.95** | 1.92 | 1.92 | 1 | 1.43 | 1.53 | 2.10 |
| HLA-DPB1 | 84  215 | V  T | 12832.89 | **12823.77** | **0.54** | **0.16** | **12823.77** | **0.54** | **0.16** | NA | NA | NA | NA | NA | NA | NA | NA | **1.71** | **2.93** | **1.71** | **1.71** | 1 | NA | NA | NA |
| HLA-B | 9 | H | 12832.89 | **12815.28** | **0.17** | **0.04** | 12817.89 | 0.18 | 0.04 | 12827.75 | 0.28 | 0.10 | 12817.27 | 0.16 | 0.05 | 0.34 | 0.11 | **1.18** | **1.39** | 1.2 | 1.2 | 1 | 1.33 | 1.18 | 1.40 |
