# Supplementary figures and images for: Amino acid residues in five separate HLA genes can explain most of the known associations between the MHC and primary biliary cholangitis
Source: PLoS Genet. 2018 Dec 3;14(12):e1007833. doi: 10.1371/journal.pgen.1007833 (PMC6292650; doi:10.1371/journal.pgen.1007833)

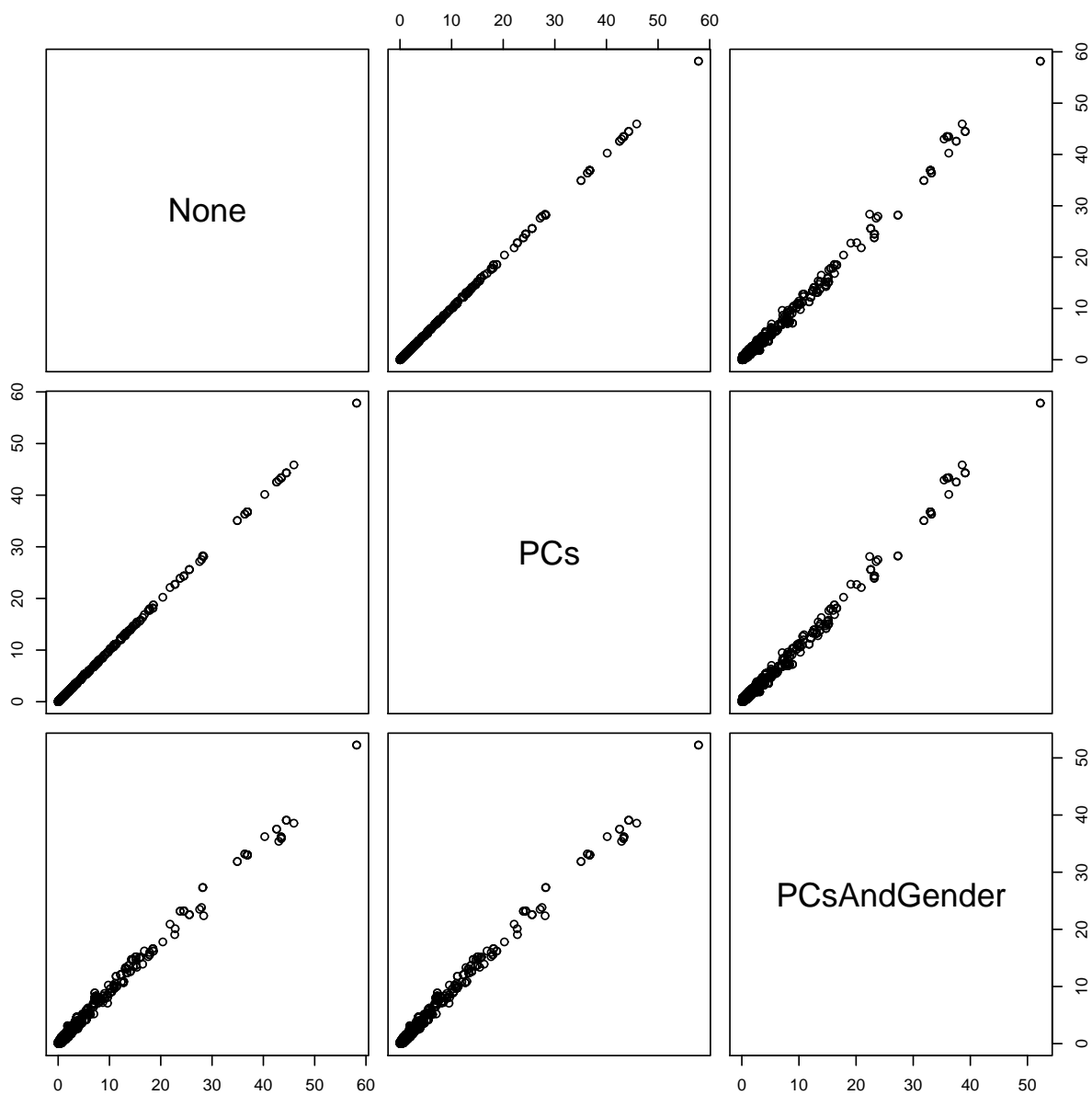

Supplement: S1 Fig — None: no covariates included; PCs: the top 10 principal component scores included as covariates; PCsAndGender: the top 10 principal component scores and gender included as covariates. (PDF) [file pgen.1007833.s012.pdf]

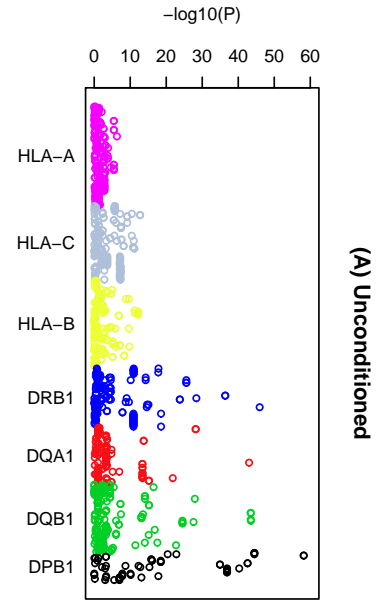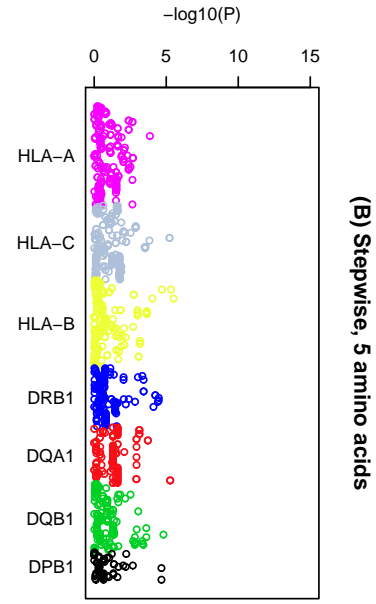

**(C) FINEMAP (4 amino acids)**

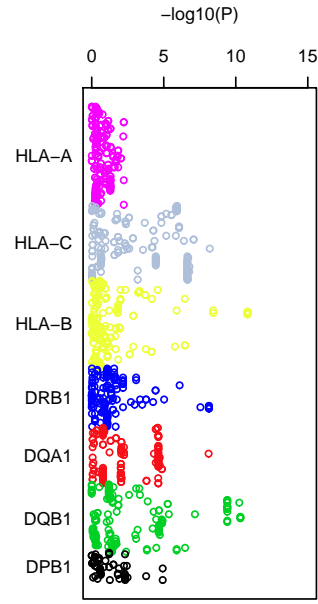

**(D) FINEMAP (5 amino acids)**

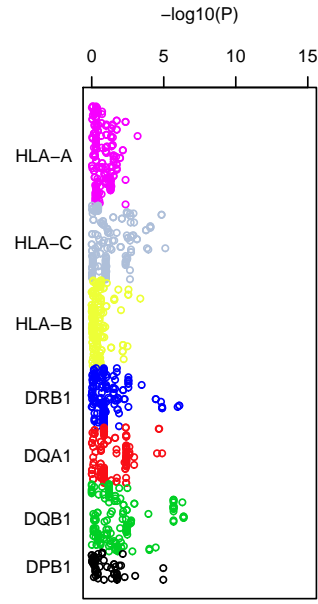

**(E) FINEMAP (6 amino acids)**

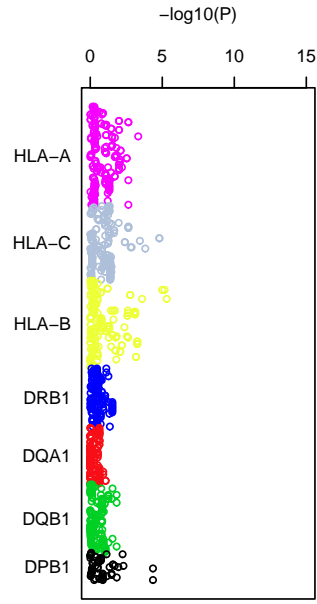

**(F) FINEMAP (7 amino acids)**

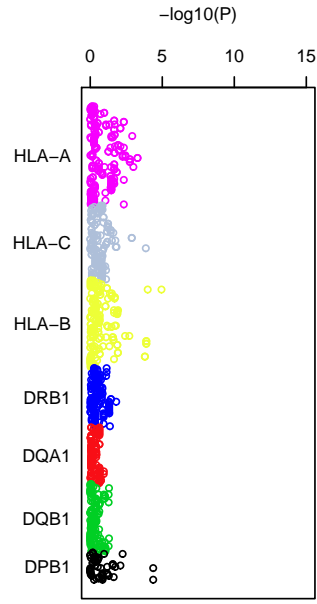

Supplement: S6 Fig — Association analysis results for individual amino acids while including in the regression model: (A) no other variables; (B) the top five amino acids from stepwise regression; (C) the four amino acids in the top model from FINEMAP, when limiting to a maximum of four predictors; (D) the five amino acids in the top model from FINEMAP, when limiting to a maximum of five predictors; (E) the six amino acids in the top model from FINEMAP, when limiting to a maximum of six predictors; (F) the seven amino acids in the top model from FINEMAP, when limiting to a maximum of seven predictors. (PDF) [file pgen.1007833.s017.pdf]

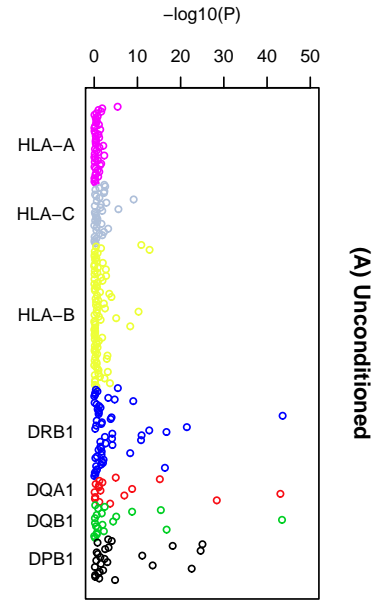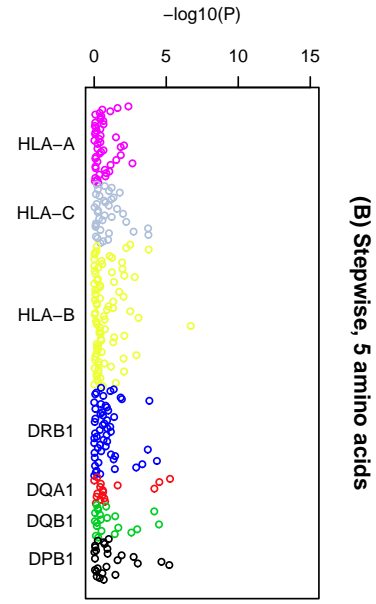

**(C) FINEMAP (4 amino acids)**

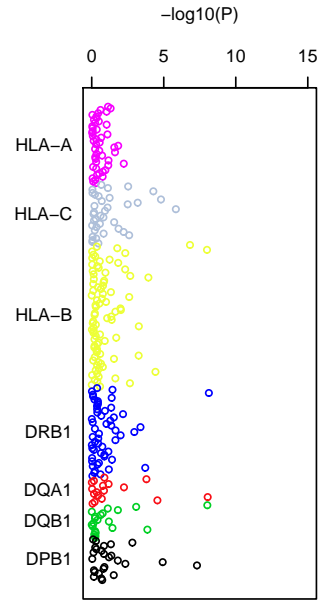

**(D) FINEMAP (5 amino acids)**

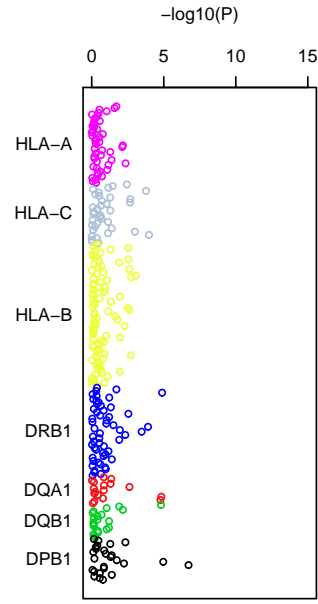

**(E) FINEMAP (6 amino acids)**

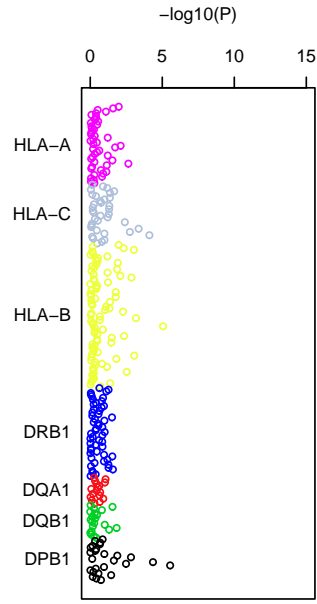

**(F) FINEMAP (7 amino acids)**

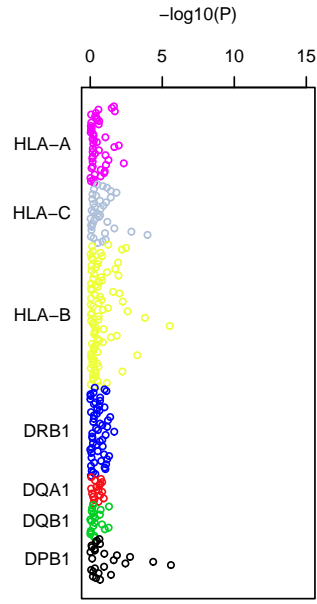

Supplement: S8 Fig — Association analysis results for individual classical alleles while including in the regression model: (A) no other variables; (B) the top five amino acids from stepwise regression; (C) the four amino acids in the top model from FINEMAP, when limiting to a maximum of four predictors; (D) the five amino acids in the top model from FINEMAP, when limiting to a maximum of five predictors; (E) the six amino acids in the top model from FINEMAP, when limiting to a maximum of six predictors; (F) the seven amino acids in the top model from FINEMAP, when limiting to a maximum of seven predictors. (PDF) [file pgen.1007833.s019.pdf]

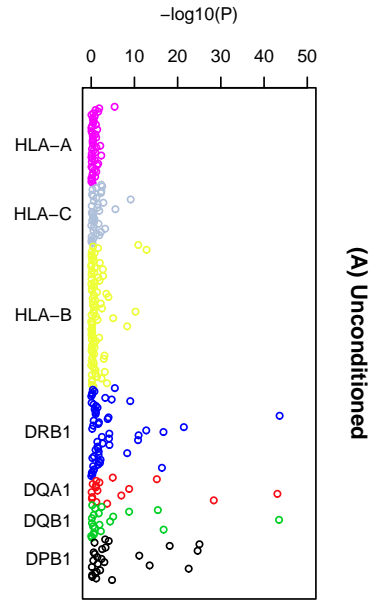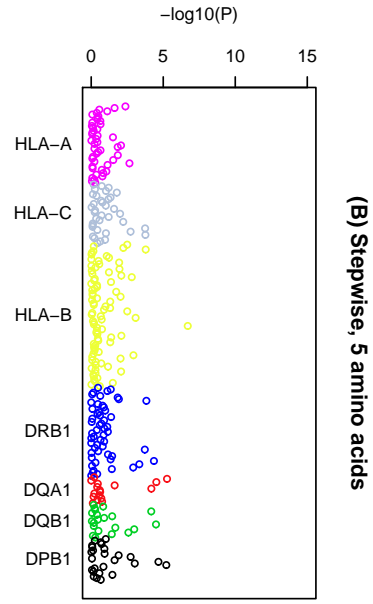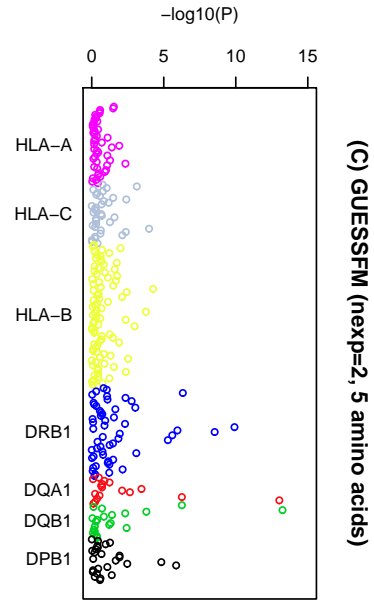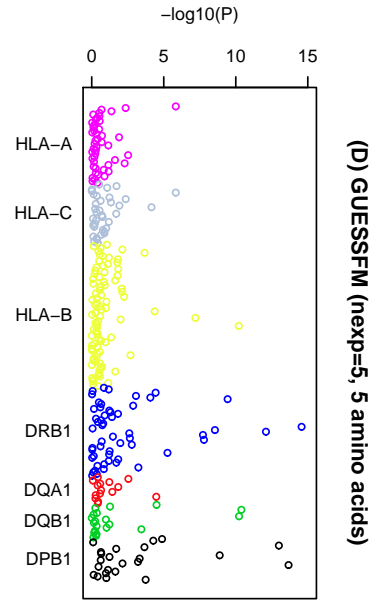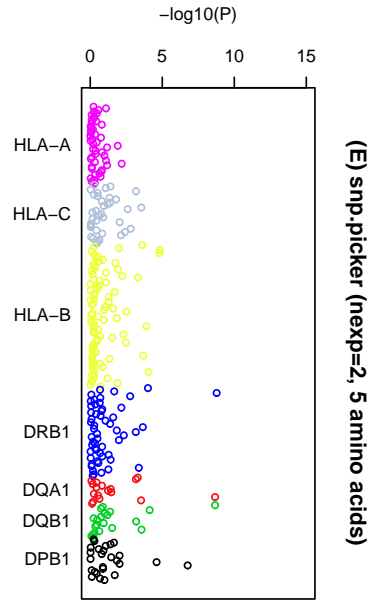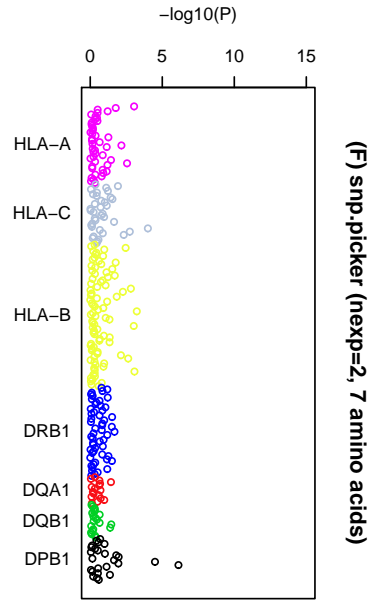

Supplement: S11 Fig — Association analysis results for individual classical alleles while including in the regression model: (A) no other variables; (B) the top five amino acids from stepwise regression; (C) the five amino acids with the highest posterior probabilities from GUESSFM when run with with nexp = 2; (D) the five amino acids with the highest posterior probabilities from GUESSFM when run with with nexp = 5; (E) the five amino acids with the highest posterior probabilities from snp.picker, applied following a GUESSFM run with nexp = 2; (F) the seven amino acids with the highest posterior probabilities from snp.picker, applied following a GUESSFM run with nexp = 2. (PDF) [file pgen.1007833.s022.pdf]

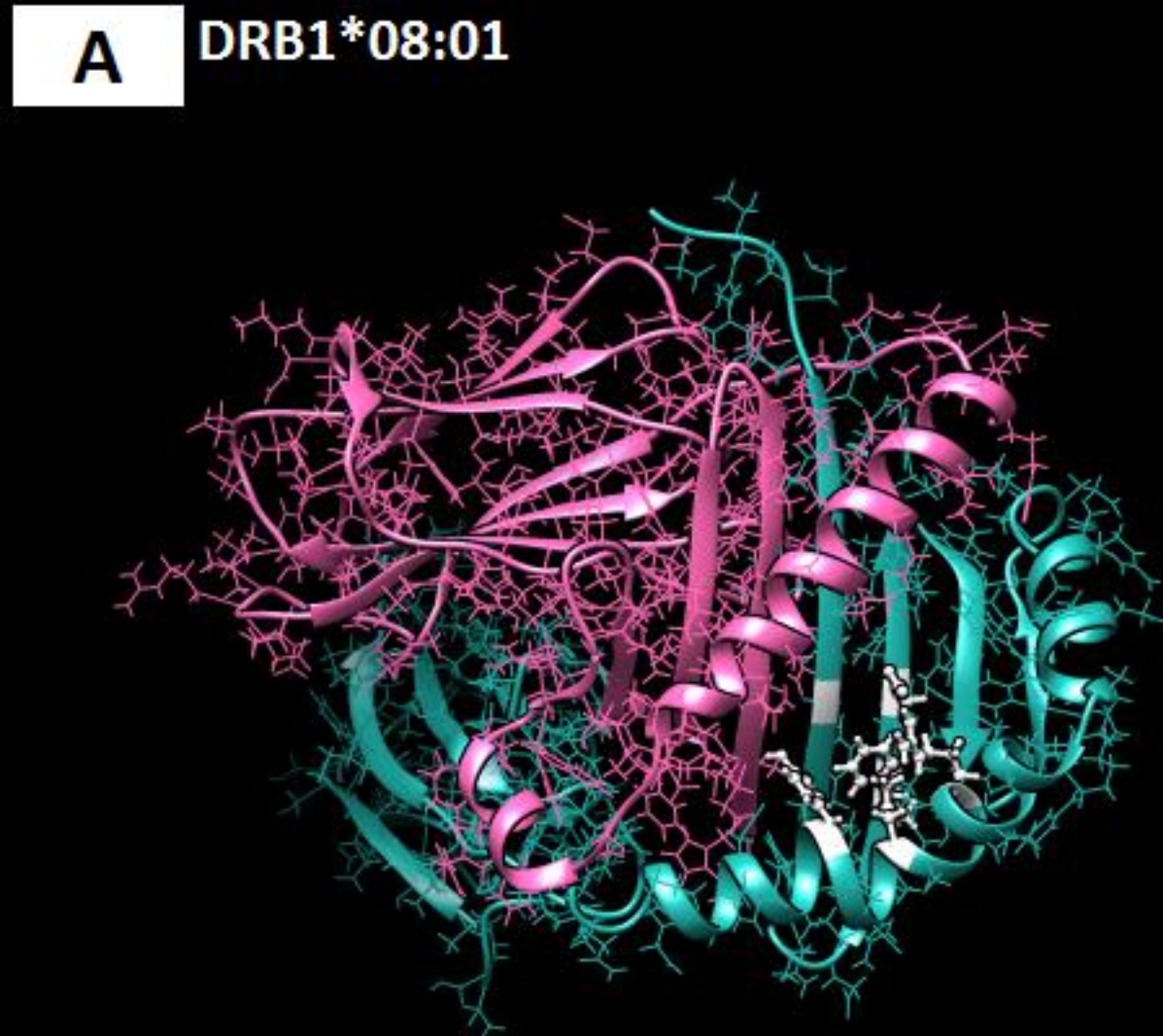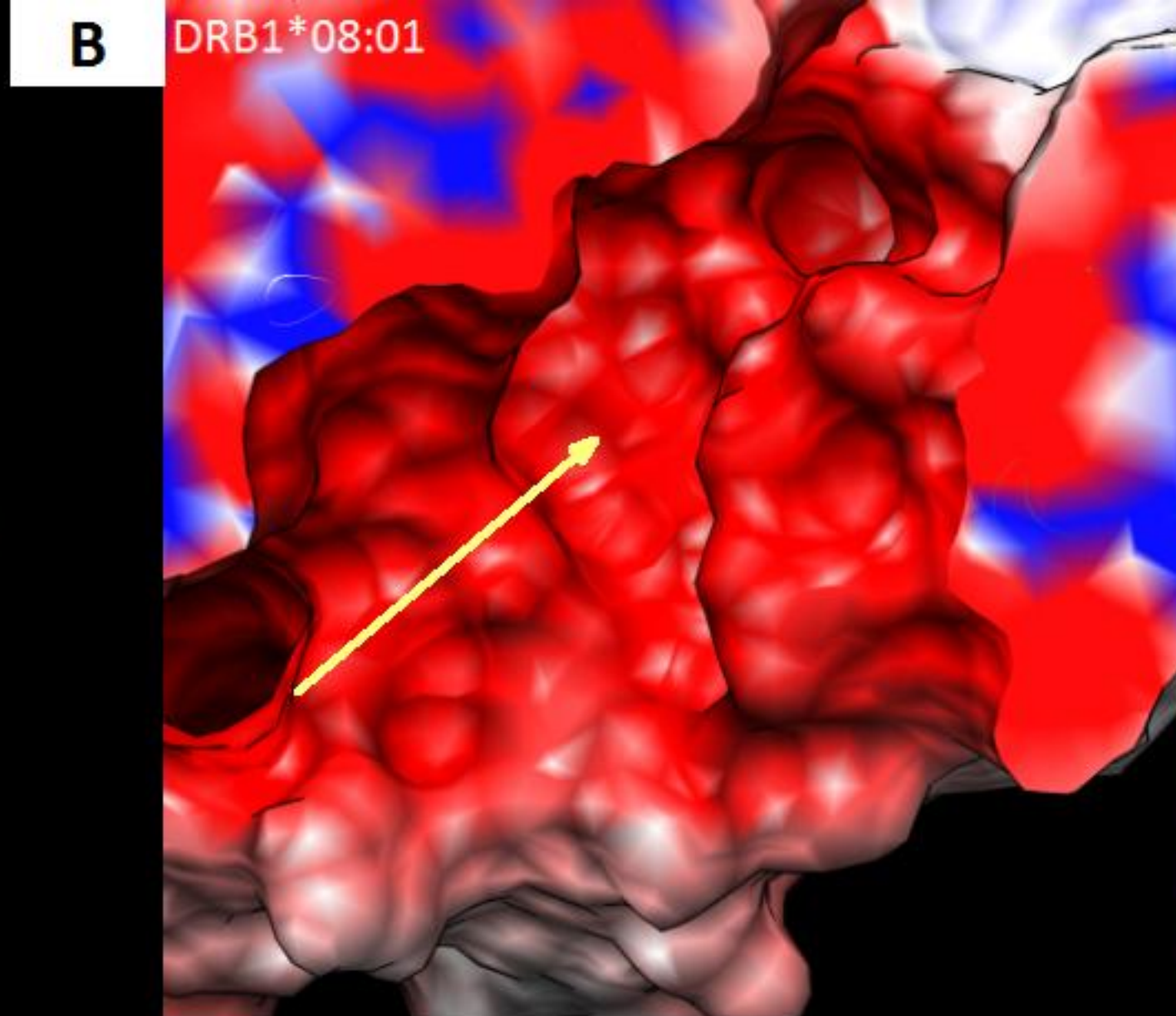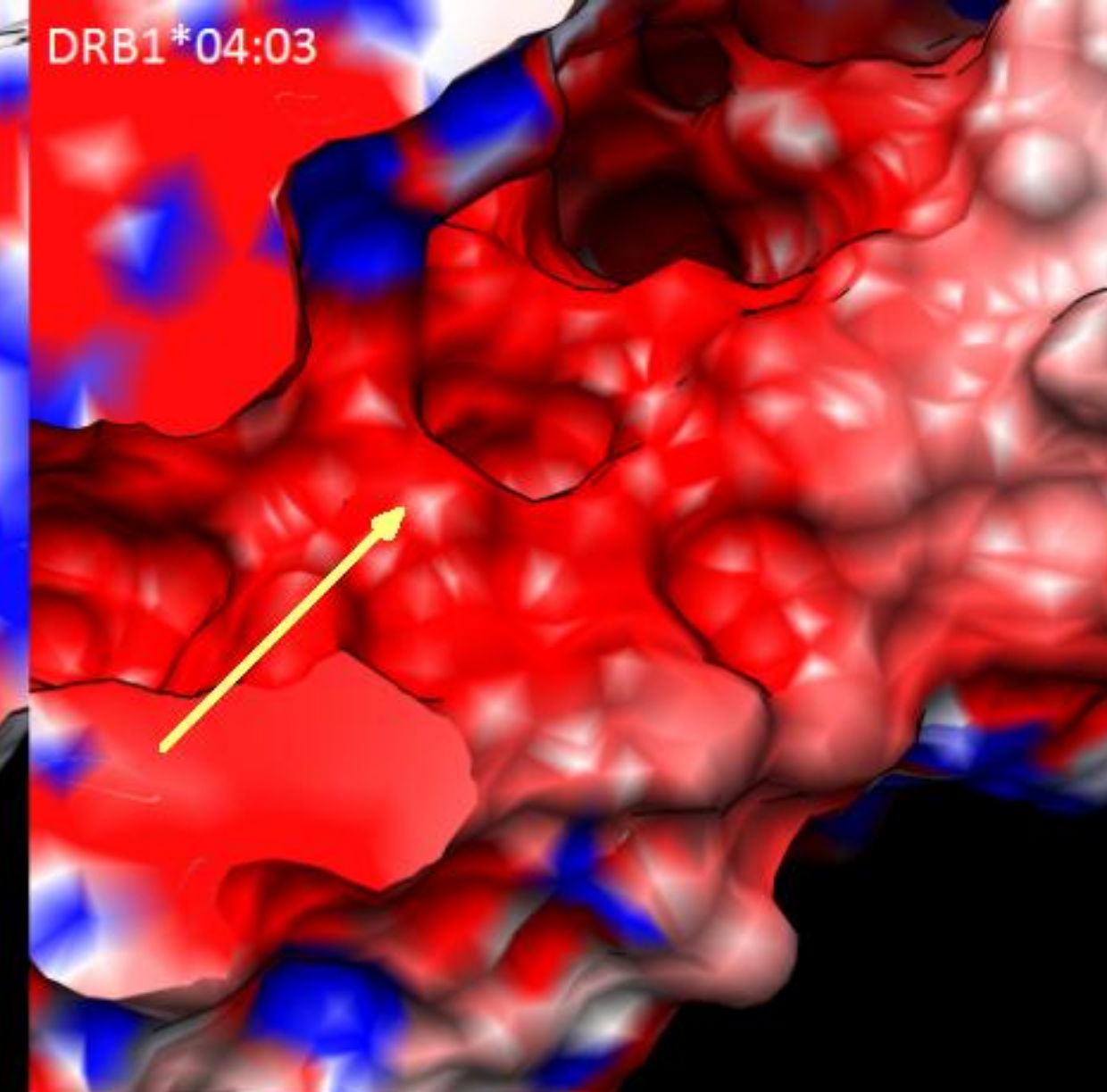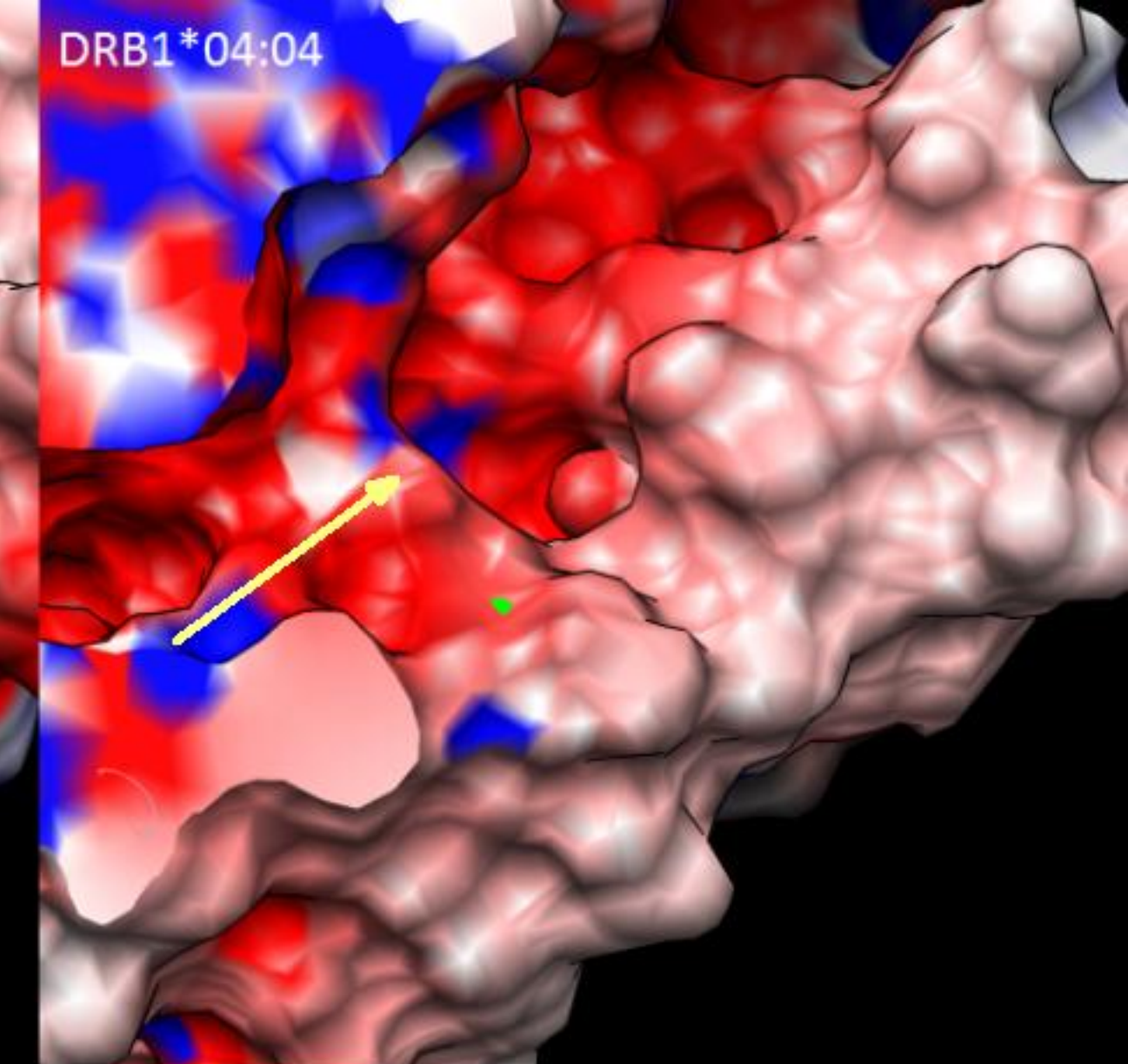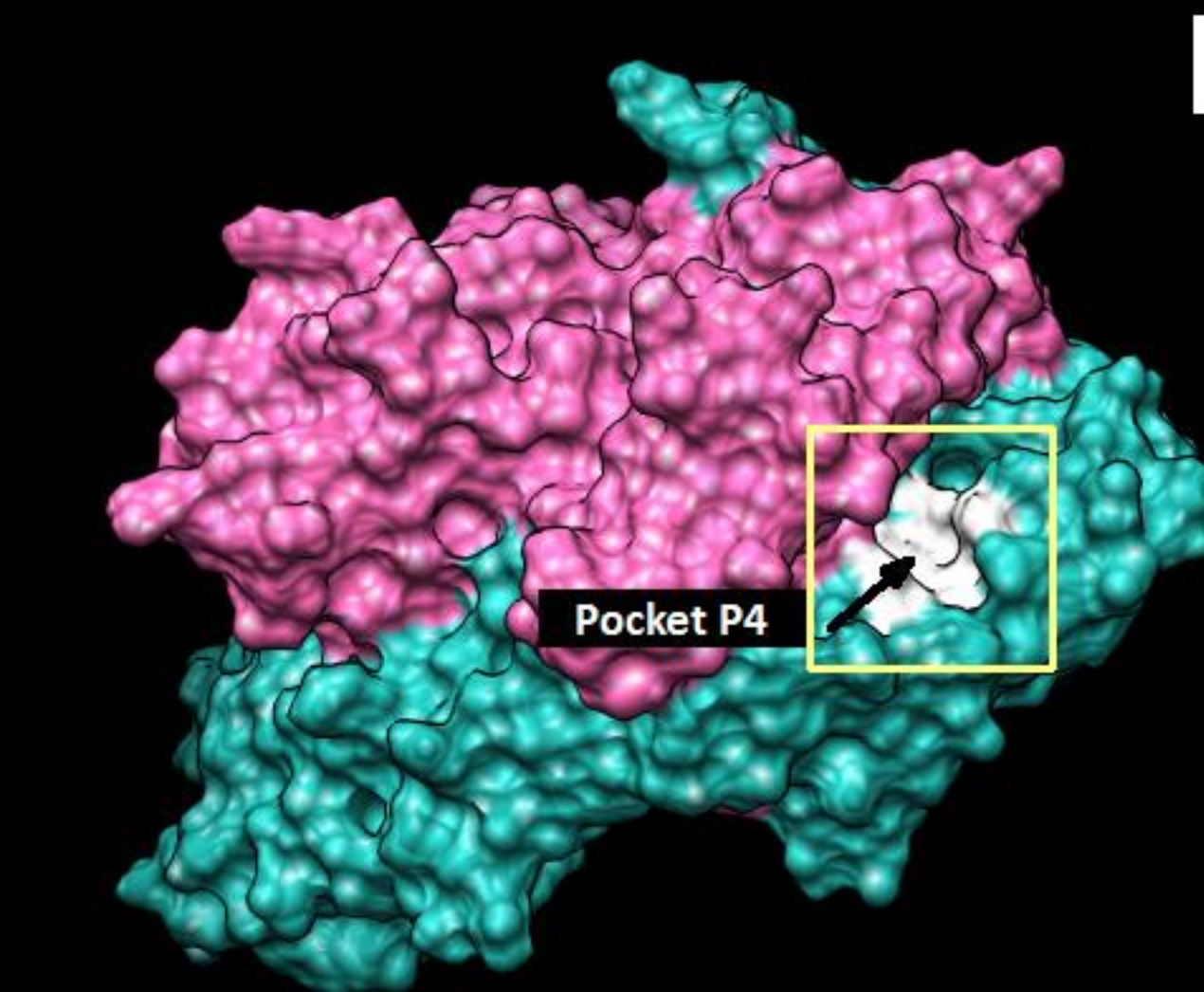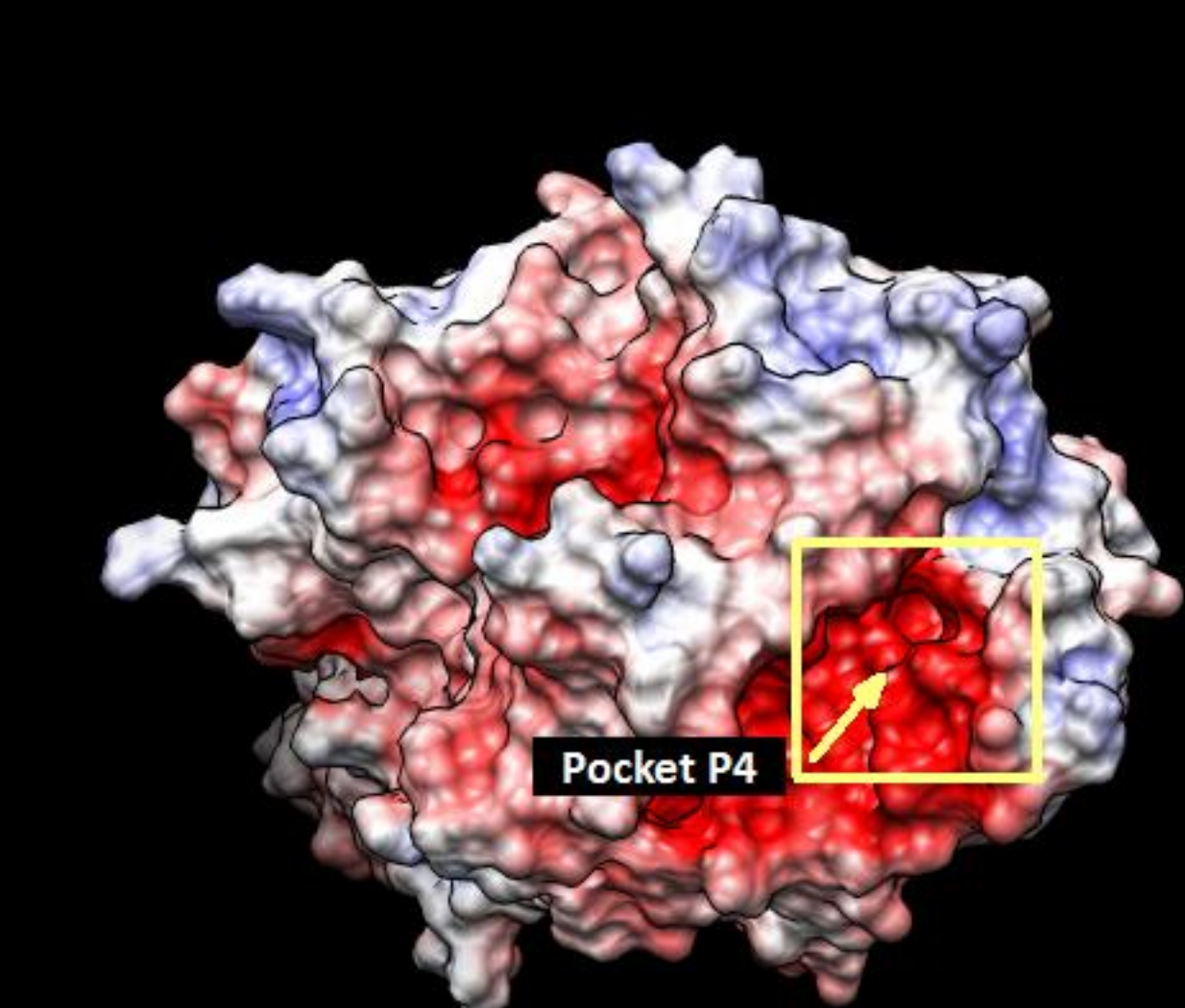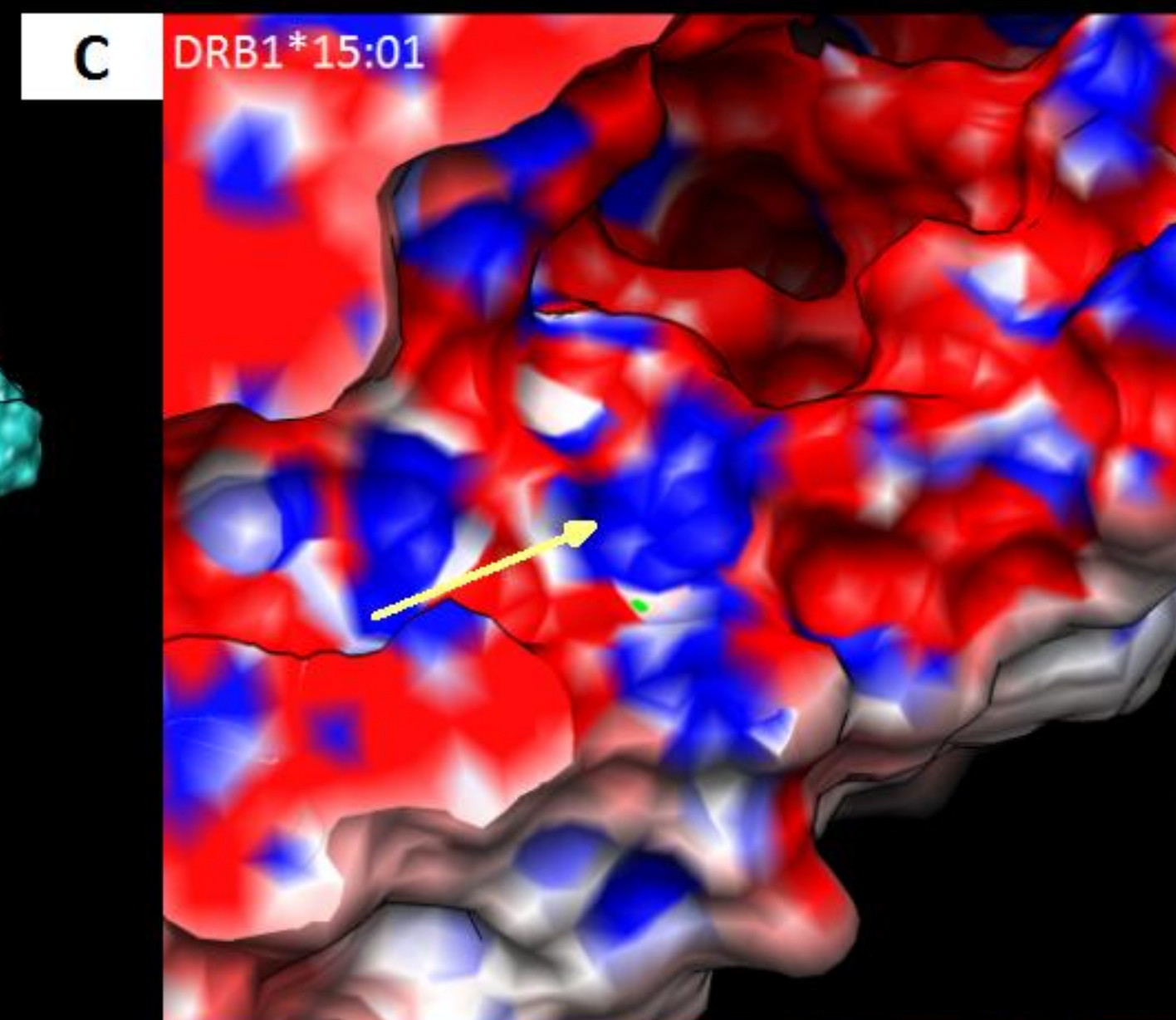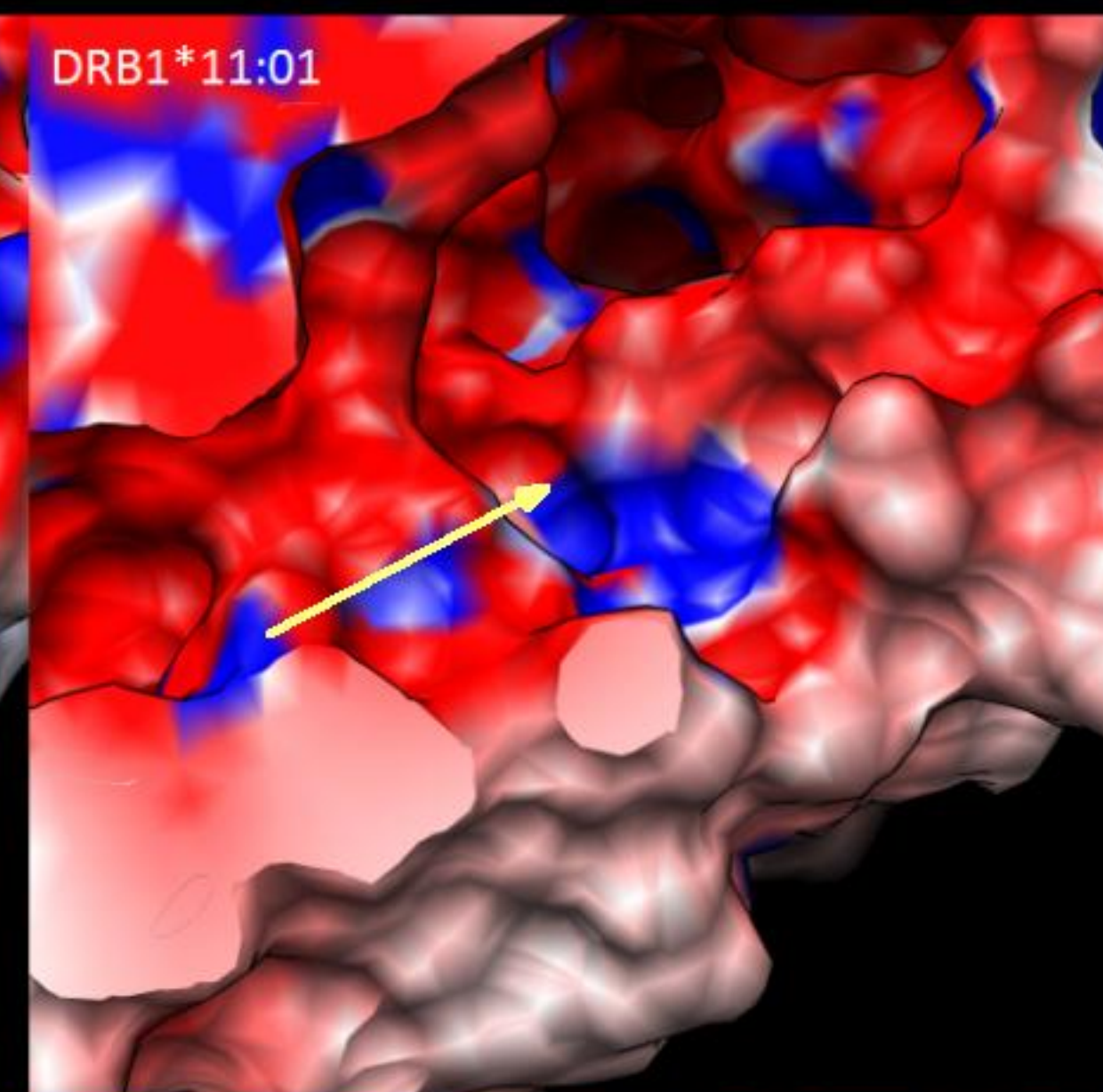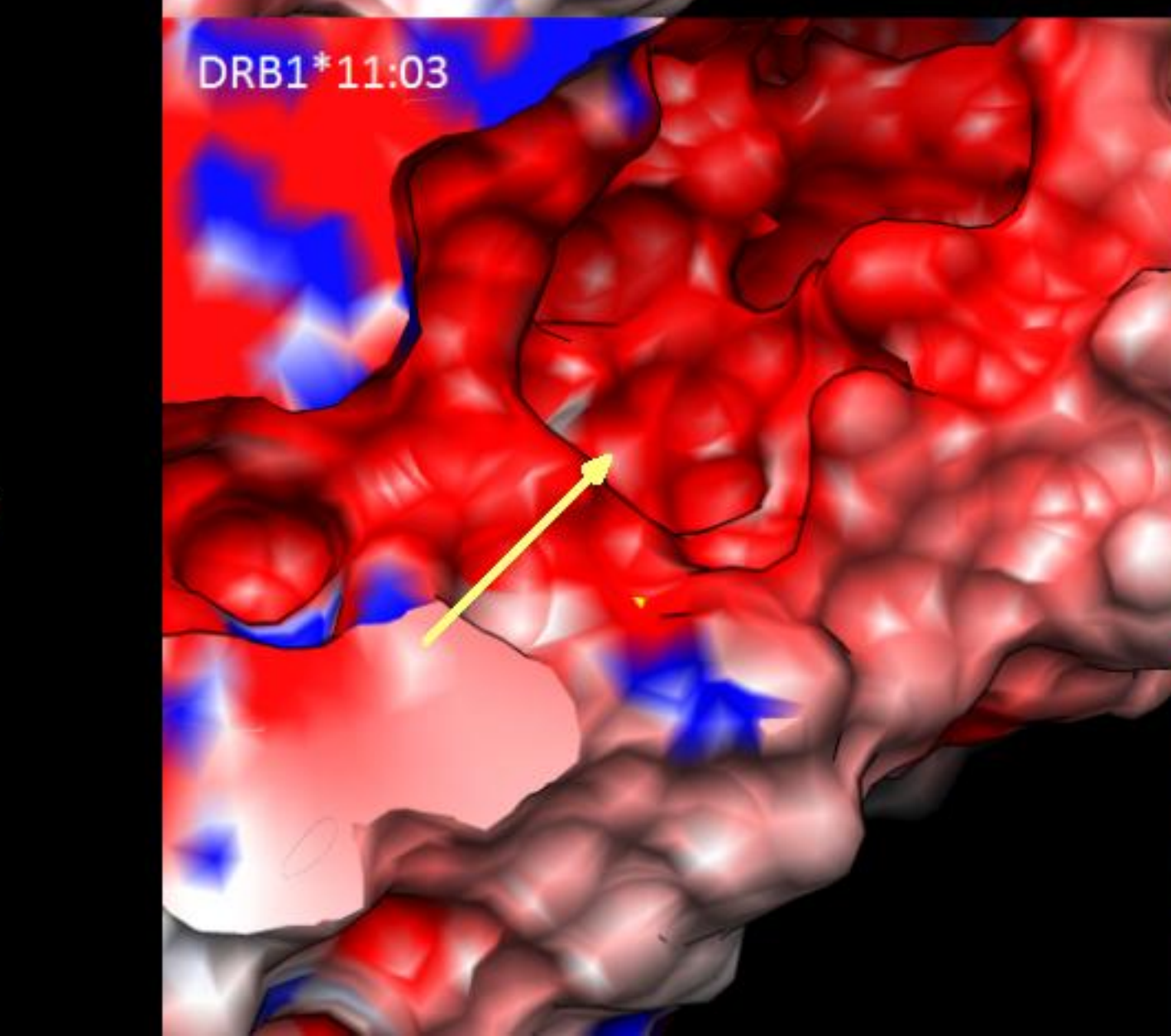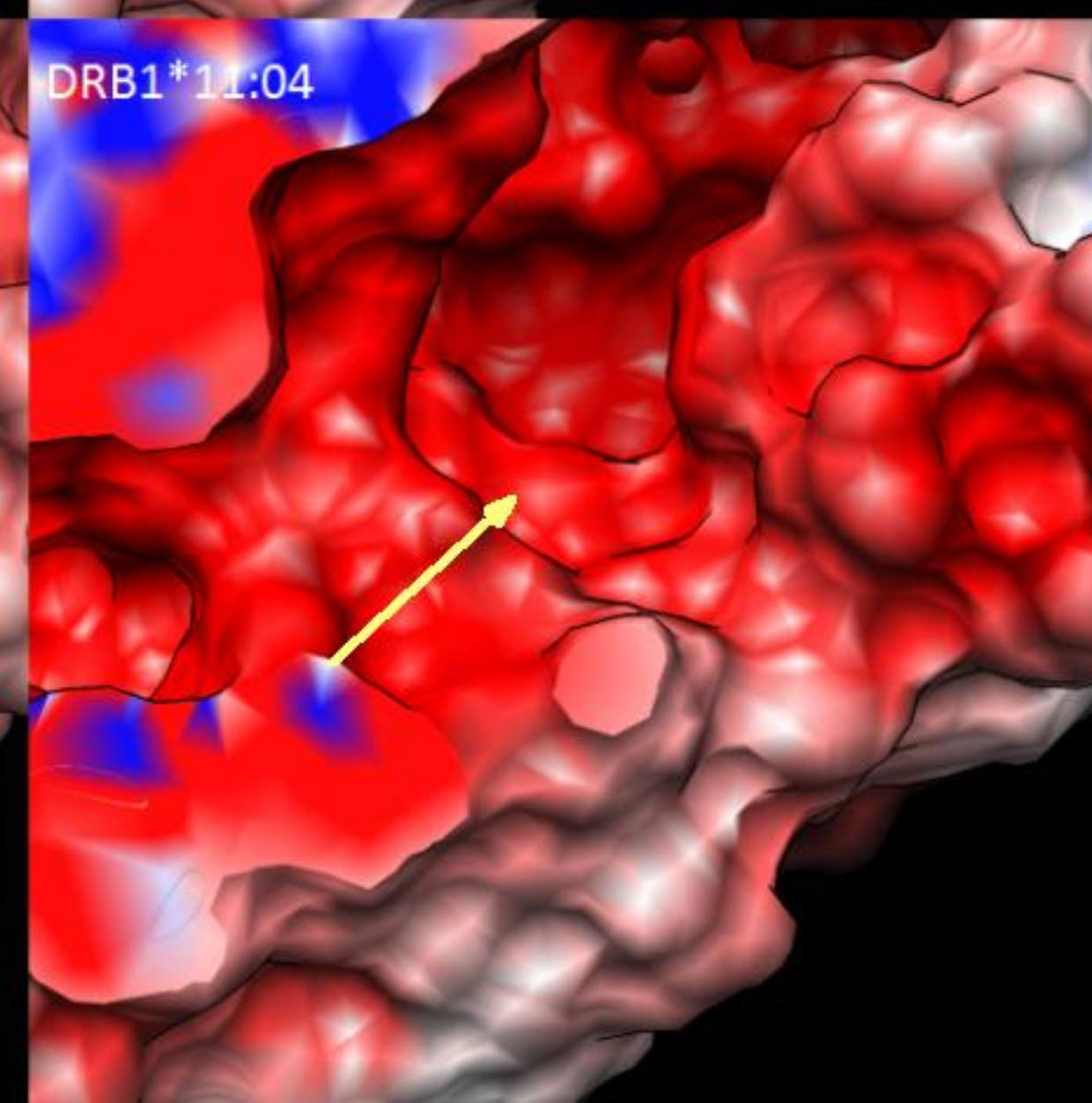

Supplement: S12 Fig — A) The structure and electrostatic potential of HLA-DRB1*08:01. The area within the frame is depicted in expanded form in B and C, and indicates the position of pocket P4 (arrows). All structures were superimposed on HLA-DRB1*08:01 and therefore show the same view. HLA-DRB1 alleles associated with an increased risk of PBC (08:01, 04:03 and 04:04) are shown in panel B whereas those associated with a protective effect (15:01,11:01,11:03 and 11:04) are shown in panel C. Negatively charged potentials (less than 5 kT/e) are coloured red, positively charged (greater than 5 kT/e) blue, and neutral potentials (0 kT/e) are coloured white. Linear interpolation was used to produce the colour for surface potentials between these values. (PDF) [file pgen.1007833.s023.pdf]

**A**

DQB\*04:02

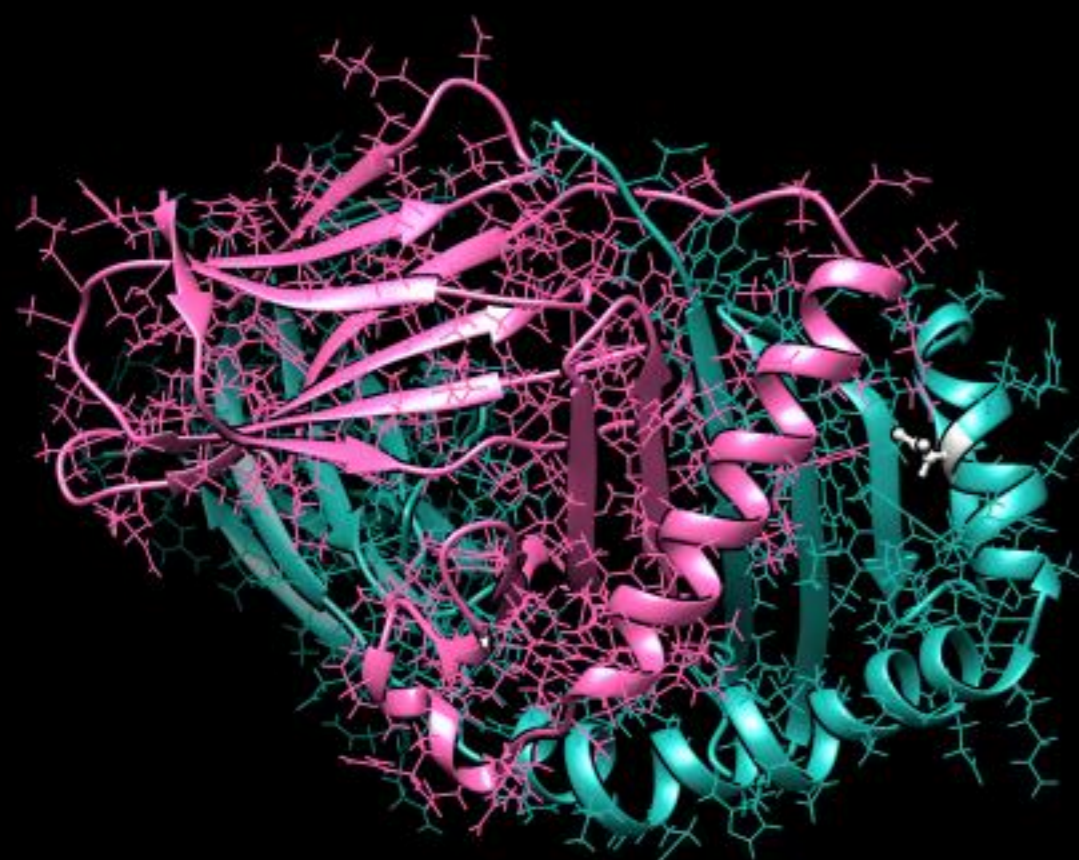**B**

DQB\*04:02

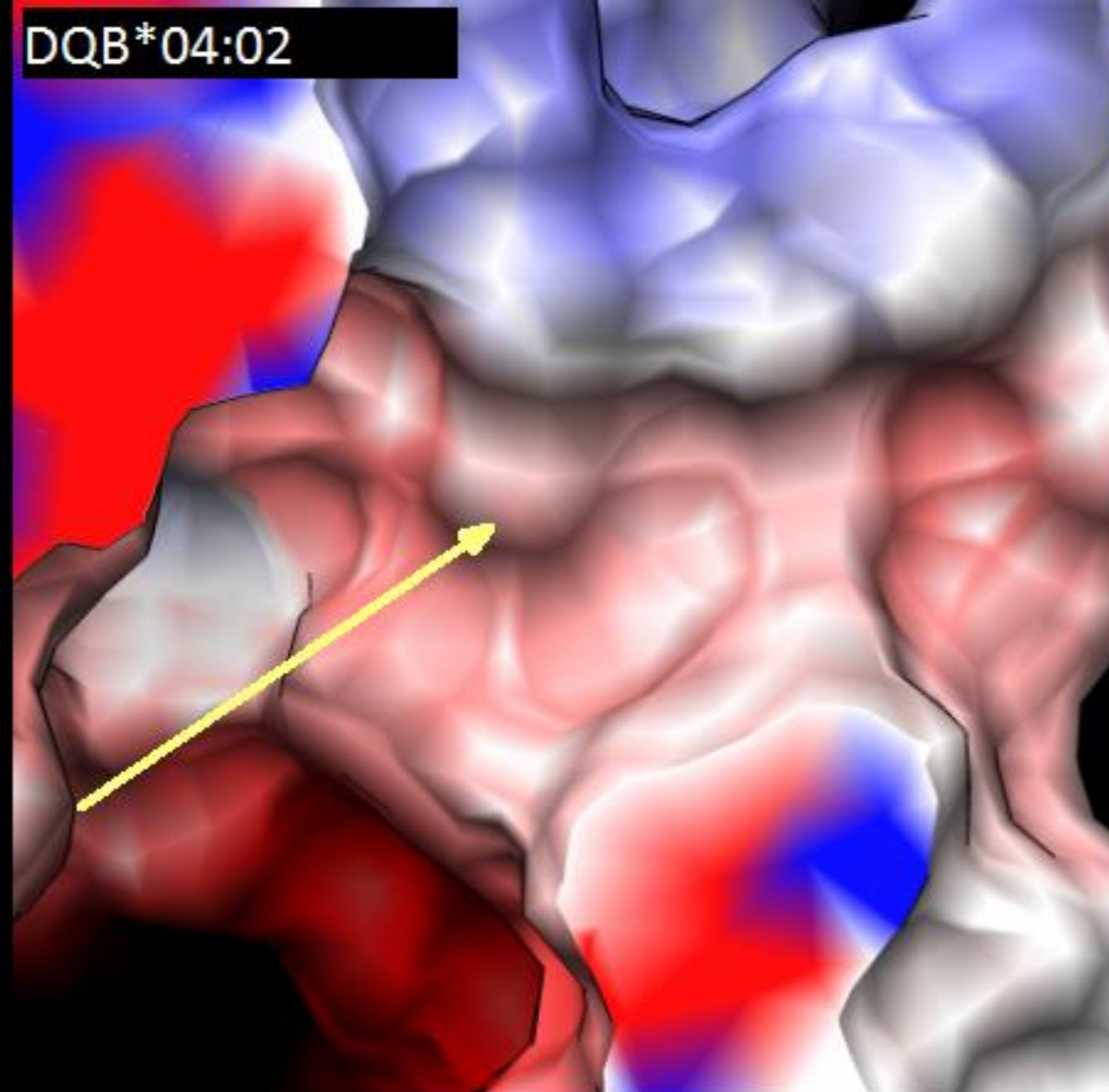

DQB\*03:02

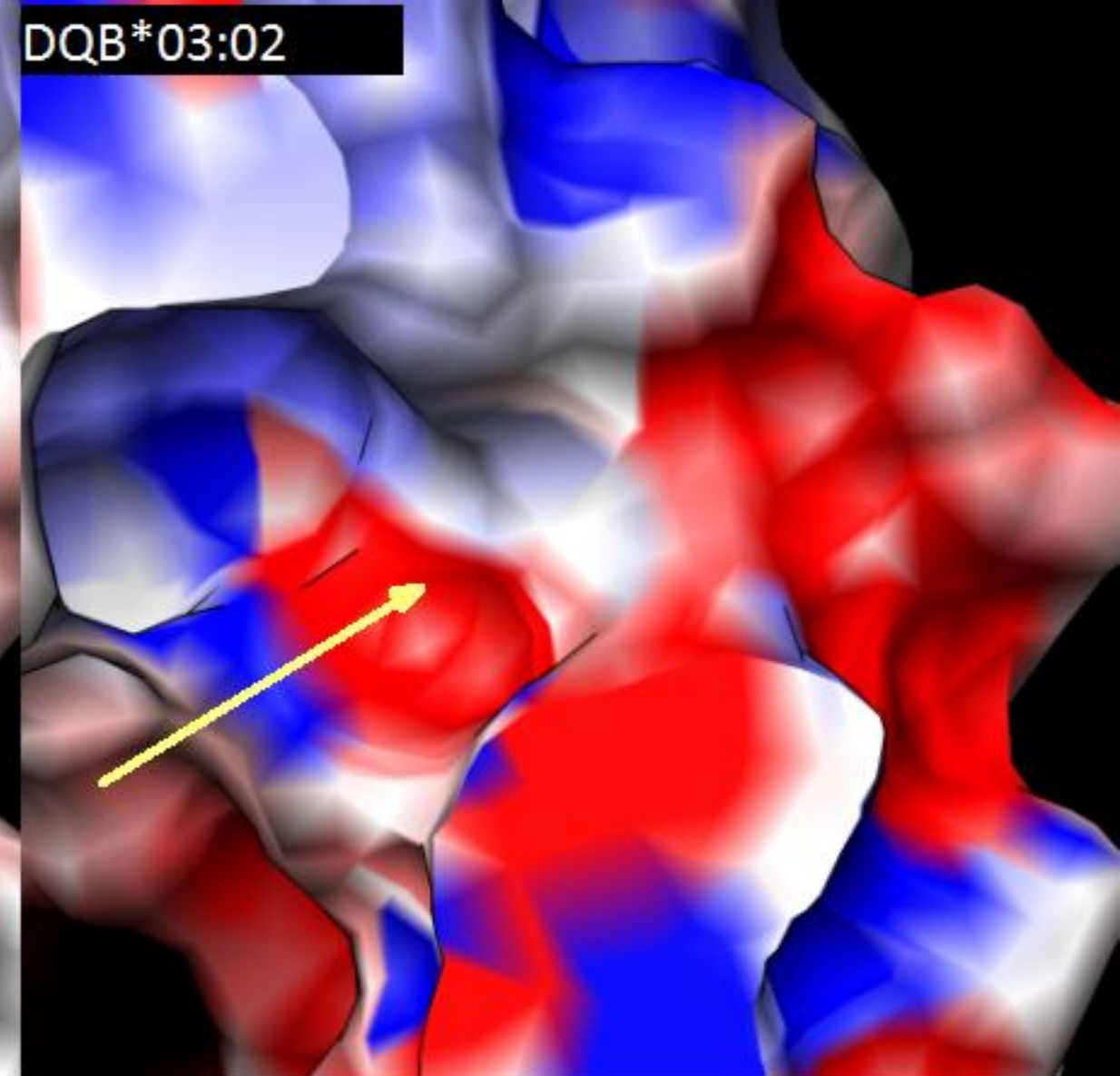**C**

DQB\*06:02

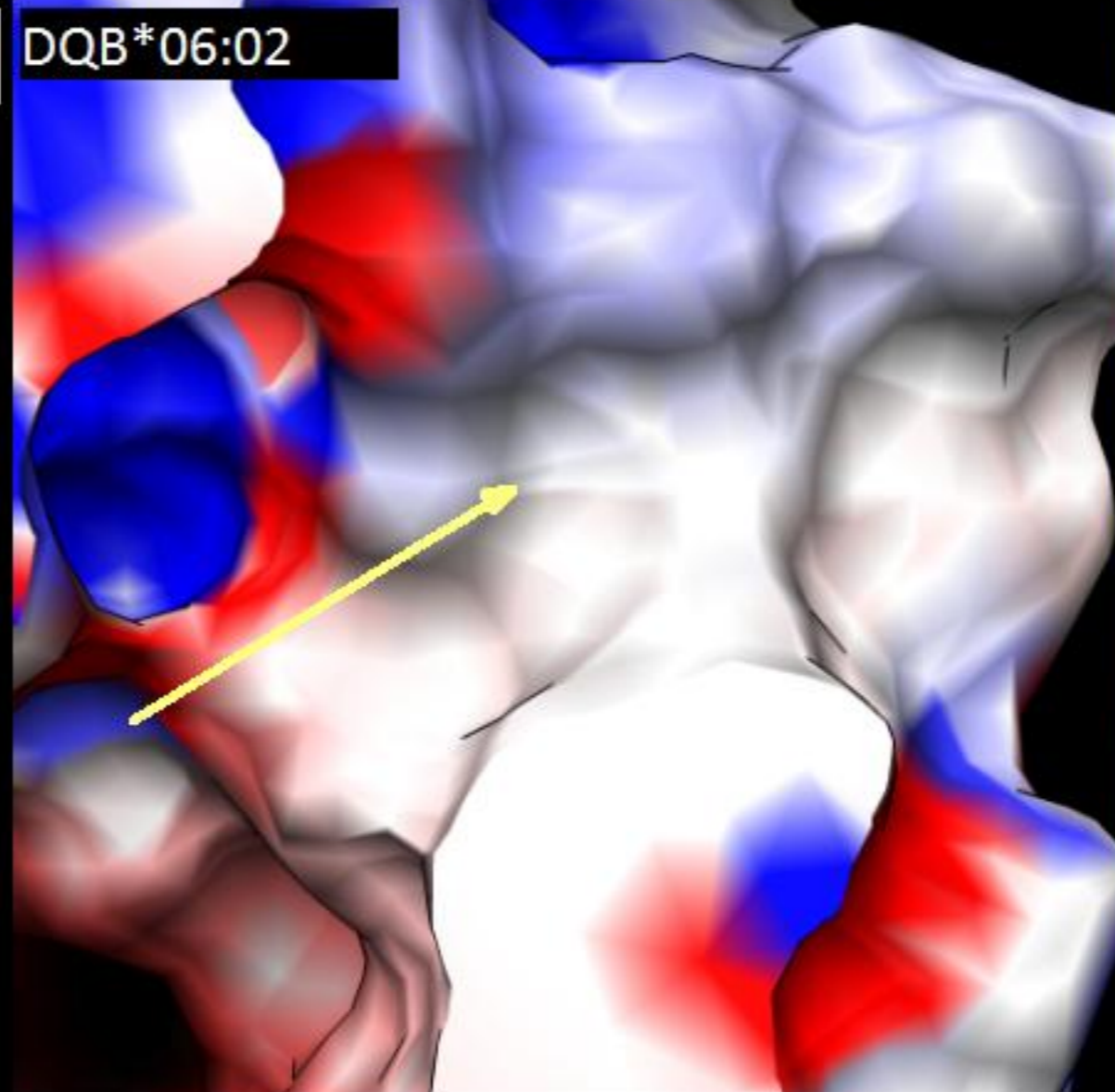

DQB\*03:01

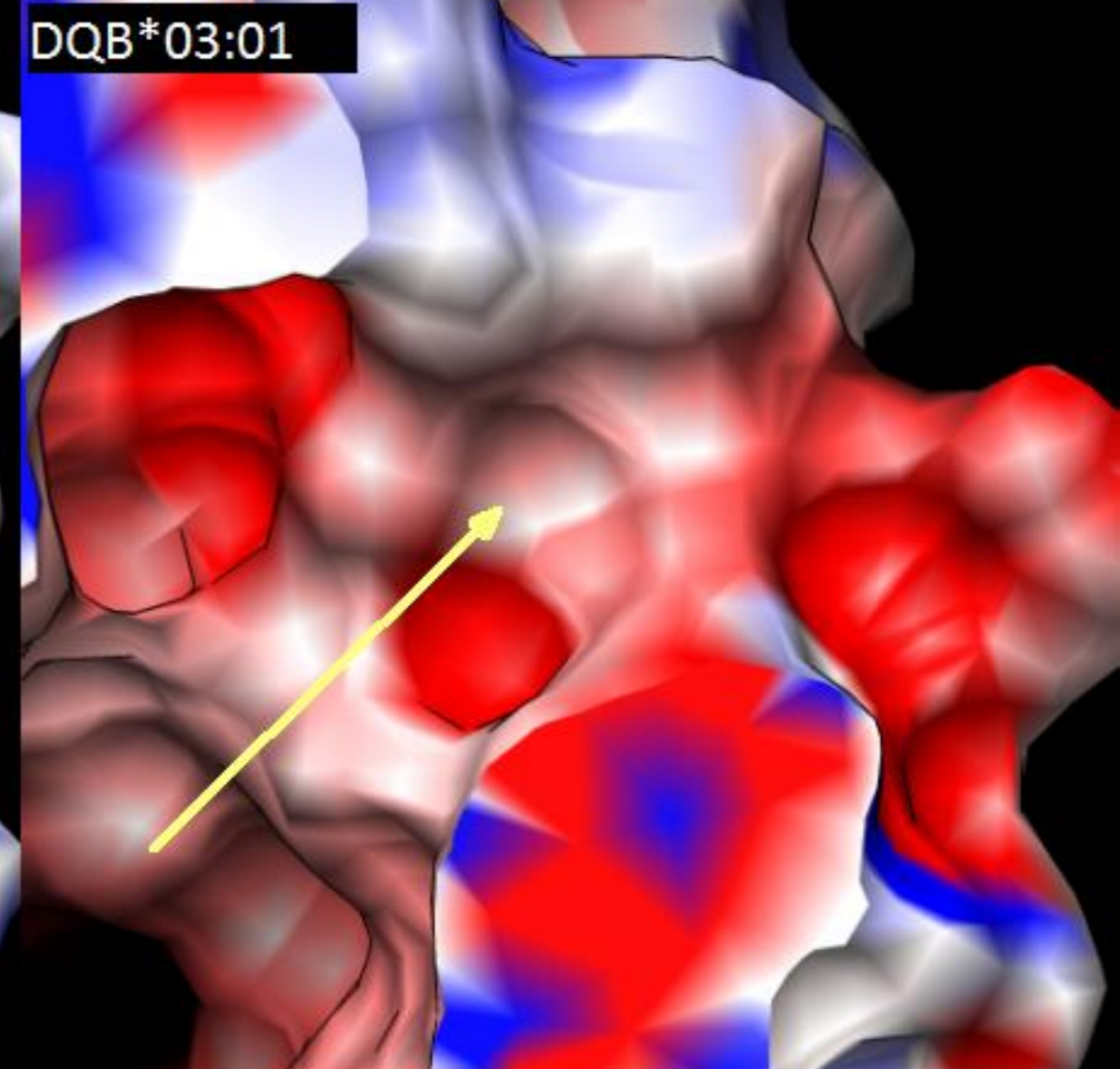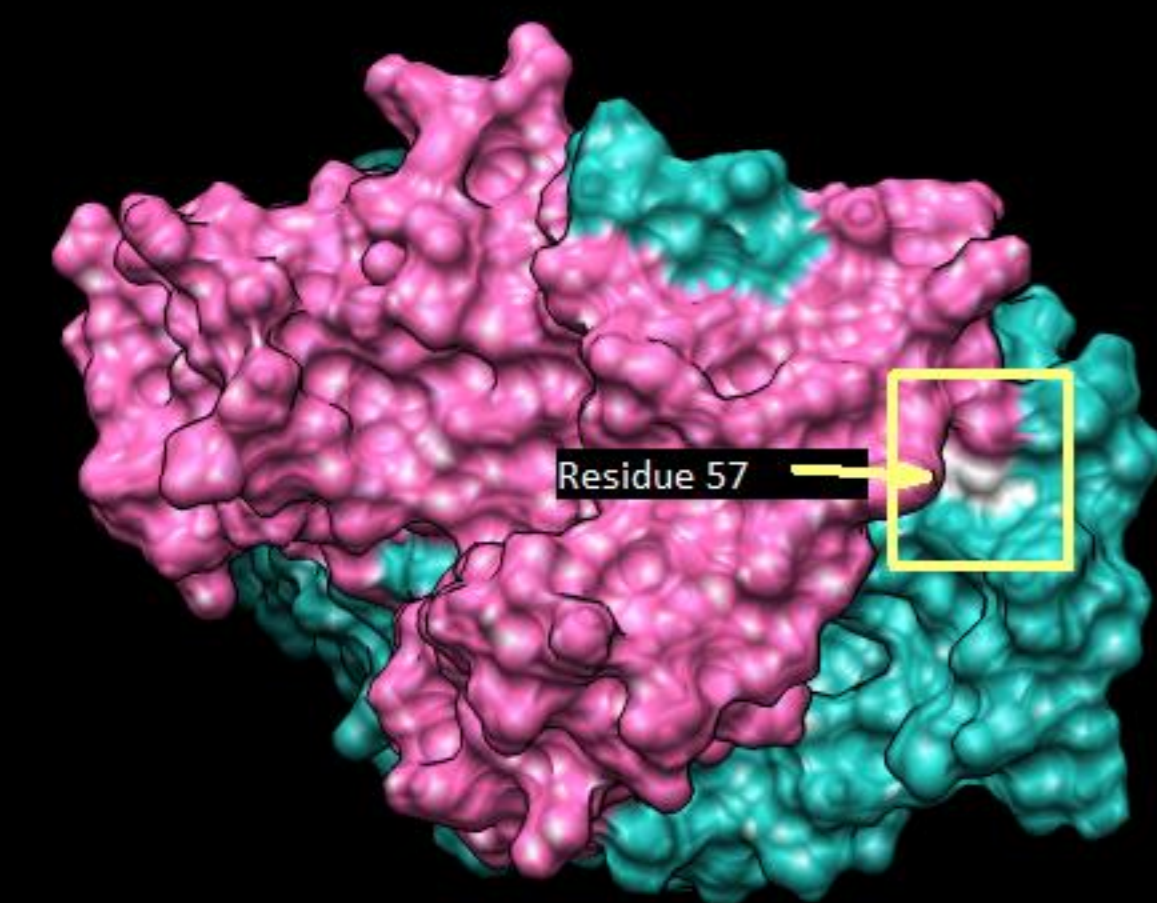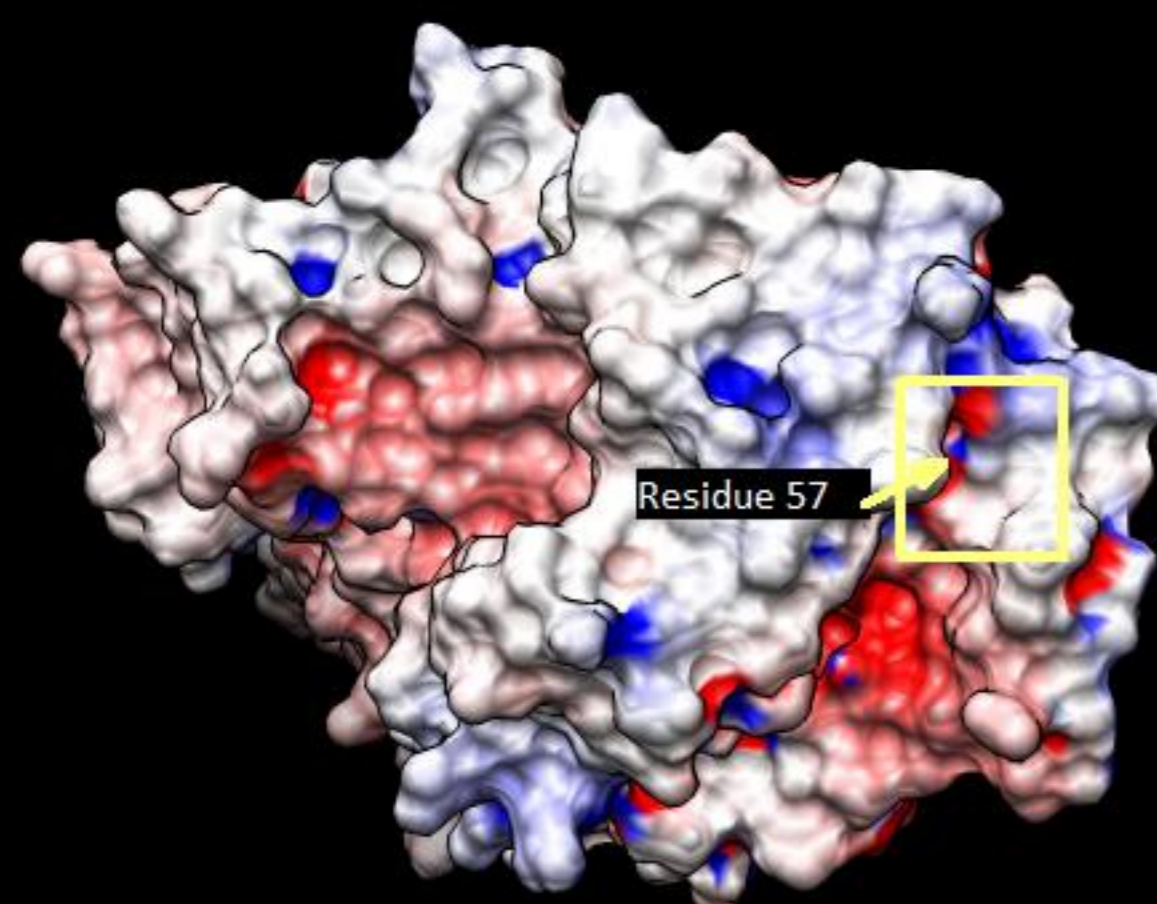

Supplement: S13 Fig — A) The structure and electrostatic potential of HLA-DQB1*04:02. The area within the frame is depicted in expanded form in B and C, and indicates the position of residue 57 (arrows). All structures were superimposed on HLA-DQB1*04:02 and therefore show the same view. HLA-DQB1 alleles associated with an increased risk of PBC (04:02 and 03:02) are shown in panel B whereas those associated with a protective effect (06:02 and 03:01) are shown in panel C. Negatively charged potentials (less than 5 kT/e) are coloured red, positively charged (greater than 5 kT/e) blue, and neutral potentials (0 kT/e) are coloured white. Linear interpolation was used to produce the colour for surface potentials between these values. (PDF) [file pgen.1007833.s024.pdf]

**A**

DQB\*04:02

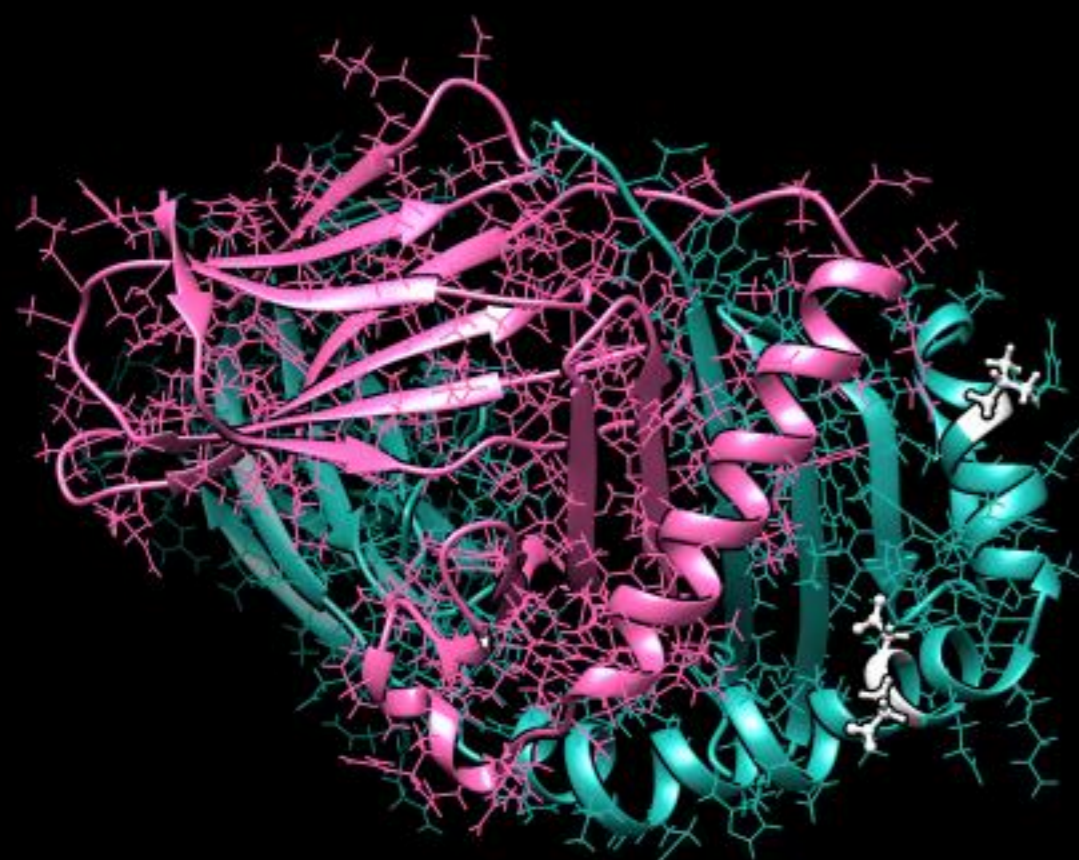**B**

DQB\*04:02

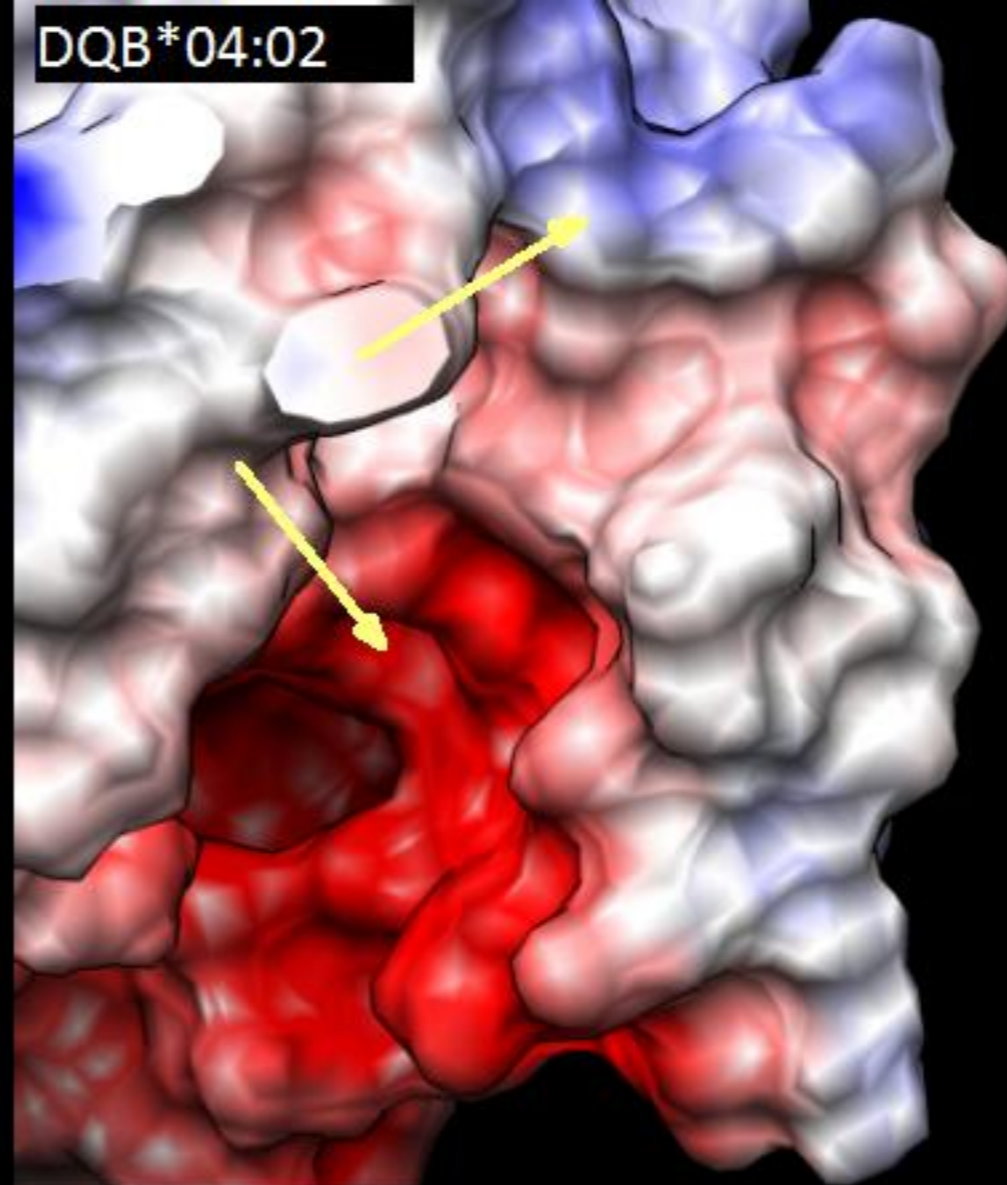

DQB\*03:02

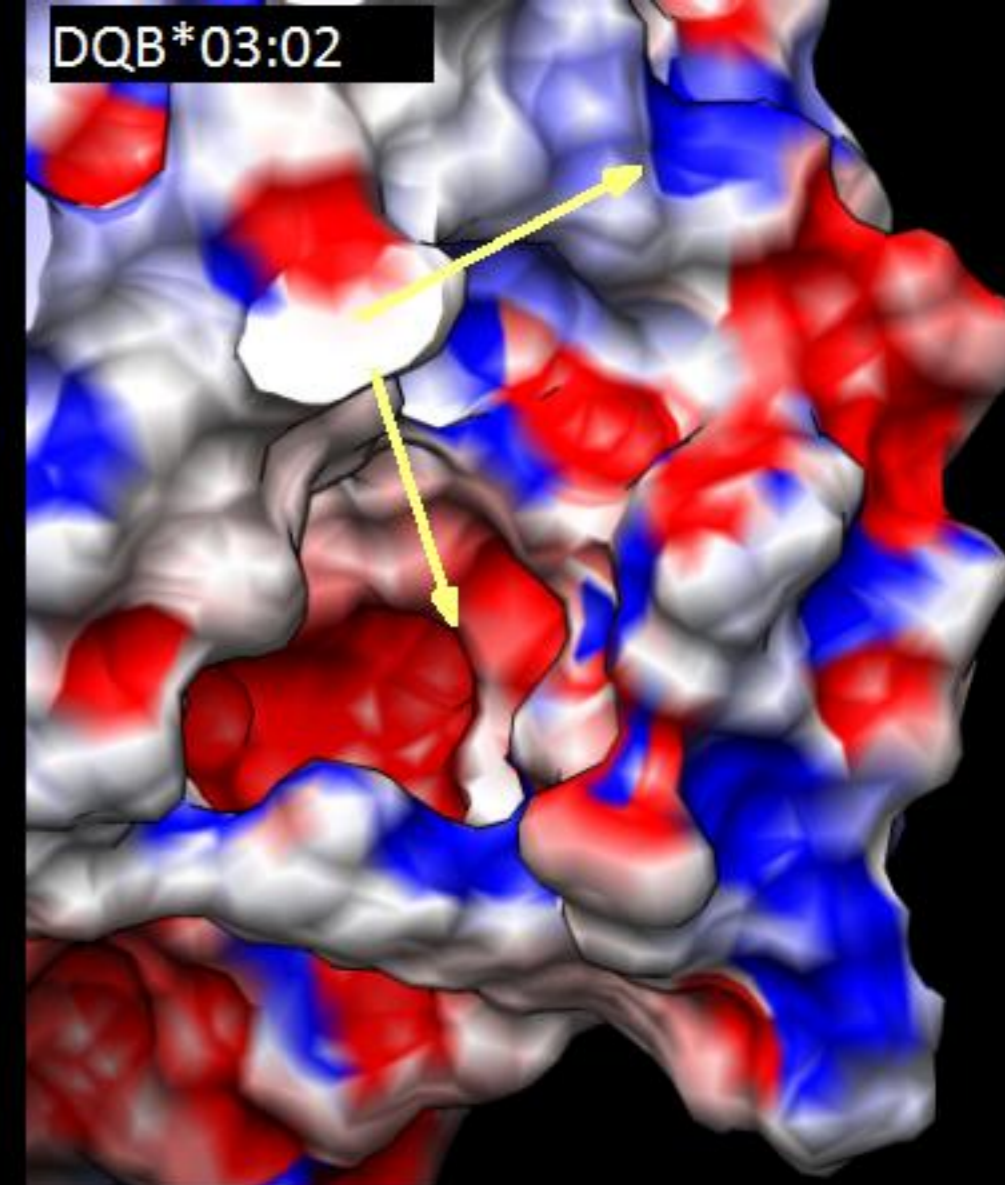**C**

DQB\*06:02

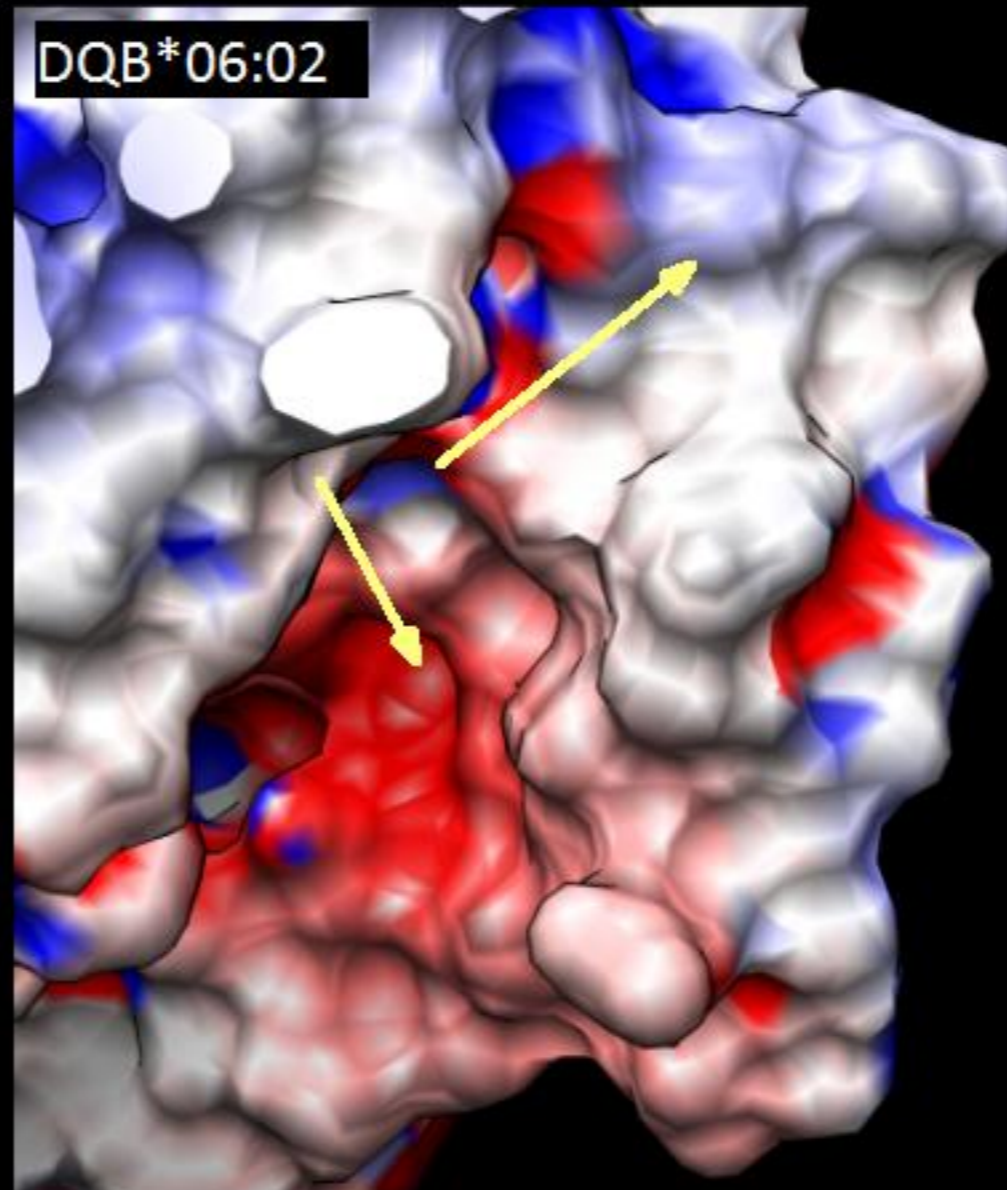

DQB\*03:01

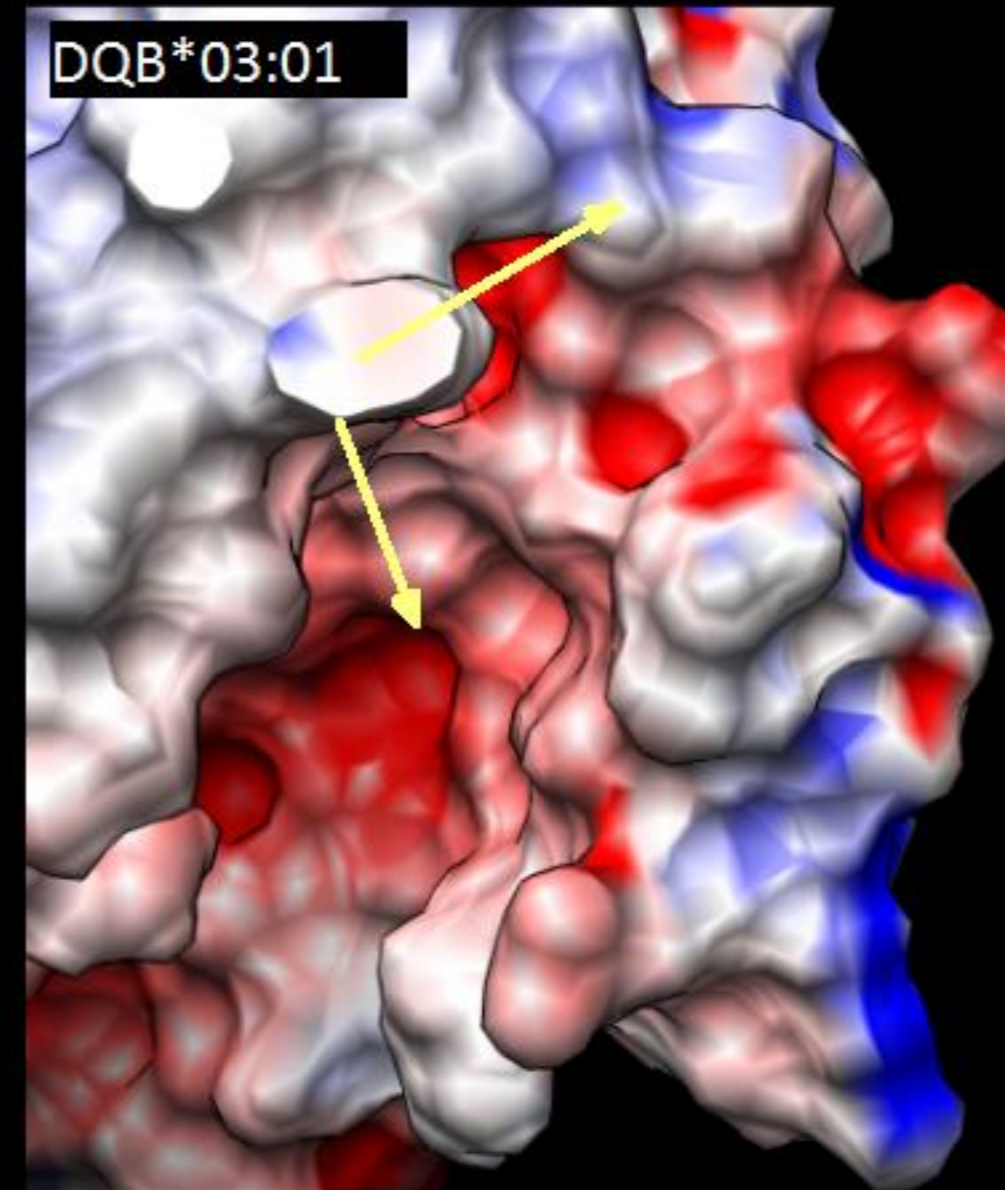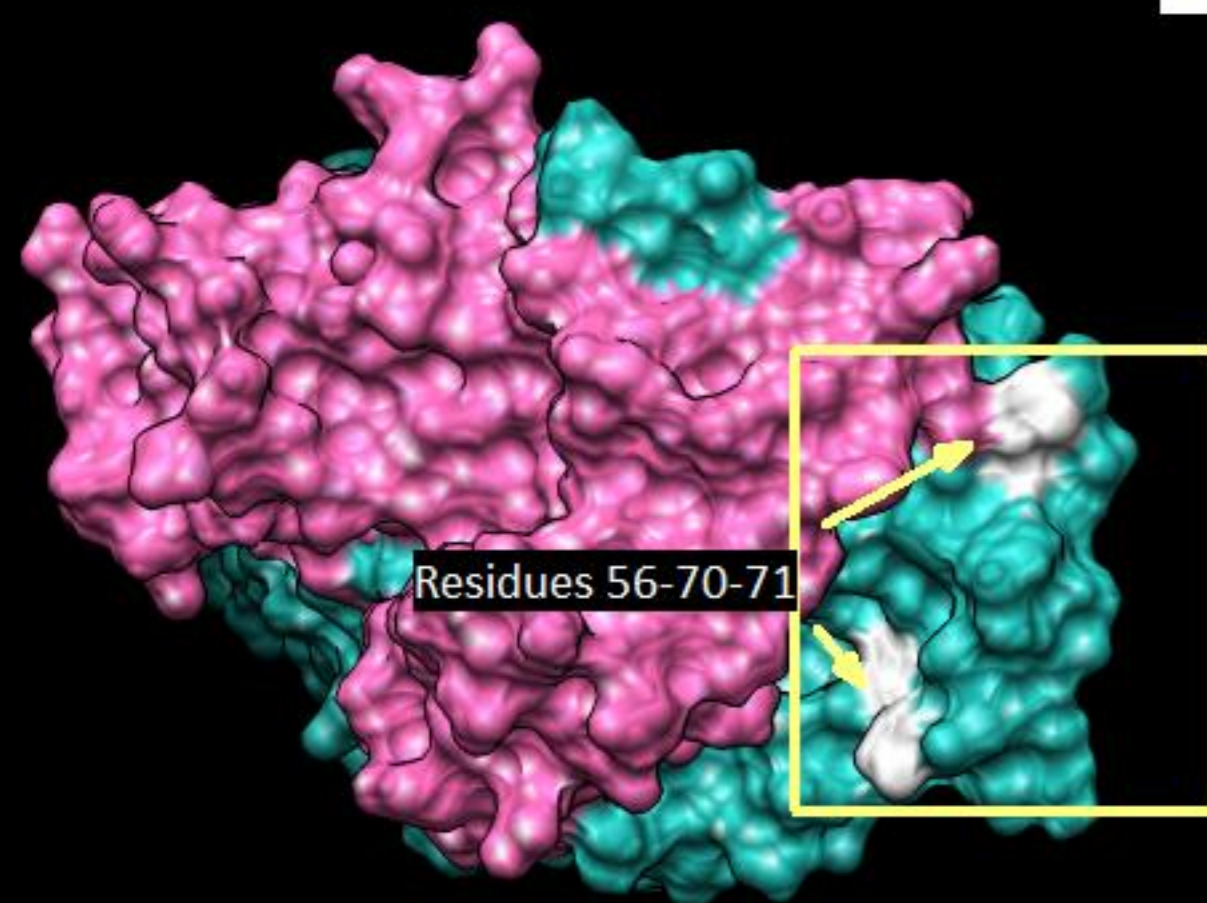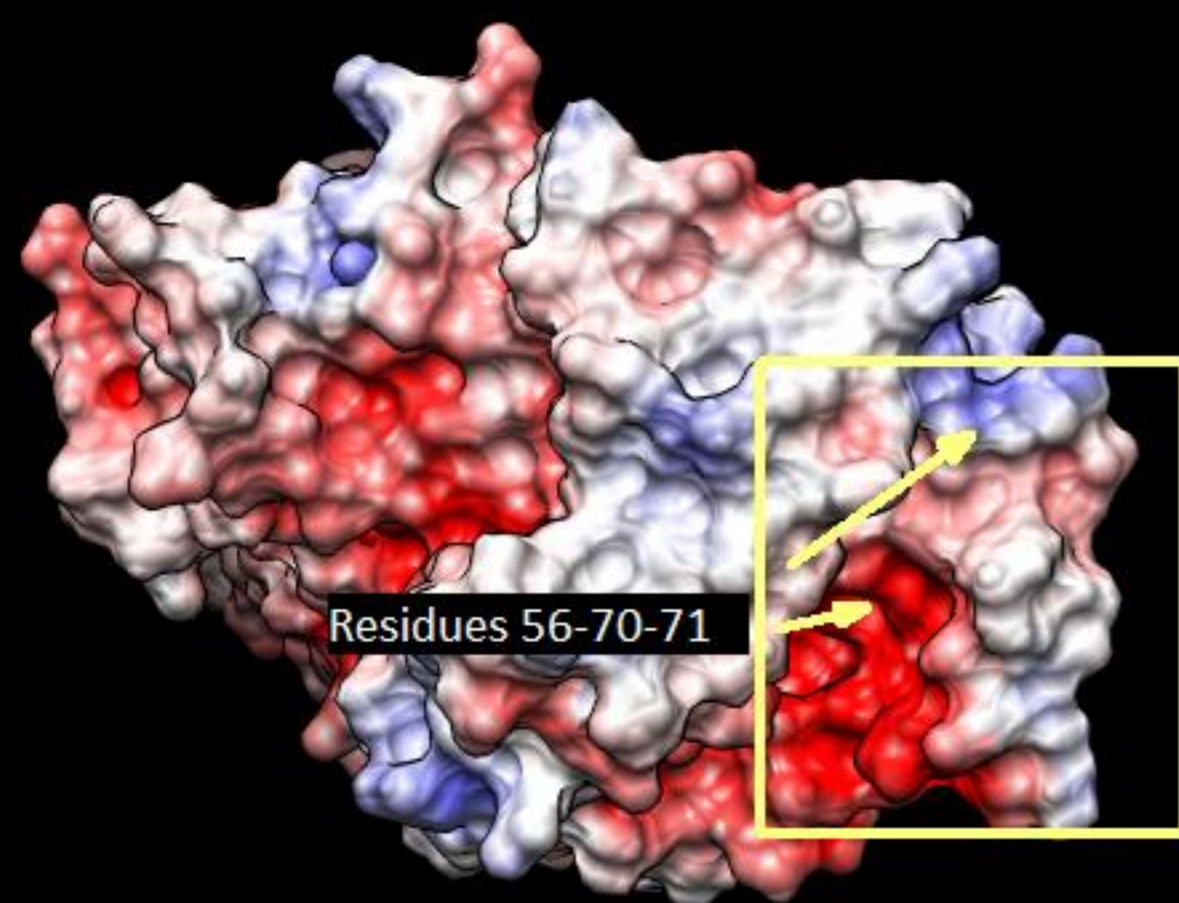

Supplement: S14 Fig — A) The structure and electrostatic potential of HLA-DQB1*04:02. The area within the frame is depicted in expanded form in B and C, and indicates the position of residues 56, 70 and 71 (arrows). All structures were superimposed on HLA-DQB1*04:02 and therefore show the same view. HLA-DQB1 alleles associated with an increased risk of PBC (04:02 and 03:02) are shown in panel B whereas those associated with a protective effect (06:02 and 03:01) are shown in panel C. Negatively charged potentials (less than 5 kT/e) are coloured red, positively charged (greater than 5 kT/e) blue, and neutral potentials (0 kT/e) are coloured white. Linear interpolation was used to produce the colour for surface potentials between these values. (PDF) [file pgen.1007833.s025.pdf]
