# Supplementary material for: Amino acid residues in five separate HLA genes can explain most of the known associations between the MHC and primary biliary cholangitis
Source: PLoS Genet. 2018 Dec 3;14(12):e1007833. doi: 10.1371/journal.pgen.1007833 (PMC6292650; doi:10.1371/journal.pgen.1007833)
Supplement: S2 Fig — Association analysis results for individual amino acids (panels (A) and (B)), classical alleles (panels (C) and (D)) and SNPs (panels (E) and (F)) in the extended MHC region, once the top five amino acids (left panels) or the top five variables (right panels)—which correspond to two amino acids, two SNPs and a classical allele—have been included in the regression model. (PDF) [file pgen.1007833.s013.pdf]

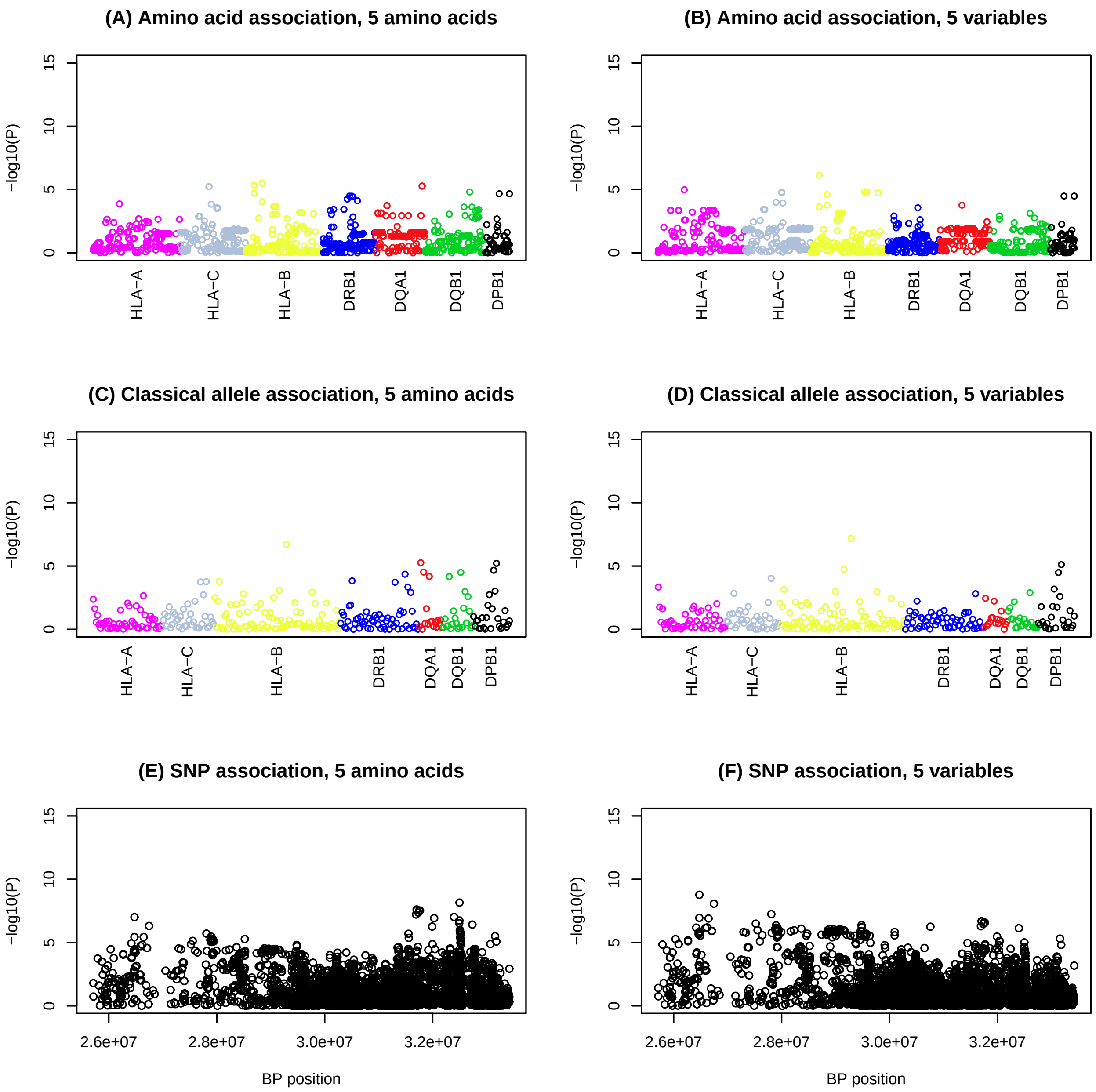
(A) Amino acid association, 5 amino acids
(B) Amino acid association, 5 variables
(C) Classical allele association, 5 amino acids
(D) Classical allele association, 5 variables
(E) SNP association, 5 amino acids
(F) SNP association, 5 variables
−log10(P)
0
5
10
15
HLA−A
HLA−C
HLA−B
DRB1
DQA1
DQB1
DPB1
2.6e+07
2.8e+07
3.0e+07
3.2e+07
BP position
