# Supplementary material for: Amino acid residues in five separate HLA genes can explain most of the known associations between the MHC and primary biliary cholangitis
Source: PLoS Genet. 2018 Dec 3;14(12):e1007833. doi: 10.1371/journal.pgen.1007833 (PMC6292650; doi:10.1371/journal.pgen.1007833)
Supplement: S3 Fig — Association analysis results for individual amino acids while including in the regression model: (A) no other variables; (B) the top five amino acids from stepwise regression; (C) the top five amino acid positions (resulting in multi-df tests at each position) from stepwise regression; (D) the top seven amino acid positions (resulting in multi-df tests at each position) from stepwise regression. (PDF) [file pgen.1007833.s014.pdf]

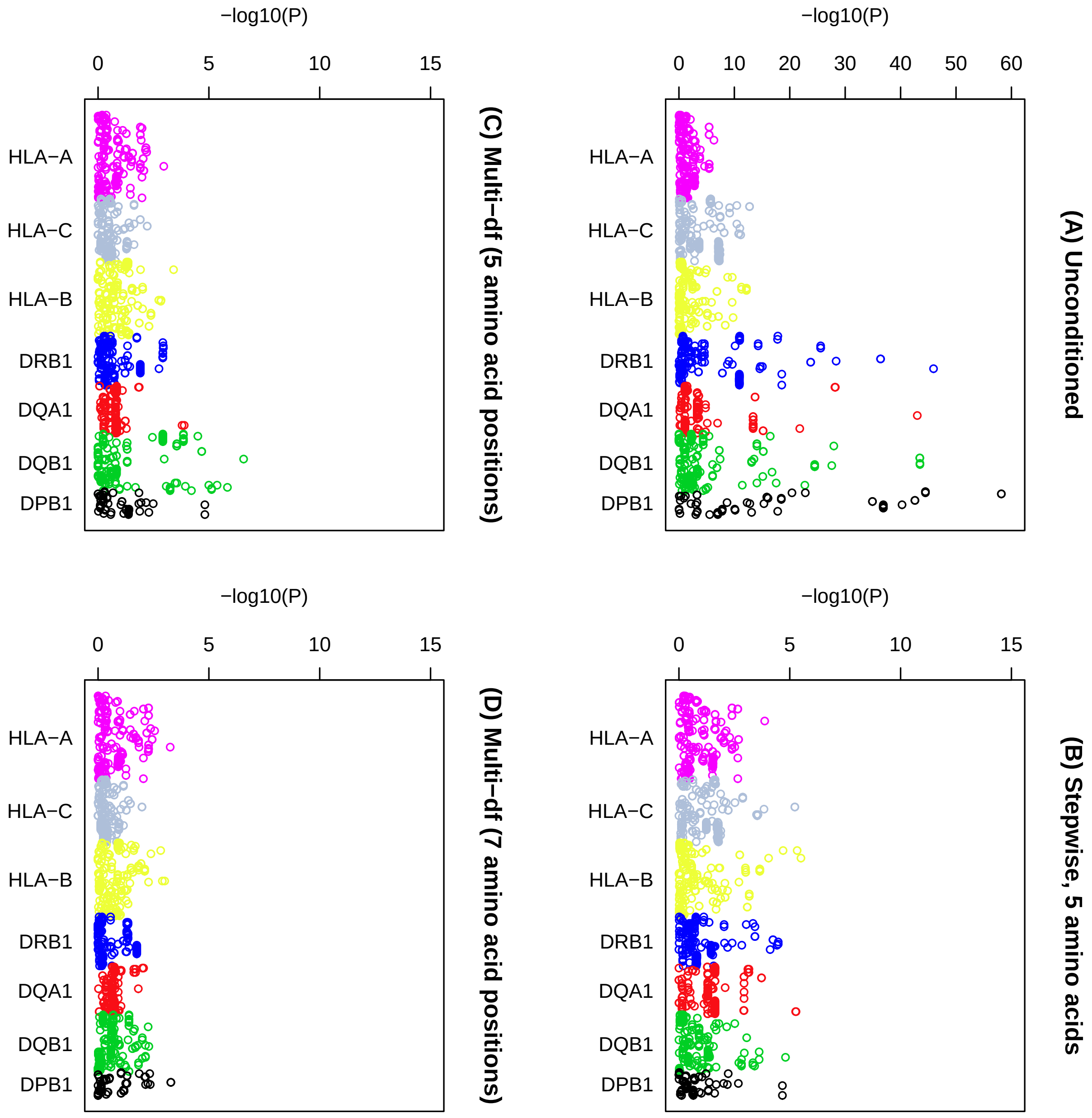

(A) Unconditioned
(B) Stepwise, 5 amino acids
(C) Multi−df (5 amino acid positions)
(D) Multi−df (7 amino acid positions)
−log10(P)
0
5
10
15
20
30
40
50
60
HLA−A
HLA−C
HLA−B
DRB1
DQA1
DQB1
DPB1
