# Supplementary material for: Amino acid residues in five separate HLA genes can explain most of the known associations between the MHC and primary biliary cholangitis
Source: PLoS Genet. 2018 Dec 3;14(12):e1007833. doi: 10.1371/journal.pgen.1007833 (PMC6292650; doi:10.1371/journal.pgen.1007833)
Supplement: S7 Fig — Association analysis results for individual SNPs while including in the regression model: (A) no other variables; (B) the top five amino acids from stepwise regression; (C) the four amino acids in the top model from FINEMAP, when limiting to a maximum of four predictors; (D) the five amino acids in the top model from FINEMAP, when limiting to a maximum of five predictors; (E) the six amino acids in the top model from FINEMAP, when limiting to a maximum of six predictors; (F) the seven amino acids in the top model from FINEMAP, when limiting to a maximum of seven predictors. (PDF) [file pgen.1007833.s018.pdf]

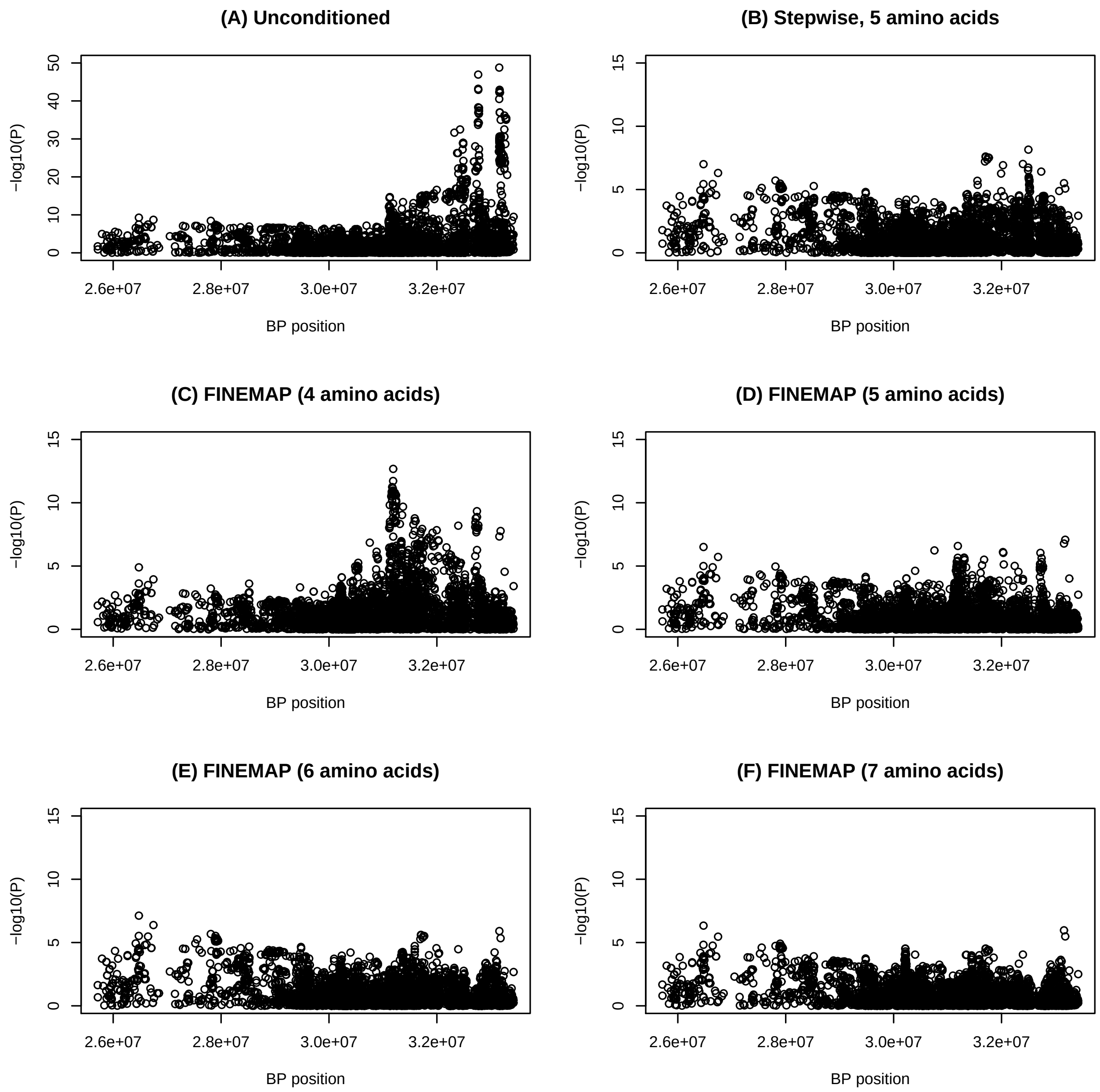
(A) Unconditioned
(B) Stepwise, 5 amino acids
(C) FINEMAP (4 amino acids)
(D) FINEMAP (5 amino acids)
(E) FINEMAP (6 amino acids)
(F) FINEMAP (7 amino acids)
−log10(P)
BP position
2.6e+07
2.8e+07
3.0e+07
3.2e+07
