# Supplementary material for: Amino acid residues in five separate HLA genes can explain most of the known associations between the MHC and primary biliary cholangitis
Source: PLoS Genet. 2018 Dec 3;14(12):e1007833. doi: 10.1371/journal.pgen.1007833 (PMC6292650; doi:10.1371/journal.pgen.1007833)
Supplement: S1 Text — (DOCX) [file pgen.1007833.s026.docx]

**DESCRIPTION OF STEPWISE LOGISTIC REGRESSION ANALYSIS**

***Details of methodology***

Stepwise logistic regression was used to assess the importance of variables while accounting for the effects of other, previously detected, effects. In this analysis, predictor variables encoding an individual’s estimated dosage of the relevant SNP, HLA classical allele or amino acid substitution were included as predictors in a logistic regression equation in a forward stepwise fashion (and were subsequently considered for removal from the model in a backward stepwise fashion). We note that inclusion of an additively coded (dosage) variable on the log odds scale as used in logistic regression is equivalent to assuming multiplicative effects of alleles on the odds scale. At each step, the variable chosen to next enter the model was that for which the difference between the log likelihoods of the current model (with the variable included) and the previous model (without the variable included) was greatest. Selected models were also compared using the Akaike Information Criterion (AIC). Thus, a comparison of models between step *j* and step *j*+1 involved modeling the log odds of disease as a linear function of either *j* or *j*+1 predictors (denoted x_1_, x_2_, x_3_… x*_j_*, x*_j_*_+1_) and comparing the fit of the two models:

ln(p/(1-p))=β_0_+β_1_x_1_+β_2_x_2_+…+β*_j_*x*_j_* versus ln(p/(1-p))=β_0_+β_1_x_1_+β_2_x_2_+…+β*_j_*x*_j_*+β*_j_*_+1_x*_j_*_+1_

(where p corresponds to the probability of being diseased) via comparing either their log likelihoods or their AICs.

For our primary analyses, we did not consider it necessary to include additional predictors such as principal component scores in the regression model to account for possible population stratification, as prior analysis of this UK PBC data set [1-3] has shown little evidence of population stratification (once appropriate QC has been performed to remove outlying individuals). Similarly we did not consider it necessary to include gender as a covariate – even though gender is known to be important in PBC (the disease is more prevalent in women than in men) – as theoretical arguments dictate that inclusion of gender should not bias the results of association tests between disease and genetic factors (outside of the X/Y chromosomes) as gender is not a *confounder* (it is associated with the disease outcome, but not with the genetic predictors). Indeed, it has been shown [4] that inclusion of known covariates such as gender can even reduce power to detect genetic effects in case-control studies. We subsequently investigated the sensitivity of our results to the inclusion (or not) of principal component scores and gender in the regression model, using the top 10 principal component scores calculated from a pruned (by LD) set of SNPs with SNPs in the extended HLA region removed, but found, as expected from theoretical arguments, that this had little impact on the results obtained.

We also investigated the stability of the stepwise selection procedure through a resampling approach motivated by the stability selection procedure of Meinshausen and Buhlmann (2010) [5]. In each of 1000 bootstrap replicates we randomly selected 2/3 of our cases and 2/3 of our controls to form a new case/control data set and applied stepwise regression to select the top 20 amino acid predictors, noting the order and significance of entry of each predictor in each replicate.

***Results of application to PBC data, one degree of freedom (df) models***

Application of the stepwise regression procedure to amino acid residue variables alone resulted in a final model that included nine amino acids when using a stopping threshold of *P*=4.87x10^-5^, or five amino acids when using a more stringent stopping threshold of *P*=1.0x10^-8^ (Table 2). The same five amino acids entered, in the same order (and all with *P*<1.0x10^-8^) when including the top 10 principal component scores as covariates. When including the top 10 principal component scores and gender as covariates, four out of the top five identified amino acids (all still with *P*<1.0x10^-8^) remained the same, with HLA-DQβ1 87F entering the model in preference to the marginally less significant HLA-DQβ1 57D, and with the order of entry slightly altered as follows: (1) HLA-DPβ1 11L/G (*P*=5.70x10^-53^), (2) HLA-DRβ1 74L (*P*=4.67x10^-33^), (3) HLA-DQα1 -13A (*P*=2.34x10^-20^), (4) HLA-DQβ1 87F (*P*=1.18x10^-13^), and (5) HLA-C 156R (*P*=2.23x10^-10^)

The stepwise regression procedure was found to be highly stable in terms of the top amino acid predictors identified. In 1000 bootstrap replicates (each containing 2/3 of our PBC cases and 2/3 of our controls) the top amino acid HLA-DPβ1 11L/G entered as the most significant predictor in 86.7% of replicates (and entered as second in the remaining 13.3% of replicates). The second amino acid to enter was HLA-DRβ1 74L in 83.1% of replicates. The third amino acid to enter was HLA-DQβ1 57D in 51% of replicates (the closest competitor was HLA-DRβ1 67L which entered third in 25.8% of replicates). The fourth amino acid to enter was HLA-C 156R in 33.7% of replicates (the closest competitor was HLA-C 152A which entered fourth in only 10.3% of replicates). The fifth amino acid to enter was HLA-DQα1 -13A in 27.9% of replicates (the closest competitor was HLA-DRβ1 58A which entered fifth in only 10.5% of replicates).

We further examined the relationship between amino acid residues and SNPs or classical HLA alleles by allowing either amino acid residues and/or SNPs and/or classical HLA alleles to enter the stepwise regression model at each step. At each of steps 1-2, an amino acid residue (HLA-DPβ1 11L at step 1 and HLA-DRβ1 74L at step 2) entered the model preferentially in comparison to a SNP or a classical HLA allele. At step 3, a SNP (rs7774434, P=2.78x10^-23^) entered in preference to the top amino acid (HLA-DQB 57D, P=6.42x10^-22^) or the top classical allele (HLA-DQA1*05:05, P=1.18x10^-21^). At step 4, assuming rs7774434 had been entered at step 3, a SNP (rs3130553, P=4.41x10^-14^) again entered in preference to the top amino acid (HLA-DQA -13A, P=1.15x10^-13^) or the top classical allele (HLA-DQA1*05:05, P=9.25x10^-14^). (If HLA-DQB 57D had instead been entered at step 3, then at step 4 the amino acid residue HLA-C 156R (P=2.70x10^-12^) entered in preference to the top SNP (rs3906263, P=1.43x10^-11^) or the top classical allele (HLA-DQA1*05:05, P=1.38x10^-11^)).

At step 5, the top variable for inclusion was the classical HLA allele HLA-DQA1*05:05, which showed marginally stronger association (P=6.52x10^-14^) than the top amino acid residue (HLA-DQα1 -13A, P=8.19x10^-14^) or SNP (rs4639334, P=9.50x10^-14^); once HLA-DQA1*05:05 had entered the model, amino acid residue HLA-DQα1 -13A was no longer significant and vice versa, suggesting that the classical HLA allele and amino acid residue are essentially marking the same effect. Further investigation showed that the molecule encoded by HLA-DQA1*05:05 actually carries a T residue instead of the A residue carried by all other HLA-DQA1 encoded molecules (except for the molecule encoded by HLA-DQA1*05:09, which also carries a T residue but occurs with low probability). Amino acid residue HLA-DQα1 -13T has a marginally less significant stepwise effect than HLA-DQα1 -13A (P=8.19x10^-14^ compared to P=6.52x10^-14^) but effectively these two substitutions at HLA-DQα1 -13 operate in a “yin-yang” fashion, with T (carried on the molecule encoded by HLA-DQA1*05:05) decreasing and A (carried on most other molecules) increasing the odds of disease.

These results illustrate the difficulty of disentangling “causal” from “hitchhiking” effects amongst highly correlated variables such as the amino acid residues, classical alleles and SNPs considered here. We note that, in most cases, the difference in model fit between including the top SNP and the top amino acid or classical allele was relatively small. Given the a priori potential functional role of amino acid substitutions, we found it most natural to focus primarily on variables directly encoding these effects. The fact that, in some instances, inclusion of a SNP provided a slightly better model fit could indicate that the SNP itself is having a functional role (perhaps through a mechanism such as modulation of gene expression) but, equally, could arise from the phenomenon whereby a SNP tags the combined effects of several functional amino acids. In terms of accounting for the overall association in the region, we found the model that included the top 5 amino acids (S2 Figure, left hand panels) performed similarly to the model that included the top 5 variables of any type (S2 Figure, right hand panels).

***Results of application to PBC data, multi-degree of freedom (df) models***

We additionally fitted multi-df models that included predictor variables encoding the effects of all amino acid substitutions at a position simultaneously (the maximum number of such amino acid variants at a position was 8). This analysis strategy investigates the *combined* effects seen at a particular position of the amino acid sequence, rather than the effects of individual specific amino acid residues, and was the preferred strategy of Goyette et al. (2015) [6], although we favour the investigation of individual amino acid residues as providing a more interpretable analysis. The results from forward stepwise regression (using a stopping significance level threshold of *P*=0.000136, representing a Bonferroni-corrected threshold of 0.05, allowing for 368 amino acid positions tested) are shown in S2 Table; this resulted in a final model that included residues at eight amino acid positions, however the second position to enter (position 71 of HLA-DQβ1) was subsequently removed via a backward step. The results from this multi-df procedure show reasonable concordance with the results seen when considering individual amino acid substitutions (Table 2), with positions 11 and 215 of HLA-DPβ1, position 45 of HLA-B and position 156 of HLA-C coming up as important players. However, some new positions are also implicated by the multi-df analysis, which allows the entry of multiple predictors at each step. The degree to which these multiple sets of predictors can account for the observed amino acid, SNP and classical HLA allele associations in the region is shown in Figures S3, S4 and S5. In some cases the association is slightly better accounted for by the multiple sets of predictors (Figures S3, S4, S5; lower panels) than by the five individual amino acid residues previously identified (Figures S3, S4, S5; upper right panel), however this comes at the expense of a larger number of degrees of freedom and an arguably less interpretable model. Given that the five individual amino acid residues previously identified do almost as well at accounting for the association as do the multi-df models, overall we prefer the five amino acid model identified through stepwise regression as representing the most parsimonious solution.

**References for Text S1**

1. Cordell HJ, Han Y, Mells GF, Li Y, Hirschfield GM, Greene CS, et al. International genome-wide meta-analysis identifies new primary biliary cirrhosis risk loci and targetable pathogenic pathways. Nat Commun. 2015;6:8019. doi: 10.1038/ncomms9019. PubMed PMID: 26394269; PubMed Central PMCID: PMC4580981.

2. Liu JZ, Almarri MA, Gaffney DJ, Mells GF, Jostins L, Cordell HJ, et al. Dense fine-mapping study identifies new susceptibility loci for primary biliary cirrhosis. Nature genetics. 2012;44(10):1137-41. doi: 10.1038/ng.2395. PubMed PMID: 22961000; PubMed Central PMCID: PMC3459817.

3. Mells GF, Floyd JA, Morley KI, Cordell HJ, Franklin CS, Shin SY, et al. Genome-wide association study identifies 12 new susceptibility loci for primary biliary cirrhosis. Nature genetics. 2011;43(4):329-32. doi: 10.1038/ng.789. PubMed PMID: 21399635; PubMed Central PMCID: PMC3071550.

4. Pirinen M, Donnelly P, Spencer CC. Including known covariates can reduce power to detect genetic effects in case-control studies. Nature genetics. 2012;44(8):848-51. doi: 10.1038/ng.2346. PubMed PMID: 22820511.

5. Meinshausen N, Buhlmann P. Stability selection. Journal of the Royal Statistical Society Series B-Statistical Methodology. 2010;72:417-73. PubMed PMID: WOS:000280638400003.

6. Goyette P, Boucher G, Mallon D, Ellinghaus E, Jostins L, Huang H, et al. High-density mapping of the MHC identifies a shared role for HLA-DRB1*01:03 in inflammatory bowel diseases and heterozygous advantage in ulcerative colitis. Nature genetics. 2015;47(2):172-9. Epub 2015/01/07. doi: 10.1038/ng.3176. PubMed PMID: 25559196; PubMed Central PMCID: PMCPMC4310771.
