# Supplementary material for: Amino acid residues in five separate HLA genes can explain most of the known associations between the MHC and primary biliary cholangitis
Source: PLoS Genet. 2018 Dec 3;14(12):e1007833. doi: 10.1371/journal.pgen.1007833 (PMC6292650; doi:10.1371/journal.pgen.1007833)
Supplement: S2 Text — (DOCX) [file pgen.1007833.s027.docx]

**DESCRIPTION OF DOMINANT, RECESSIVE, GENOTYPIC AND INTERACTION MODELS**

***Details of methodology***

Logistic regression and stepwise logistic regression analysis of imputed dosages of classical HLA alleles (and the resulting implied dosages of amino acid substitutions) makes the simplifying assumption that each allele or amino acid substitution acts multiplicatively with respect to increasing or decreasing the odds of disease (or, equivalently, acts additively with respect to altering the log odds of disease); even if this one degree-of-freedom model is not a true representation of reality, it generally provides good power for testing the null hypothesis of no association [1]. However, non-multiplicative effects at HLA have been observed in other autoimmune diseases [2]. For each of the classical HLA alleles (Table 1) and amino acid substitutions (Table 2) identified using the multiplicative allelic model, we therefore tested whether a two degree-of-freedom genotypic model provided a better fit to the data, and whether the data could be better explained by a dominant model (signifying presence/absence of the relevant allele or amino acid substitution) or by a recessive model (signifying presence/absence of two copies of the relevant allele or amino acid substitution) than by a multiplicative model. The preferred model in each case was chosen as that with the lowest Akaike Information Criterion (AIC).

For fitting dominant models, we defined rounded dosage variables (rounded to take the value of the closest integer (0, 1, 2)) and we then recoded rounded dosages that equalled either 1 or 2 as 1 to use as predictors in logistic regression analysis. For fitting recessive models, we used indicators of whether the rounded dosages equalled 2 as predictors in logistic regression analysis. For fitting genotypic models, we declared the original rounded dosage variables (0, 1, 2) as “factors” in the logistic regression analysis. This is equivalent to including two predictor variables simultaneously in the regression model, one coded as (0, 1, 2) to model additive (dosage) effects and one coded as (0, 1, 0) to model “dominance” effects.

Pairwise interactions were modelled by treating the rounded dosage variables (0, 1, 2) as “factors” in the logistic regression analysis, and then fitting both main effects and interaction terms. This is equivalent to constructing variables that code for the 9 possible pairwise combinations of classical HLA alleles or amino acid residues, and comparing the fit of the 9-parameter saturated model to that of a 5-parameter model where only the main effects of the two variables are included. This generates a 4 degree-of-freedom interaction test that compares models

ln(p/(1-p))=β_0_+β_1_I[x_1_=1]+β_2_I[x_1_=2]+γ_1_I[x_2_=1]+γ_2_I[x_2_=2]

versus

ln(p/(1-p))=

β_0_+β_1_I[x_1_=1]+β_2_I[x_1_=2]+γ_1_I[x_2_=1]+γ_2_I[x_2_=2]+δ_11_I[x_1_=1]I[x_2_=1]

+ δ_12_I[x_1_=1]I[x_2_=2]+ δ_21_I[x_1_=2]I[x_2_=1]+ δ_22_I[x_1_=2]I[x_2_=2]

via a comparison of their log likelihoods, where x_1_ and x_2_ are the rounded dosage variables and I is an indicator function.

***Results of application to PBC data***

For each of the classical HLA alleles (Table 1) and amino acid substitutions (Table 2) identified using the 1df multiplicative allelic model, we tested whether the data could be better explained by a 2df genotypic model or by a 1df dominant or a 1df recessive model. For the classical HLA alleles (S6 Table) the preferred model was generally either multiplicative or dominant, and there was generally little difference in fit between the multiplicative and dominant models, suggesting insufficient data (in particular insufficient observations with two copies of the allele in question) as to be able to distinguish between these two scenarios. For the amino acid residues (S7 Table), the multiplicative allelic model provided the best fit in all cases except for HLA-DRβ1 74L which showed weak evidence (P=0.015) against a multiplicative model and for which a dominant model fitted slightly better (AICs 12629.77 for multiplicative allelic versus 12624.14 for dominant). Only five cases and seven controls were homozygous at HLA-DRβ1 74L, meaning that there was very little difference between a dominantly coded and a multiplicatively coded predictor variable; use of the dominant coding did not therefore have any impact on the subsequent entry of variables in the stepwise regression procedure.

We additionally performed pairwise interaction analysis to investigate whether particular *combinations* of classical HLA alleles or amino acid residues led to increased or reduced risks (over and above their individual multiplicative effects) but found no evidence of any significant interactions, once Bonferroni correction had been made for the number of tests performed. Such interactions, had they existed, could potentially have signified that particular classical HLA alleles and/or amino acid substitutions operate as heterogeneous causes of disease, but we found no evidence to support this hypothesis. We note, however, that, given the strong linkage disequilibrium seen in the HLA region, much larger sample sizes than are available here would probably be required in order to definitively address this question.

**References for Text S2**

1. Iles MM. The impact of incomplete linkage disequilibrium and genetic model choice on the analysis and interpretation of genome-wide association studies. Ann Hum Genet. 2010;74(4):375-9. doi: 10.1111/j.1469-1809.2010.00579.x. PubMed PMID: 20597907; PubMed Central PMCID: PMC2905613.

2. Lenz TL, Deutsch AJ, Han B, Hu X, Okada Y, Eyre S, et al. Widespread non-additive and interaction effects within HLA loci modulate the risk of autoimmune diseases. Nature genetics. 2015;47(9):1085-90. doi: 10.1038/ng.3379. PubMed PMID: 26258845; PubMed Central PMCID: PMC4552599.
