# Supplementary material for: Amino acid residues in five separate HLA genes can explain most of the known associations between the MHC and primary biliary cholangitis
Source: PLoS Genet. 2018 Dec 3;14(12):e1007833. doi: 10.1371/journal.pgen.1007833 (PMC6292650; doi:10.1371/journal.pgen.1007833)
Supplement: S3 Text — (DOCX) [file pgen.1007833.s028.docx]

**DETAILS OF HLA IMPUTATION**

***Imputation with HIBAG:***

Seven classical HLA alleles (HLA-A, HLA-B, HLA-C, HLA-DQA1, HLA-DQB1, HLA-DRB1 and HLA-DPB1) were imputed using the R software package HIBAG [1], using its hg18 ImmunoChip-specific European “pre-fit classifier set” of 3200 SNPs available in the HLARES reference population, 2642 of which were present in our data set. PLINK-format data [2] were first converted to the HIBAG format using HIBAG’s “hlaBED2Geno” function against the hg18 assembly. Each HLA locus was then separately imputed using the HIBAG “predict” function. Case-control analysis was carried out via logistic regression in R with predictors corresponding to HIBAG’s “best-guess” genotypes (provided they had posterior probability > 0.8). The expected dosage of each allele was also calculated by summing for each individual the posterior probabilities of the imputed classical alleles (i.e. the sum of all posterior probabilities for heterozygous genotypes containing that allele, plus twice the posterior probability of the homozygous genotype call), and these were used as predictors in a logistic regression analysis in order to account for genotype uncertainty after imputation.

***Imputation with SNP2HLA:***

Imputation of eight classical HLA alleles (HLA-A, HLA-B, HLA-C, HLA-DQA1, HLA-DQB1, HLA-DRB1, HLA-DPA and HLA-DPB1) was carried out using the software package SNP2HLA [3], which uses a maximum of 8961 SNPs available from the Type 1 Diabetes Genetics Consortium (T1DGC) reference panel. (It is unclear from the output generated how many of the 7848 SNPs we provided were actually used for imputation by the program). Due to the large memory requirement of SNP2HLA, the PBC data set was first split into five sets of 2000 samples plus one of 1375 samples. HLA imputation was then carried out using SNP2HLA with the maximum java heap size set to 15000 MB and the marker window size set at 1000. Association testing of the imputed HLA dosages via PLINK’s logistic regression function was implemented automatically within SNP2HLA.

***Imputation with HLA*IMP:02:***

Imputation of 10 classical HLA alleles (HLA-A, HLA-B, HLA-C, HLA-DPB1, HLA-DQA1, HLA-DQB1, HLA-DRB1, HLA-DRB3, HLA-DRB4 and HLA-DRB5) was carried out using the software package HLA*IMP:02 [4]. The data was first converted from PLINK-format genotypes to OxfordHLA standard format via the HLA*IMP Front End Client. Within the Quality Control tab of this program, missing and non-HLA data was then removed from the formatted data, using a missing data threshold of 0.20. The remaining genotypes were then aligned to HapMap3 chromosome 6 and submitted to the HLA*IMP:02 servers for imputation using the European training set. The resultant imputations were downloaded and dosages of individual alleles were calculated from the genotype posterior probabilities, as detailed above for the output of HIBAG. Association between HLA allele dosage and PBC status was tested using logistic regression within R.

For HLA*IMP:02, like SNP2HLA, it is unclear from the output generated how many of the SNPs that we provided were actually used for imputation by the program.

***Imputation with HLA*IMP:03:***

Imputation of 10 classical HLA alleles (HLA-A, HLA-B, HLA-C, HLA-DPB1, HLA-DQA1, HLA-DQB1, HLA-DRB1, HLA-DRB3, HLA-DRB4 and HLA-DRB5) was carried out using a development version of the software package HLA*IMP:03 [5]. The data was first converted from hg18 to hg19 coordinates via the LiftOver tool (<https://genome.ucsc.edu>) and remapped to the positive strand where necessary, before extracting SNPs on chromosome 6 from coordinates 20,000,000 to 40,000,000 bp. These were then converted from PLINK-format genotypes to vcf format and submitted to the Michigan Imputation Server for haplotype phasing and SNP imputation. The reference panel was set to 1000G Phase 3 v5 and the Eagle [6] algorithm selected to provide phased output. The downloaded results of the imputation were then converted to Oxford HAPS/SAMPLE format, retaining only the SNPs required by HLA*IMP:03. As the ImmunoChip is not specifically supported by HLA*IMP:03, “all SNPs in the reference panel” were selected as the SNP array and the HLA*IMP multi-Population Panel (MPP), release 1 used as the reference panel. File sizes are limited to 100 MB and so our 266 MB dataset was broken into 3 subsets consisting of one batch of 2861 cases and two equally split batches of 4257 controls, all genotyped at 6114 SNPs. The resulting imputations were downloaded and recombined, with case-control analysis carried out via logistic regression in R with predictors corresponding to the most likely genotype.

***Imputation of HLA amino acid substitutions***

We used the probabilistically inferred HLA alleles from HIBAG to probabilistically infer the amino acid residue at each amino acid position, using peptide sequences downloaded from the IMGT/HLA Database. Allele-dependent sites of amino acid variation in the peptide were identified for each of the six HLA molecules. For each amino acid variant, the expected dosage for each individual was calculated by summing the previously-calculated dosages of those imputed classical alleles that carried the variant. We then used logistic regression (the *glm()* function) in R to test the dosage of each imputed amino acid variant for association with PBC. We also carried out a multi-allelic (multi-degree of freedom (df)) analysis by including predictor variables encoding the effects of all amino acids at a position included simultaneously (the maximum number of such amino acid variants at a position was 8). We additionally compared our results to those directly obtained using the package SNP2HLA.

**References for Text S3**

1. Zheng X, Shen J, Cox C, Wakefield JC, Ehm MG, Nelson MR, et al. HIBAG-HLA genotype imputation with attribute bagging. Pharmacogenomics J. 2014;14(2):192-200. doi: 10.1038/tpj.2013.18. PubMed PMID: WOS:000333665400013.

2. Purcell S, Neale B, Todd-Brown K, Thomas L, Ferreira MAR, Bender D, et al. PLINK: A tool set for whole-genome association and population-based linkage analyses. American journal of human genetics. 2007;81(3):559-75. doi: 10.1086/519795. PubMed PMID: WOS:000249128200012.

3. Jia X, Han B, Onengut-Gumuscu S, Chen WM, Concannon PJ, Rich SS, et al. Imputing amino acid polymorphisms in human leukocyte antigens. PLoS One. 2013;8(6):e64683. doi: 10.1371/journal.pone.0064683. PubMed PMID: 23762245; PubMed Central PMCID: PMC3675122.

4. Dilthey AT, Moutsianas L, Leslie S, McVean G. HLA*IMP--an integrated framework for imputing classical HLA alleles from SNP genotypes. Bioinformatics. 2011;27(7):968-72. doi: 10.1093/bioinformatics/btr061. PubMed PMID: 21300701; PubMed Central PMCID: PMC3065693.

5. Motyer A, Vukcevic D, Dilthey A, Donnelly P, McVean G, Leslie S. Practical Use of Methods for Imputation of HLA Alleles from SNP Genotype Data. BioRxiv (Cold Spring Harbor Labs Journals, 2014). 2016. doi: <https://doi.org/10.1101/091009>.

6. Loh PR, Danecek P, Palamara PF, Fuchsberger C, Y AR, H KF, et al. Reference-based phasing using the Haplotype Reference Consortium panel. Nature genetics. 2016;48(11):1443-8. Epub 2016/10/28. doi: 10.1038/ng.3679. PubMed PMID: 27694958; PubMed Central PMCID: PMCPMC5096458.
